# Programmatic mapping and population size estimation of key population in India: Method and findings

**DOI:** 10.1371/journal.pgph.0004475

**Published:** 2025-05-07

**Authors:** Pradeep Kumar, Chinmoyee Das, Bhawani Singh Khushwaha, Saiprasad P. Bhavsar, Shantanu Kumar Purohit, Arvind Kumar, Subrata Biswas, Nidhi Priyam, Lalit Singh Kharayat, Shajan Mathew, Akhilesh Srivastava, Jyotsana Pal, Shreena Ramanathan, Abhina Aher, Deepika Srivastava Joshi, Rajatashuvra Adhikary, Shajy Isac, H. Sanayaima Devi, P. V. M. Lakshmi, Elangovan Arumugam, Sanjay K. Rai, Sheela V. Godbole, S. K. Singh, Himanshu K. Chaturvedi, Shanta Dutta, Shashi Kant, Dandu Chandra Sekhar Reddy, Sanjay Mehendale, Shobini Rajan

**Affiliations:** 1 National AIDS Control Organization, Ministry of Health and Family Welfare, New Delhi, India; 2 UNAIDS, Delhi, India; 3 USAID, Delhi, India; 4 WHO, Delhi, India; 5 India Health Action Trust, Delhi, India; 6 Regional Institute of Medical Sciences, Imphal, India; 7 Postgraduate Institute of Medical Education and Research, Chandigarh, India; 8 Indian Council of Medical Research, National Institute of Epidemiology, Chennai, India; 9 All India Institute of Medical Sciences, New Delhi, India; 10 Indian Council of Medical Research, National AIDS Research Institute, Pune, India; 11 International Institute for Population Sciences, Mumbai, India; 12 Indian Council of Medical Research, National Institute of Medical Statistics, New Delhi, India; 13 Indian Council of Medical Research, National Institute of Cholera and Enteric Diseases, Kolkata, India; 14 Institute of Medical Sciences, Banaras Hindu University, Varanasi, India; 15 PD Hinduja Hospital and Medical Research Center, Mumbai, India; Institute of Public Health Bengaluru, INDIA

## Abstract

India has the world’s second-largest HIV burden. Key populations of female sex workers (FSW), men who have sex with men (MSM), hijra/transgender (H/TG) people, and people who inject drugs (PWID), are disproportionately affected by the HIV epidemic. A community-led programmatic mapping and population size estimation (PMPSE) was carried out in 651 districts of 32 States and Union Territories of India. The goal was to identify the hotspots, network operators, and estimate the size of key population groups. This involved documenting the known hotspots, visiting them for rapid field assessment through key informants’/ network operators interviews, and identifying additional hotspots/ network operators through the snow-balling approach from the existing hotspots. For each identified hotspot, network operator, and village, size of each key population group was estimated after adjusting for the duplications and overlaps. These estimates were then aggregated to arrive at district, State, and ultimately national-level estimates. PMPSE estimated a total of 9,95,499 (9,02,277–10,88,712) FSWs, 3,51,020 (3,13,860–3,88,175) MSM, 2,88,717 (2,53,024-3,24,407) PWIDs, and 96,193 (85,206-1,07,174) H/TG individuals. The number of FSWs per 1000 adult women in different States/Union Territories (UT) varied from 0.34 to 17.25; MSM estimates ranged from 0.07 to 7.35 per 1000 adult men, H/TG persons ranged from 0.03 to 2.75 per 1000 adult men, and PWIDs ranged from 0.01 to 31.30 per 1000 adult men. Additionally, approximately 14% of FSWs, 7% of MSM, and 8% of H/TG individuals were estimated to operate exclusively through network operators. The community-led PMPSE has updated the size estimates for FSWs, MSM, PWIDs, and H/TG individuals at a granular level. This approach has emphatically quantified the presence of network operators. The methodological simplicity of the present round of PMPSE is likely to encourage and facilitate its periodic implementation for better tracking of population level changes in HIV burden based on more reliable denominators.

## Introduction

Despite the relatively low overall adult HIV prevalence of 0.20% (0.17-0.25) in 2023, The human immunodeficiency virus (HIV) epidemic in India remains a public health challenge [1,2]. With an estimated 2.54 million (2.17-3.04) people living with HIV (PLHIV), India ranks as the country with the second largest population of PLHIV globally [3]. The National AIDS and STD Control Programme (NACP) has successfully responded in restricting the epidemic. Through its comprehensive and pointed initiatives, nearly 44% reduction in new HIV infections and a remarkable 79% decline in AIDS-related deaths from 2010 to 2023 has been estimated in India. Despite the significant success achieved so far, there is no place for complacency and the program implementation must continue with equal vigour and energy in the years to come [4].

The HIV prevalence among key populations (KPs) remains significantly higher than the overall adult prevalence [5]. In stark contrast to the overall adult HIV prevalence of 0.20%, the observed HIV prevalence among KPs ranged from 1.85% to 9.03% in 2021, which was 9–43 times higher than the national adult prevalence. Specifically, the prevalence was 1.85% (1.75-1.96) among female sex workers (FSW), 1.93% (1.75-2.12) among inmates in central jails, 3.26% (3.03-3.48) among men who have sex with men (MSM), 3.78% (3.24-4.33) among hijra/transgender (H/TG) persons, and 9.03% (8.69-9.37) among people who inject drugs (PWID).

Mapping and population size estimation are pivotal to an evidence-based response to HIV/AIDS in settings with concentrated epidemics [6,7]. KP size estimates are crucial for assessing the magnitude of the response required for adequate provision of services preventing HIV infection. These estimates inform HIV response planning, target setting, and resource allocation, and provide denominators for monitoring and evaluating program outcomes and impacts.

Recognizing the necessity for robust key population (KP) size estimates, the mid-term appraisal (MTA) of the National AIDS Control Programme Phase IV (NACP-IV) in India recommended development of a systematic roadmap for size estimation of key populations in the country [8]. This recommendation was supported by the Expert Consultation on HIV Surveillance and Estimations in India in 2016 [9]. India’s National Strategic Plan (2017–2024) also highlighted the importance of periodic updating of key population size estimates to facilitate strategic planning, costing, monitoring, reporting, and evaluation as part of HIV/AIDS prevention and control in India [10].

A White Paper on Population Size Estimation (PSE) was commissioned under the NACP to evaluate various methodologies and recommend an appropriate approach for PSE in India. The White Paper recommended integration of mapping and population size estimations with established peer-led intervention platforms for KPs as a sustainable approach enabling its periodic implementation [11]. This method, termed as programmatic mapping and population size estimation (PMPSE) due to its utilization of established and existing programme implementation and monitoring structures and mechanisms, provides an opportunity to get periodic updates on KP size estimates. Consequently, this approach would enable evidence-based expansion recalibration of interventions.

PMPSE is a sustainable and systematic method designed to map congregation/ solicitation locations (hotspots) of key populations as well as estimate of their size at each hotspot. Globally, PMPSE has been recommended to inform program implementation effectively [12,13]. By aiming to map each hotspot, PMPSE proves invaluable in informing program design, monitoring program efficacy, and guiding programs to provide tailored responses in the scenarios of evolving dynamics.

The first round of the PMPSE was conducted in India in 2020–2022. This paper presents the methodology and findings from the first round of the PMPSE conducted under NACP in India.

## Methods

### Overview

The first round of PMPSE was implemented in 651 districts of India (out of total 763 districts) for FSW, MSM, PWIDs, and H/TG population groups. PMPSE was implemented by employing established mechanisms for routine service delivery and program monitoring for KPs under NACP [14]. Method and tools for PMPSE was first piloted in 5 districts of 4 States (Andhra Pradesh, Mizoram, Chhattisgarh and Uttar Pradesh). This multicentric pilot was to be designed to test the method in a variety of scenarios so that we can understand the strengthens and limitations of this method in a variety of scenarios. The successful implementation of the pilot led to recommendation for nation-wide implementation following PMPSE approach.

KPs are directly covered under NACP through two approaches comprising Targeted Interventions (TI) and Link Workers Scheme (LWS) [15,16]. TIs under NACP are implemented through Non-Government Organisations (NGO)/Community-based Organisations (CBO). TI NGOs/CBOs engages peers from the community (FSW/MSM/PWID or H/TG people) to facilitate offering of comprehensive package of services. Link Workers Scheme provides defined package of services to KPs and other vulnerable population in most populous 100 villages of select districts through Cluster Link Workers (CLWs) identified from the same locality. Data collection under PMPSE was done using Peer Educators (PEs, persons from the KP groups who works with her/ his colleagues to influence attitude and behaviour change) and CLWs engaged by TIs and LWS for delivery of comprehensive package of services for the community. Data was collected during January 2020 to November 2022. Overall, 597 districts were covered through TIs and 146 districts were covered through LWS.

Community Advisory Boards (CAB), constituted in each of the implementation districts, monitored the field activities that included understanding and addressing the community concerns. CAB’s members included KPs, community-level gatekeepers as well as other related-stakeholders. CAB also reviewed the draft findings and provided the local perspectives for data generated for their districts.

The PMPSE mapped the hotspots (specific locations where KP solicit/congregate) in urban/peri-urban areas and estimated KP size through group discussions of key informants (aged 18 years or older) at each of the hotspots. Key informants at hotspots were primarily KPs and community gate keepers. PMPSE at hotspots captured information on KPs who visited these locations. For LWS villages, CLWs provided the consolidated number of KPs being covered by them.

There is growing evidence of the increasing role of persons -managed networks in the solicitation and congregation dynamics of key populations (KPs) [17–20]. KPs who are part of these networks may or may not visit traditional hotspots. Recognizing the evolving operational dynamics, PMPSE also mapped such network operators—individuals with whom groups of KPs are linked for various purposes including soliciting—and interviewed them to estimate the size of the KPs associated with them. The PMPSE defined network operators as individuals from key population groups or others who facilitate FSW, or MSM or H/TG people in seeking a partner [14]. For PWIDs, it referred to persons, who may or may not be injecting themselves, but facilitated the PWIDs to get drugs by sharing information related to drug availability through their networks. Key population can contact them, and vice-versa, via phone or message (SMS, WhatsApp etc) to connect with sexual/injecting partners.

Data collection was done using specific tools for hotspots, network operators and LWS villages (Supplementary Information/Reference to the operational manual). Each tool had specific questions to explore and account for the overlap. The hotspot information format collected information on the minimum and maximum number of KPs frequenting it. This maximum and minimum number was used to inform the lower and upper bound of the size estimates. The estimates, generated after adjusting for overlaps, were reviewed by institutional structures at district, State and national level. The final approval for the findings was provided by the Technical Resource Group (Surveillance & Epidemiology) under NACP.

Overall, 651 districts across 32 States/UTs in India were covered under PMPSE (Table 1). PMPSE was not implemented in the UT of Andaman & Nicobar Islands (3 districts), Dadra and Nagar Haveli and Daman and Diu (3 districts), and Lakshadweep (1 district). Among the 105 districts where the PMPSE program was not implemented, Bihar and Chhattisgarh in eastern India accounted for the highest number of uncovered districts, with 20 and 10 districts respectively. North-eastern States of Arunachal Pradesh, Assam, Manipur, Meghalaya, Mizoram, Nagaland and Sikkim accounted for another 33 districts where PMPSE was not implemented. PMPSE was not implemented in 7 districts of Tamil Nadu (a State in southern region) followed by five districts in +Uttar Pradesh (a State in northern India).

**Table 1:** State/UT-wise number of districts covered under the PMPSE 2020–22.

State/UT	Total Districts	Districts Covered under PMPSE	Districts with TI	Districts having LWS
Andaman & Nicobar Islands	3	–	–	–
Andhra Pradesh	26	22	22	13
Arunachal Pradesh	25	16	16	–
Assam	33	26	26	–
Bihar	38	18	18	5
Chandigarh	1	1	1	–
Chhattisgarh	33	23	20	4
Dadra and Nagar Haveli and Daman and Diu	3	–	–	–
Delhi	11	11	11	–
Goa	2	2	2	–
Gujarat	33	33	29	14
Haryana	22	20	17	–
Himachal Pradesh	12	10	10	–
Jammu And Kashmir	22	19	7	–
Jharkhand	24	20	20	–
Karnataka	31	30	30	9
Kerala	14	14	14	–
Lakshadweep	1	0	0	–
Madhya Pradesh	52	50	50	10
Maharashtra	36	32	30	19
Manipur	16	14	14	14
Meghalaya	12	8	7	2
Mizoram	11	8	8	8
Nagaland	16	12	12	–
Odisha	30	30	24	6
Puducherry	4	4	3	–
Punjab	23	21	21	7
Rajasthan	33	31	22	–
Sikkim	6	2	2	–
Tamil Nadu	38	31	29	12
Telangana	33	33	33	29
Tripura	8	8	8	5
Uttar Pradesh	75	70	62	7
Uttarakhand	13	11	7	–
West Bengal	23	21	21	14
**India**	**763**	**651**	**596**	**178**

PMPSE implementation included 596 districts having targeted interventions or link worker scheme. Besides, PMPSE was also implemented in 54 districts which did not have TI or LWS but were epidemiologically important based on the local intelligence informed by HIV positivity, self-reported route of transmission, known presence of pockets of KP etc. In these districts, community liaisons were selected with the aid of key informants to facilitate PMPSE implementation. TIs/LWS personnel from neighbouring districts were also engaged to support PMPSE implementation in these districts.

### Mapping and size estimation at hotspots

PMPSE at hotspots was implemented in two sequential phases. The first phase included listing of all hotspots in the given district. Additionally, information on locations of hotspots was also collected by interviewing key informants after conducting the first meeting of the CAB. This list was compared with the list of hotspots available with TI NGOs/CBOs and a comprehensive master list of hotspots was developed.

In the second phase, Rapid Field Assessment (RFA) was undertaken. In RFA, each hotspot in the master list was visited and information was collected from three to six key informants (primary and secondary), to characterise the hotspot in terms of its type, the nature of activities at the hotspot and the size of population associated with it. Outcome of interviews, in terms of hotspot type, size estimates, operational days, peak time and programme coverage status, was captured in Hotspot Information Format (HIF) for each hotspot (S1 Appendix). If a hotspot was used by more than one KP group, RFA was implemented separately for each of the KP group separately and HIF was filled for each KP group separately.

At each of the hotspot, during RFA, key informants were asked to list other hotspots in their locality where KPs solicit/congregate. The list mentioned by key informants was then compared with the master list. If a hotspot mentioned by key informants was not already included in the master list, it was subsequently added and covered under RFA. This iterative process ensured that the universe of hotspots under master list kept on increasing and all hotspots known to the key informants were covered under PMPSE.

### Mapping and size estimation for network operators

Similar to physical hotspots, PMPSE with network operators was also done in sequential steps. The first phase included identification of an initial set of network operators by having discussions with stakeholders who were aware of the network dynamics in the community. Once this first set of network operators was developed, they were contacted to collect information as per the prescribed Network Operator Format (NOF) (S2 Appendix). This process required multiple contacts with potential respondents to build rapport, ensure confidentiality, and seek their support after explaining the objectives of PMPSE. As per the prescribed format, network operators were also asked about other network operators about whom they may be aware of. New network operators identified through this process were also contacted to collect information by repeating the same process.

### Mapping and size estimation in LWS villages

The PMPSE estimated the size of KPs in LWS villages. All the villages covered by LWS in three years preceding the PMPSE were included. Data collection was conducted using standardised Village Information Format (VIF) (S3 Appendix).

### Field quality assurance

PMPSE was implemented across the country using an operational manual which was critical for ensuring standardized and quality implementation by providing technical protocol, methodological instructions, roles and responsibilities along with troubleshooting guidelines. This operational manual was used to create a pool of master trainers in each State and Union Territory. These master trainers were selected from the State AIDS Control Societies (SACS) and Technical Support Units (TSU). They trained project managers, counsellors, and monitoring and evaluation (M&E) personnel working in targeted interventions and Link Worker Schemes (LWS). Subsequently, these trained individuals trained outreach workers, peer educators, and cluster link workers in their respective districts. The national team and master trainers from SACS and TSUs were also responsible for monitoring the progress and conducting spot checks in the field. Supervisory visits included back check at 10% of the hotpots.

### Data management and analysis

Field data collection was done using paper forms. A web-based portal with inbuilt quality checks was developed to enable data entry of the of paper forms. Data were entered in the portal by M&E personnel of TIs and LWS. The data entered in the portal was exported and then analysed in Microsoft Excel to generate estimates after making required adjustments for overlaps.

District was the unit of analysis for generating the size estimates after adjusting for the overlaps. The size estimate for a district was achieved by undertaking analysis of data recorded in HIF, VIF and NOF in a sequential manner.

#### Duplication adjustment for KP at hotspots in a district.

First, size estimate was undertaken for the physical hotspots. KP size estimate at each of the hotspot was calculated after adjusting for the multiplicity in a hotspot visited by a KP. At each of the hotspots, the data collection tool collected information from the key informants about number of key populations who may also be visiting other hotspots. Information provided on this question was used to adjust for duplication on account of mobility between the venues. The adjusted size estimate of KPs at all hotspots in a district (AHS_d_) was calculated by summing up the adjusted size estimates at each hotspot within that district. To obtain the adjusted size estimate at each hotspot, we subtracted the number of KPs visiting multiple hotspots from the total unadjusted number of KPs (UVS_i_) associated with that hotspot. By summing up these adjusted size estimates across all hotspots within a district, we obtain the adjusted size estimate for that district (AHS_d_). Following mathematical formula was used to generate the adjusted estimate for the district.


AHSd=∑UHSi−1/M(Wi*UHSi)

Where,

𝑈𝐻𝑆_𝑖_ = Unadjusted size of KPs at a physical hotspot,

W_i_ = Weighted proportion of KPs operating at multiple sites, derived by summation of multiplication of average number of KPs at each hotspot with average proportion going out at each hotspot in a given district divided by summation of average number of KPs at each hotspot in a given district. Weighted proportion was derived for each district separately based on the information collected in all hotspot information format. An illustration is also provided as supplementary information (S4 Appendix).

M = Average number of hotspots visited by a KP, and

AHS_d_ = Total estimated size (adjusted) of KPs at all of the physical hotspot of the district on a typical day.

#### Duplication adjustment for KPs with network operators in a district.

Then, we estimated the size of the key population exclusively associated with the network operators. The data collection tool for network operators collected information about size of key populations associated with them as well as other network operators. It also collected information on the size of key populations associated with them who might also be visiting physical hotspots. For network operators, duplicate adjustment was thus done by first accounting for key populations’ associations with multiple operators and also for their visits to physical venues. This resulted in a size estimate of key populations exclusively associated with network operators.

Mathematically, we employed a two-step adjustment process to estimate the size of KPs exclusively associated with network operators in a given district. First, we calculated the total adjusted size of KPs associated with all of network operators in a district (N1S_d_) by summing up the adjusted size estimates with each network operator in that district. We calculated the adjusted size with each network operator by subtracting the number of KPs associated with multiple network operators from the total unadjusted number of KPs associated with that network operator (UN1S_i_).

After obtaining the value for N1S_d_, we made further adjustment to account for KPs with network operators who are also associated with hotspots. We applied a weighted proportion (W_pi_) to the adjusted size (N1S_d_) to estimate the size of KPs also visiting physical hotspots. By subtracting the estimated size of KPs visiting physical hotspots from total number of KPs associated with network operators in a district (N1S_d),_ we derived the total number of KPs associated exclusively with network operators in a district (𝐴𝑁𝑆_d_). Following mathematical formula was used to generate the size estimates of KPs exclusively available with network operators.


N1Sd=∑UN1Si−1/M(Wi*UN1Si)

and


ANSd=∑N1Si−(Wpi*N1Si)

Where,

UN1S_i_ = Unadjusted size of KPs with a network operator,

W_*i*_ = Weighted proportion of KPs associated with multiple operators derived by summation of multiplication of number of KP with each network operator with proportion of KP associated with other network operators divided by summation of number of KP with each network operator. Weighted proportion was derived for each district separately based on the information collected in all network information format in a district.

M = Average number of network operators with whom a KP is associated,

N1*S*_d_ = Total size (adjusted) of KP with a network operator in a given district,

W_pi_ = Weighted proportion of KP who are also associated with hotspots, and

𝐴𝑁𝑆_d_ = Total estimated size (adjusted) of KPs exclusively with network operator in a given district.

#### Duplication adjustment for KPs in link worker villages in a district.

Next, we estimated the exclusive size of key population in the link worker villages. In the village information format, information was collected on the total number of KPs’ in that village as well as also about number among them who may also be visiting physical hotspots. The estimation of KPs exclusively in link worker villages was done by adjusting for KPs who also visited hotspots for high-risk behaviours using the mathematical formula mentioned below.


AVSd=∑UVSi−(Wi *UVSi)

Where,

𝑈𝑉𝑆_𝑖_  = Unadjusted size of KPs for the village,

W_i_ = Weighted proportion of KPs visiting hotspots in urban areas, and

𝐴𝑉𝑆_d_ = Total estimated size (adjusted) of KPs exclusively in LWS villages in a given district.

#### Final adjusted size estimates for a district.

In the final step, we worked out the total estimated size in a district by summing the estimated size (adjusted) at all the physical hotspots of the district (𝐴𝑉𝑆_d_), estimated size (adjusted) of KPs exclusively with network operator in district (𝐴𝑉𝑆_d_), and estimated size (adjusted) of KPs exclusively in LWS villages of the district (𝐴𝑉𝑆_d_).

### Ethical considerations

Given the sensitive nature of the locations and populations under consideration, PMPSE employed all necessary measures to ensure the appropriate protection of respondents. Administration of participants information sheet (PIS) and written informed consent from respondents aged 18 or older in local language was central to PMPSE implementation (S5 Appendix). The PIS specified that any information given by respondents would remain confidential and only be shared with the programme team. Respondents agreed through the ICF to have their anonymized and aggregated data published or shared. For illiterate respondents, informed consent through thumb impression was taken in the presence of an impartial literate witness. Sensitisation and training of the field teams, and community participation across PMPSE implementation through CABs were other key approaches for getting support from the KPs as well as strengthening of respondent protection measures.

Hard copies of the data forms were kept under lock and keys by the implementation partners. Data collected in hard copies by the team were entered into the secure web-based portal. The digitalized data providing the characteristics of the hotspots and network operators including location, estimated size, operational days, time, etc. were made available in soft copy only to the TI programme, TSU, SACS and NACO for programmatic purposes. Ethical approval for PMPSE was obtained from the Ethics Committee of National AIDS Control Organisation, Ministry of Health and Family Welfare, Government of India (T-11020/48/2019-NACO-R&D dated 12.09.2019).

## Results

Tables 2–9 presents the State/UT-wise KP size estimates for FSWs, MSM, PWIDs, and H/TG people. Overall, PMPSE estimated 9,95,499 (9,02,277–10,88,712) FSWs, 3,51,020 (3,13,860–3,88,175) MSM, 2,88,715 (2,53,025-3,24,408) PWIDs, and 96,193 (85,206-1,07,174) H/TG people. This works out to 2.75 (2.49 - 3.00) FSW per 1000 adult women, 0.91 (0.81 – 1.01) MSM, 0.75 (0.66 - 0.84) PWIDs and 0.25 (0.22 – 0.28) H/TG people per 1000 adult men.

**Table 2:** FSW size estimates, PMPSE 2020–22.

State/UT	Total hotspots	Total network operators	LWS villages	Size estimates	Average number of FSW			Percentage distribution of estimated size			Number of FSWs per 1000 adult women
					Per hotspot	Per network operator	Per LWS village	At hotspots	Exclusively with network operators	Exclusively in LWS villages	
Andhra Pradesh	3,593	148	1598	119367 (102886 -135847)	29	6	10	85.88	0.72	13.40	8.08 (6.97 - 9.2)
Arunachal Pradesh	570	0	0	6940 (6263 -7617)	12	–	–	100.00	0.00	0.00	17.24 (15.56 - 18.93)
Assam	2,034	182	0	39720 (34891 -44550)	19	6	–	97.26	2.74	0.00	4.07 (3.58 - 4.57)
Bihar	516	39	400	11681 (11019 -12343)	18	8	5	79.21	2.74	18.05	0.38 (0.36 - 0.4)
Chandigarh	131	44		3333 (2997 -3668)	23	6	–	91.54	8.46	0.00	10.1 (9.08 - 11.12)
Chhattisgarh	929	68	266	18375 (16858 -19891)	18	6	4	92.56	2.30	5.14	2.31 (2.12 - 2.5)
Delhi	291	2845	0	88399 (87811 -88987)	26	28	–	8.53	91.47	0.00	15.45 (15.35 - 15.55)
Goa	248	5	0	5040 (4304 -5776)	20	1	–	99.92	0.08	0.00	11.66 (9.96 - 13.37)
Gujarat	1,580	81	1075	37118 (33444 -40791)	19	20	5	80.82	4.29	14.89	2.04 (1.84 - 2.24)
Haryana	1,326	221	0	17667 (16083 -19251)	12	7	–	91.53	8.47	0.00	2.27 (2.07 - 2.48)
Himachal Pradesh	913	27	0	13210 (11887 -14533)	14	5	–	99.03	0.97	0.00	6.59 (5.93 - 7.25)
Jammu And Kashmir	303	52	0	4633 (3898 -5368)	14	8	–	91.48	8.52	0.00	1.26 (1.06 - 1.46)
Jharkhand	846	0	0	11860 (10567 -13152)	14	–	–	100.00	0.00	0.00	1.16 (1.03 - 1.28)
Karnataka	8,771	2718	1179	153336 (140575 -166098)	13	7	16	74.46	13.03	12.52	8.34 (7.64 - 9.03)
Kerala	1,519	107	0	16623 (14316 -18922)	11	5	–	96.58	3.42	0.00	1.8 (1.55 - 2.05)
Madhya Pradesh	2,841	617	1146	53454 (47189 -59720)	16	8	2	86.15	8.70	5.14	2.45 (2.16 - 2.73)
Maharashtra	3,273	1291	2388	95351 (88080 -102622)	23	8	4	79.72	11.16	9.12	2.85 (2.63 - 3.07)
Manipur	341	32	696	5661 (4484 -6839)	16	1	0	94.37	0.70	4.93	6.45 (5.11 - 7.8)
Meghalaya	116	10	46	3296 (2756 -3837)	26	6	6	89.87	1.85	8.28	3.96 (3.31 - 4.61)
Mizoram	116	0	197	1433 (1255 -1611)	9	–	2	74.26	0.00	25.74	4.37 (3.83 - 4.91)
Nagaland	211	18	0	2245 (1765 -2725)	10	6	–	95.25	4.75	0.00	3.83 (3.01 - 4.65)
Odisha	2,113	190	957	24620 (21528 -27712)	10	5	2	88.63	3.98	7.39	1.97 (1.72 - 2.21)
Puducherry	101	13	0	2514 (2196 -2831)	24	7	–	96.54	3.46	0.00	5.35 (4.68 - 6.03)
Punjab	1,296	285	596	27303 (24301 -30306)	18	7	4	83.12	7.81	9.06	3.39 (3.02 - 3.77)
Rajasthan	1,155	233	0	21033 (19196 -22870)	17	8	–	91.07	8.93	0.00	1.01 (0.92 - 1.1)
Sikkim	75	0	0	731 (634 -828)	10	–	–	100.00	0.00	0.00	3.9 (3.38 - 4.42)
Tamil Nadu	2,516	372	2042	60775 (54633 -66917)	19	8	5	78.08	5.09	16.84	2.91 (2.61 - 3.2)
Telangana	1,624	568	1264	75380 (68300 -82461)	38	6	8	82.12	4.50	13.39	6.99 (6.33 - 7.64)
Tripura	691	38	271	6242 (5750 -6734)	8	4	2	87.95	2.55	9.50	5.52 (5.09 - 5.96)
Uttar Pradesh	2,412	384	1171	40479 (37074 -43885)	15	8	2	87.24	7.46	5.30	0.67 (0.61 - 0.73)
Uttarakhand	421	62	0	7213 (6386 -8040)	17	2	–	98.35	1.65	0.00	2.28 (2.01 - 2.54)
West Bengal	707	68	803	20452 (18936 -21967)	26	9	2	88.82	2.88	8.30	0.74 (0.69 - 0.8)
India	43,579	10718	16095	995499 (902277 -1088712)	18	13	5	77.49	13.96	8.56	2.74 (2.49 - 3)

**Table 3:** State/UT-wise distribution of districts by FSW size estimates categories from PMPSE 2020–22.

State/UT	Total Districts covered	>=5000	2500- < 5000	1000- < 2500	500- < 1000	<500
Andhra Pradesh	22	5	10	4	2	–
Arunachal Pradesh	16	–	–	1	5	10
Assam	26	–	2	15	7	2
Bihar	18	–	–	3	8	7
Chandigarh	1	–	1	–	–	–
Chhattisgarh	23	–	1	5	9	8
Delhi	11	9	1	1	–	–
Goa	2	–	1	1	–	–
Gujarat	33	2	–	8	11	11
Haryana	20	–	–	7	10	3
Himachal Pradesh	10	–	–	8	2	–
Jammu And Kashmir	19	–	–	–	3	15
Jharkhand	20	–	–	1	11	8
Karnataka	30	7	14	7	1	1
Kerala	14	–	1	6	7	–
Madhya Pradesh	50	–	2	20	16	12
Maharashtra	32	4	4	16	6	2
Manipur	14	–	–	–	7	7
Meghalaya	8	–	–	–	2	5
Mizoram	8	–	–	–	1	7
Nagaland	12	–	–	–	2	7
Odisha	30	–	–	10	11	9
Puducherry	4	–	–	1	2	1
Punjab	21	–	2	11	6	2
Rajasthan	31	–	–	6	14	11
Sikkim	2	–	–	–	–	2
Tamil Nadu	31	–	9	16	4	2
Telangana	33	6	3	1	3	18
Tripura	8	–	–	1	4	3
Uttar Pradesh	70	–	1	8	21	40
Uttarakhand	11	–	–	2	3	6
West Bengal	21	1	–	3	9	8
**India**	**651**	**34**	**52**	**162**	**187**	**207**

**Table 4:** MSM size estimates, PMPSE 2020–22.

State/UT	Total hotspots	Total network operators	LWS villages	Size estimates	Average number of MSM			Percentage distribution of estimated size			Number of MSM per 1000 adult men
					Per hotspot	Per network operator	Per LWS village	At hotspots	Exclusively with network operators	Exclusively in LWS villages	
Andhra Pradesh	856	39	370	22059 (18564 -25554)	25	8	2	95.58	1.39	3.03	1.48 (1.24 - 1.71)
Arunachal Pradesh	125	0	0	900 (810 -990)	7	–	–	100.00	0.00	0.00	2.1 (1.89 - 2.32)
Assam	858	89	0	16666 (14544 -18788)	19	6	–	96.76	3.24	0.00	1.68 (1.46 - 1.89)
Bihar	101	15	215	2376 (2236 -2516)	18	10	2	78.15	6.11	15.74	0.07 (0.06 - 0.07)
Chandigarh	106	16	0	2569 (2306 -2832)	23	9	–	94.51	5.49	0.00	6.46 (5.8 - 7.13)
Chhattisgarh	189	8	99	2888 (2629 -3148)	15	0	1	97.40	0.00	2.60	0.35 (0.32 - 0.39)
Delhi	851	110	0	27026 (24984 -29067)	29	23	–	90.44	9.56	0.00	4.15 (3.84 - 4.47)
Goa	122	2	0	3338 (2786 -3891)	27	4	–	99.77	0.23	0.00	7.35 (6.13 - 8.56)
Gujarat	1,505	0	995	34299 (30660 -37937)	20	–	4	87.43	0.00	12.57	1.67 (1.5 - 1.85)
Haryana	738	42	0	8022 (7205 -8838)	11	6	–	96.76	3.24	0.00	0.9 (0.81 - 0.99)
Himachal Pradesh	153	4	0	1251 (1080 -1422)	8	2	–	99.22	0.78	0.00	0.59 (0.51 - 0.67)
Jammu And Kashmir	59	10	0	676 (547 -805)	10	9	–	86.69	13.31	0.00	0.16 (0.13 - 0.19)
Jharkhand	157	0	0	1742 (1493 -1990)	11	–	–	100.00	0.00	0.00	0.16 (0.13 - 0.18)
Karnataka	2,810	198	583	45630 (41397 -49864)	15	10	2	93.46	4.22	2.32	2.38 (2.16 - 2.6)
Kerala	863	55	0	13836 (11647 -16021)	16	3	–	98.68	1.32	0.00	1.59 (1.34 - 1.84)
Madhya Pradesh	1,188	161	109	18084 (15782 -20387)	14	7	1	93.19	6.26	0.55	0.76 (0.66 - 0.86)
Maharashtra	1,272	383	469	40186 (37260 -43113)	24	23	1	76.76	21.74	1.50	1.08 (1 - 1.16)
Manipur	107	0	213	1506 (1258 -1755)	13	–	0	93.50	0.00	6.50	1.74 (1.45 - 2.02)
Meghalaya	16	4	1	343 (251 -435)	20	5	1	93.46	6.25	0.29	0.41 (0.3 - 0.52)
Mizoram	83	0	55	841 (717 -964)	9	–	2	87.87	0.00	12.13	2.53 (2.15 - 2.9)
Nagaland	102	1	0	1238 (1051 -1425)	9	370	–	70.13	29.87	0.00	2.01 (1.7 - 2.31)
Odisha	618	24	458	5977 (5260 -6693)	9	4	1	88.42	1.69	9.89	0.47 (0.42 - 0.53)
Puducherry	98	4	0	2488 (2179 -2798)	24	45	–	92.78	7.22	0.00	5.79 (5.07 - 6.51)
Punjab	542	63	238	8044 (7015 -9074)	14	6	1	92.37	5.07	2.56	0.88 (0.76 - 0.99)
Rajasthan	575	63	0	7350 (6511 -8190)	12	6	–	94.53	5.47	0.00	0.33 (0.29 - 0.37)
Sikkim	0	0	0	–	–	–	–	–	–	–	–
Tamil Nadu	1,629	148	1113	38283 (33927 -42640)	20	22	1	87.14	8.55	4.30	1.84 (1.63 - 2.04)
Telangana	292	76	323	16427 (15281 -17572)	50	16	2	89.51	7.54	2.95	1.5 (1.39 - 1.6)
Tripura	126	4	61	875 (808 -943)	6	8	1	90.92	3.71	5.37	0.74 (0.69 - 0.8)
Uttar Pradesh	1,468	97	216	19668 (17776 -21560)	13	12	0	93.47	6.01	0.52	0.3 (0.27 - 0.33)
Uttarakhand	188	13	0	2882 (2647 -3118)	15	1	–	99.73	0.27	0.00	0.86 (0.79 - 0.94)
West Bengal	170	31	69	3532 (3234 -3829)	18	15	1	85.02	12.86	2.12	0.12 (0.11 - 0.13)
India	17,967	1,660	5,587	351020 (313860 -388175)	18	14	2	90.23	6.76	3.00	0.91 (0.81 - 1)

**Table 5:** State/UT-wise distribution of districts by MSM size estimates categories from PMPSE 2020–22.

State/UT	Total Districts covered	>=5000	2500- < 5000	1000- < 2500	500- < 1000	<500
Andhra Pradesh	22	1	2	1	7	10
Arunachal Pradesh	16	–	–	–	1	4
Assam	26	–	2	3	2	15
Bihar	18	–	–	–	1	16
Chandigarh	1	–	1	–	–	–
Chhattisgarh	23	–	–	–	1	16
Delhi	11	–	5	6	–	–
Goa	2	–	–	2	–	–
Gujarat	33	2	1	6	9	14
Haryana	20	–	–	1	5	13
Himachal Pradesh	10	–	–	–	–	10
Jammu And Kashmir	19	–	–	–	–	8
Jharkhand	20	–	–	–	1	6
Karnataka	30	–	5	12	9	4
Kerala	14	–	–	8	4	1
Madhya Pradesh	50	–	–	6	7	34
Maharashtra	32	2	2	7	6	15
Manipur	14	–	–	–	–	10
Meghalaya	8	–	–	–	–	2
Mizoram	8	–	–	–	1	4
Nagaland	12	–	–	–	1	3
Odisha	30	–	–	–	2	26
Puducherry	4	–	–	1	–	3
Punjab	21	–	–	1	3	17
Rajasthan	31	–	–	–	1	30
Sikkim	2	–	–	–	–	–
Tamil Nadu	31	1	1	13	7	9
Telangana	33	1	1	3	2	19
Tripura	8	–	–	–	–	8
Uttar Pradesh	70	–	–	2	7	57
Uttarakhand	11	–	–	–	3	7
West Bengal	21	–	–	–	1	17
**India**	**651**	**7**	**20**	**72**	**81**	**378**

**Table 6:** PWID size estimates, PMPSE 2020–22.

State/UT	Total hotspots	Total network operators	LWS villages	Size estimates	Average number of PWID			Percentage distribution of estimated size			Number of PWID per 1000 adult men
					Per hotspot	Per network operator	Per LWS village	At hotspots	Exclusively with network operators	Exclusively in LWS villages	
Andhra Pradesh	147	1	0	1332 (1059 -1604)	9	1	–	97.00	3.00	0.00	0.08 (0.07 - 0.1)
Arunachal Pradesh	407	0	0	5142 (4676 -5608)	13	–	–	100.00	0.00	0.00	12.04 (10.95 - 13.13)
Assam	1,145	105	0	26155 (22963 -29347)	22	1	–	96.70	3.30	0.00	2.63 (2.31 - 2.96)
Bihar	215	21	120	4585 (4249 -4920)	20	1	1	92.35	4.05	3.60	0.13 (0.12 - 0.14)
Chandigarh	82	6	0	1908 (1701 -2115)	23	1	–	98.06	1.94	0.00	4.8 (4.28 - 5.32)
Chhattisgarh	222	22	15	3922 (3535 -4309)	17	1	0.4	94.78	5.06	0.15	0.48 (0.43 - 0.53)
Delhi	1,249	22	0	32481 (29446 -35515)	25	1	–	97.73	2.27	0.00	4.99 (4.52 - 5.46)
Goa	22	0	0	301 (246 -356)	14	–	–	100.00	0.00	0.00	0.66 (0.54 - 0.78)
Gujarat	83	0	0	778 (637 -920)	9	–	–	100.00	0.00	0.00	0.03 (0.03 - 0.04)
Haryana	1,535	73	0	19024 (17192 -20856)	12	1	–	96.78	3.22	0.00	2.14 (1.93 - 2.34)
Himachal Pradesh	346	4	0	3661 (3213 -4109)	11	1	–	99.67	0.33	0.00	1.74 (1.52 - 1.95)
Jammu And Kashmir	617	133	0	10162 (8022 -12301)	14	1	–	86.57	13.43	0.00	2.49 (1.96 - 3.01)
Jharkhand	61	0	0	778 (641 -916)	13	–	–	100.00	0.00	0.00	0.07 (0.06 - 0.08)
Karnataka	479	12	1	4330 (3770 -4890)	8	1	0.0	92.71	7.29	0.00	0.22 (0.19 - 0.25)
Kerala	370	16	0	3276 (2628 -3922)	8	1	–	92.10	7.88	0.00	0.37 (0.3 - 0.45)
Madhya Pradesh	784	102	3	11918 (10445 -13392)	14	1	3	90.68	9.25	0.07	0.5 (0.44 - 0.56)
Maharashtra	64	4	2	1096 (910 -1282)	17	1	4	97.83	1.55	0.64	0.02 (0.02 - 0.03)
Manipur	1,409	14	846	24984 (20446 -29522)	16	1	2	92.85	0.43	6.72	28.86 (23.62 - 34.11)
Meghalaya	142	0	29	3174 (2715 -3634)	21	–	7	93.29	0.00	6.71	3.83 (3.28 - 4.39)
Mizoram	653	0	172	10397 (8927 -11866)	15	–	3	94.94	0.00	5.06	31.29 (26.87 - 35.72)
Nagaland	1,194	36	0	16802 (14136 -19467)	14	1	–	97.89	2.11	0.00	27.32 (22.99 - 31.66)
Odisha	401	46	9	4525 (3918 -5133)	10	1	1	90.46	9.40	0.13	0.36 (0.31 - 0.41)
Puducherry	8	0	0	20 (13 -28)	3	–	–	100.00	0.00	0.00	0.04 (0.03 - 0.06)
Punjab	1,915	162	742	45098 (40325 -49871)	18	1	11	77.71	4.54	17.75	4.94 (4.42 - 5.47)
Rajasthan	224	26	0	3023 (2711 -3336)	13	1	–	93.90	6.10	0.00	0.13 (0.12 - 0.15)
Sikkim	58	0	0	821 (713 -929)	14	–	–	100.00	0.00	0.00	3.87 (3.36 - 4.38)
Tamil Nadu	14	0	1	115 (91 -138)	8	–	1	99.13	0.00	0.87	0 (0 - 0)
Telangana	64	0	1	815 (656 -974)	13	–	0	100.00	0.00	0.00	0.07 (0.06 - 0.08)
Tripura	653	34	144	6499 (5952 -7047)	9	1	2	90.55	4.27	5.18	5.55 (5.09 - 6.02)
Uttar Pradesh	2,392	87	211	35412 (31669 -39155)	14	1	1	97.07	2.57	0.36	0.54 (0.48 - 0.59)
Uttarakhand	213	10	0	3835 (3406 -4263)	18	1	–	100.00	0.00	0.00	1.15 (1.02 - 1.28)
West Bengal	120	17	53	2333 (1997 -2670)	17	1	2	89.24	5.27	5.48	0.08 (0.07 - 0.09)
India	17,288	953	2,349	288717 (253024 -324407)	15	1	5	92.59	3.53	3.88	0.75 (0.65 - 0.84)

**Table 7:** State/UT-wise distribution of districts by PWID size estimates categories from PMPSE 2020–22.

State/UT	Total Districts covered	>=5000	2500- < 5000	1000- < 2500	500- < 1000	<500
Andhra Pradesh	22	–	–	–	1	2
Arunachal Pradesh	16	–	–	1	3	12
Assam	26	1	1	6	8	7
Bihar	18	–	–	1	1	15
Chandigarh	1	–	–	1	–	–
Chhattisgarh	23	–	–	1	3	6
Delhi	11	–	7	3	1	–
Goa	2	–	–	–	–	2
Gujarat	33	–	–	–	–	3
Haryana	20	–	2	4	6	8
Himachal Pradesh	10	–	–	–	3	7
Jammu And Kashmir	19	–	–	2	6	11
Jharkhand	20	–	–	–	–	4
Karnataka	30	–	1	1	–	1
Kerala	14	–	–	1	2	4
Madhya Pradesh	50	–	–	3	3	25
Maharashtra	32	–	–	–	1	4
Manipur	16	1	2	5	4	3
Meghalaya	8	–	–	1	1	5
Mizoram	8	–	1	3	3	1
Nagaland	12	–	3	4	4	1
Odisha	30	–	–	1	4	11
Puducherry	4	–	–	–	–	1
Punjab	21	2	4	9	4	2
Rajasthan	31	–	–	–	1	26
Sikkim	2	–	–	–	1	1
Tamil Nadu	31	–	–	–	–	3
Telangana	33	–	–	–	1	–
Tripura	8	–	–	2	5	1
Uttar Pradesh	70	–	1	8	12	47
Uttarakhand	11	–	–	1	3	4
West Bengal	19	–	–	1		7
India	651	4	22	59	81	224

**Table 8:** H/TG people size estimates, PMPSE 2020–22.

State/UT	Total hotspots	Total network operators	LWS villages	Size estimates	Average number of H/TG people			Percentage distribution of estimated size			Number of H/TG people per 1000 adult men
					Per hotspot	Per network operator	Per LWS village	At hotspots	Exclusively with network operators	Exclusively in LWS villages	
Andhra Pradesh	265	9	53	5363 (4418 -6308)	20	7	1	97.94	1.15	0.91	0.36 (0.29 - 0.42)
Arunachal Pradesh	51	0	0	139 (111 -168)	3			100.00	0.00	0.00	0.32 (0.26 - 0.39)
Assam	197	43	0	2481 (2185 -2776)	11	5		90.53	9.47	0.00	0.25 (0.22 - 0.28)
Bihar	58	12	2	846 (774 -917)	13	7	1	89.31	10.57	0.12	0.02 (0.02 - 0.02)
Chandigarh	15	1	0	164 (142 -185)	10	9		94.31	5.49	0.00	0.41 (0.35 - 0.46)
Chhattisgarh	102	5	33	1118 (990 -1246)	11	2	0.8	96.75	0.83	2.41	0.13 (0.12 - 0.15)
Delhi	667	56	0	17906 (16187 -19626)	24	31		90.28	9.72	0.00	2.75 (2.49 - 3.01)
Goa	9	2	0	131 (115 -147)	13	6		90.23	9.77	0.00	0.28 (0.25 - 0.32)
Gujarat	117	0	134	2604 (2395 -2813)	21		1	93.51	0.00	6.49	0.12 (0.11 - 0.13)
Haryana	151	6	0	1434 (1275 -1594)	9	4		98.26	1.74	0.00	0.16 (0.14 - 0.17)
Himachal Pradesh	33	1	0	257 (222 -293)	8	7		97.45	2.55	0.00	0.12 (0.1 - 0.13)
Jammu And Kashmir	46	7	0	613 (530 -696)	12	9		89.77	10.23	0.00	0.15 (0.13 - 0.17)
Jharkhand	43	0	0	482 (420 -544)	11			100.00	0.00	0.00	0.04 (0.03 - 0.05)
Karnataka	860	115	23	10926 (9836 -12016)	12	8	0.7	91.68	8.18	0.14	0.57 (0.51 - 0.62)
Kerala	273	1	0	2604 (2167 -3036)	10	7		99.73	0.27	0.00	0.29 (0.24 - 0.34)
Madhya Pradesh	129	38	5	1613 (1423 -1803)	9	11	1	73.29	26.33	0.37	0.06 (0.06 - 0.07)
Maharashtra	477	46	183	10323 (9250 -11396)	20	15	1	90.67	6.76	2.57	0.27 (0.25 - 0.3)
Manipur	59	0	0	468 (394 -541)	8			100.00	0.00	0.00	0.54 (0.45 - 0.62)
Meghalaya	8	5	1	109 (84 -135)	8	8	1	60.64	38.45	0.91	0.13 (0.1 - 0.16)
Mizoram	0	0	0	0 (0 -0)							0 (0 - 0)
Nagaland	13	0	0	82 (61 -102)	6			100.00	0.00	0.00	0.13 (0.1 - 0.16)
Odisha	717	91	175	7209 (6277 -8140)	9	5	1	90.86	6.37	2.77	0.57 (0.5 - 0.65)
Puducherry	21	0	0	203 (176 -229)	10			100.00	0.00	0.00	0.47 (0.41 - 0.53)
Punjab	90	8	11	1315 (1167 -1464)	14	11	0	93.33	6.44	0.23	0.14 (0.12 - 0.16)
Rajasthan	203	51	0	2125 (1882 -2369)	9	5		87.23	12.77	0.00	0.09 (0.08 - 0.1)
Sikkim	0	0	0	0 (0 -0)							0 (0 - 0)
Tamil Nadu	677	59	134	9210 (7734 -10687)	12	14	1	89.97	8.97	1.05	0.44 (0.37 - 0.51)
Telangana	32	18	19	995 (918 -1071)	25	11	1	79.09	19.42	1.51	0.09 (0.08 - 0.09)
Tripura	9	6	0	158 (153 -163)	5	20		25.92	74.08	0.00	0.13 (0.13 - 0.14)
Uttar Pradesh	944	105	15	9846 (8880 -10812)	9	9	1	90.76	9.08	0.15	0.15 (0.13 - 0.16)
Uttarakhand	33	2	0	321 (293 -348)	10	1		99.38	0.62	0.00	0.09 (0.08 - 0.1)
West Bengal	286	33	98	5134 (4730 -5537)	15	18	2	84.86	11.68	3.47	0.18 (0.16 - 0.19)
India	6,585	720	886	96193 (85206 -107174)	13	11	1	90.84	8.07	1.08	0.25 (0.22 - 0.28)

**Table 9:** State/UT-wise distribution of districts by H/TG people size estimates categories from PMPSE 2020–22.

State/UT	Total Districts covered	>=5000	2500- < 5000	1000- < 2500	500- < 1000	<500
Andhra Pradesh	22	–	–	1	3	16
Arunachal Pradesh	16	–	–	–	–	3
Assam	26	–	–	1	–	15
Bihar	18	–	–	–	–	18
Chandigarh	1	–	–	–	–	1
Chhattisgarh	23	–	–	–	–	16
Delhi	11	–	1	8	1	1
Goa	2	–	–	–	–	2
Gujarat	33	–	–	1	–	22
Haryana	20	–	–	–	1	14
Himachal Pradesh	10	–	–	–	–	10
Jammu And Kashmir	19	–	–	–	–	9
Jharkhand	20	–	–	–	–	8
Karnataka	30	–	1	–	4	23
Kerala	14	–	–	–	1	8
Madhya Pradesh	50	–	–	–	–	30
Maharashtra	32	–	1	1	2	17
Manipur	14	–	–	–	–	2
Meghalaya	8	–	–	–	–	3
Mizoram	8	–	–	–	–	
Nagaland	12	–	–	–	–	2
Odisha	30	–	–	1	2	27
Puducherry	4	–	–	–	–	3
Punjab	21	–	–	–	1	15
Rajasthan	31	–	–	–	1	29
Sikkim	2	–	–	–	–	
Tamil Nadu	31	–	–	1	2	27
Telangana	33	–	–	–	–	10
Tripura	8	–	–	–	–	5
Uttar Pradesh	70	–	–	–	4	66
Uttarakhand	11	–	–	–	–	7
West Bengal	21	–	–	–	2	18
Total	651	–	3	14	24	427

### Female sex workers

PMPSE for FSWs identified 43,579 hotspots and 10,718 network operators. FSWs were further reported in a total of 16,095 villages. Slightly more than half (55.1%) of the hotspots were home-based, followed by 16.1% street-based locations and 5.9% brothel-based. A total of 1,34,677 key informants were interviewed, including 98,923 from key population groups.

Out of total estimated 9,95,499 (9,02,277–10,88,712) FSWs, around 77.0% were at hotspots (around 8 FSW per hotspot), 14.0% were exclusively with network operators (around 13 FSW per network operator) and 9.0% (S1 Table) were exclusively in link worker villages (around 5 FSW per LWS village).

Karnataka (15.4%), Andhra Pradesh (12.0%), Maharashtra (9.6%), Delhi (8.9%) and Telangana (7.6%) were States with highest size of FSW contributing around 53.0% of total FSW KP size in the country. Number of FSW per 1000 adult women was highest in Arunachal Pradesh (17.24), followed by that in Delhi (15.46), Goa (11.67), Chandigarh (10.10) and Karnataka (8.34).

Highest number of hotspots in PMPSE were mapped in Karnataka (around 20.0% of the total hotspots), followed by that in Andhra Pradesh and Maharashtra (around 8.0% each), Madhya Pradesh (around 7.0%) and Tamil Nadu (around 6.0%). In Telangana, there were around 38 FSWs per hotspot, highest in the country followed by 29 FSWs per hotspot in Andhra Pradesh and 26 FSWs per hotspot in Delhi, Meghalaya and West Bengal.

Almost two thirds of the network operators were mapped in three States, i.e., Delhi (27.0%), Karnataka (25.0%) and Maharashtra (12.0%). PMPSE mapped at least 100 network operators in State of Andhra Pradesh (148), Assam (182), Haryana (221), Kerala (107), Madhya Pradesh (617), Odisha (190), Punjab (285), Rajasthan (233), Tamil Nadu (372), Telangana (568), and Uttar Pradesh (384).

In Karnataka, there were 16 FSWs per LWS village operating only in the given village followed by 10 in Andhra Pradesh and 8 in Telangana. Bihar, Gujarat, Meghalaya and Tamil Nadu were the other States where 5–6 FSWs, on an average, operated exclusively in LWS village.

The PMPSE reported presence of FSWs in 642 districts of the country (S2 Table). In 34 districts (5-Andhra Pradesh, 9-Delhi, 2-Gujarat, 7-Karnataka, 4-Maharasthra, 6-Telangama, and 1-West Bengal) PMPSE reported at least 5000 female sex workers. FSWs in range of 2500 to < 5000 was reported in 52 districts (10-Andhra Pradesh, 2-Assam, 1-Chandigarh, 1-Chhatisgarh, 1-Delhi, 1-Goa, 14-Karnataka, 1-Kerala, 2-Madhya Pradesh, 4-Maharasthra, 2-Punjab, 9-Tamil Nadu, 3-Telangama, and 1-Uttar Pradesh). PMPSE reported FSWs in range of 1000 to < 2500 in 162 districts, in range of 500 to < 1000 in 187 districts and less than 500 in 207 districts (Table 3). Broadly, districts in southern and western regions have much higher size estimates of FSW vis-à-vis rest of the country.

### Men who have sex with men

PMPSE for MSM identified 17,967 hotspots and 1,660 network operators. MSM were further reported in a total of 5,587 villages. Nationally, 14.5% of the MSM hotspots were street-based, while another 14.1% were situated near bus stands, followed by 11.1% which were home-based. Further, around 18.0% of the hotspots were located in parks or marketplaces, 9.4% were identified in old or vacant buildings or abandoned/dilapidated structures, and 5.0% were in proximity to railway stations. A total of 55,922 key informants were interviewed, including 40,983 from key population groups.

Out of the total estimated 3,51,020 (3,13,860–3,88,175) MSM, around 90.2% were at hotspots (around 18 MSM per hotspot), 6.8% were exclusively with network operators (around 14 MSM per network operator) and 3.0% (S3 Table) were exclusively in link worker villages (around 2 MSM per LWS village).

Karnataka (15.4%), Maharashtra (11.4%), Tamil Nadu (10.9%), Gujarat (9.8%), Delhi (7.7%) and Andhra Pradesh (6.3%) were the States with the highest size of MSM contributing around 60% of the total size in the country. The number of MSM per 1000 adult men was highest in Goa (7.35), followed by Chandigarh (6.46), Puducherry (5.79) and Delhi (4.15).

Highest number of hotspots in PMPSE were mapped in Karnataka (around 15.6% of the total hotspots), followed by Tamil Nadu (9.1%), Uttar Pradesh and Gujarat (around 8.0% each). In Telangana, there were around 50 MSM per hotspot, the highest in the country, followed by 29 MSM per hotspot in Delhi, 27 MSM per hotspot in Goa, and 24 MSM per hotspot in Maharashtra and Puducherry.

Majority of the network operators were mapped in five States, i.e., Maharashtra (383), Karnataka (198), Madhya Pradesh (161), Tamil Nadu (148) and Delhi (110) where PMPSE mapped at least 100 network operators.

In most states, on an average 1 or 2 MSM operated exclusively in LWS villages. However, in Gujarat, there were 4 MSM per LWS village.

The PMPSE reported presence of MSM in 558 districts of the country (S4 Table). In 7 districts (1-Andhra Pradesh, 2-Gujarat, 2-Maharasthra, 1-Tamil Nadu, and 1-Telangama), PMPSE reported at least 5000 men who have sex with men. MSM in range of 2500 to < 5000 was reported in 20 districts (2-Andhra Pradesh, 2-Assam, 1-Chandigarh, 5-Delhi, 1-Gujarat, 5-Karnataka, 2-Maharasthra, 1-Tamil Nadu, and 1-Telangama). PMPSE reported MSM in range of 1000 to < 2500 in 72 districts, in range of 500 to < 1000 in 81 districts and less than 500 in 378 districts (Table 5). Most districts have MSM size estimates of < 1000 except for some districts in southern, western, central, and eastern region.

### People who inject drugs (PWID)

PMPSE for PWID identified 17,288 hotspots and 953 network operators. PWID were further reported in a total of 2,349 villages. Nearly one-fourth (23.7%) of the PWID hotspots were identified in old or vacant buildings, and abandoned/dilapidated structures, followed by 14.1% in home-based settings and 13.7 street-based locations. A total of 54,221 key informants were interviewed, including, 38,968 from key population groups.

Out of the total estimated 2,88,717 (2,53,025-3,24,408) PWID, around 93% were at hotspots (around 15 PWID per hotspot), 3.5% were exclusively with network operators (around 11 PWID per network operator) and 3.9% (S5 Table) were exclusively in link worker villages (around 5 per LWS village).

Punjab (15.6%), Uttar Pradesh (12.3%), Delhi (11.3%), Assam (9.10%), and Manipur (8.7%) were the States with highest size of PWID contributing around 57% of the total size in the country. Number of PWID per 1000 adult men was highest in Mizoram (31.29), followed by Manipur (28.68), and Nagaland (27.32).

Highest number of hotspots in PMPSE were mapped in Uttar Pradesh (around 14% of the total hotspots), followed by Punjab (around 11%), Manipur and Haryana (8% each). In, Delhi there were around 25 PWID per hotspot, highest in the country followed by 23 PWID per hotspot in Chandigarh and 22 PWID per hotspot in Assam and 21 in Meghalaya.

At least 100 network operators were mapped in the four States of Punjab (162), Jammu & Kashmir (133), Assam (105), and Madhya Pradesh (102), collectively contributing to 53% of the total PWID network operators in the country.

In Punjab, there were 11 PWIDs per LWS village operating only in the given village, followed by 7 in Meghalaya. However, in Manipur (2), Madhya Pradesh (3), Mizoram (3) and Maharashtra (4), an average of 2–4 PWIDs operated exclusively per LWS village.

The PMPSE reported presence of PWIDs in 390 districts of the country (S6 Table). In four districts, PMPSE reported at least 5000 people who inject drugs (1-Assam, 1-Manipur, and 2-Punjab). PWIDs in range of 2500 to < 5000 was reported in 22 districts (1-Assam, 7-Delhi, 2-Haryana, 1-Karnataka, 2-Manipur, 1-Mizoram, 3-Nagaland, 4-Punjab, and 1-Uttar Pradesh). PMPSE reported FSWs in range of 1000 to < 2500 in 59 districts, in range of 500 to < 1000 in 81 districts and less than 500 in 224 districts (Table 7).

### Hijra/transgender people (H/TG)

The PMPSE for H/TG people identified 6,585 hotspots and 720 network operators. H/TG people were further reported in a total of 886 villages. At the national level, 32.8% of the H/TG hotspots were home-based, 11.8% were street-based, 10% were located near bus stands, 6.5% were in marketplaces, 5.5% were near highways, 5% were in abandoned areas, 4.5% were in parks, and 4.1% were near railway stations. A total of 20,775 key informants were interviewed, including, 15,231 from key population groups.

Out of the estimated H/TG people 96,193 (85,206-1,07,174), around 90.8% were at hotspots (around 13 H/TG per hotspot), 8.07% were exclusively with network operators (around 11 H/TG per network operator) and 1.08% (S7 Table) were exclusively in link worker villages (around 1 per LWS village).

Delhi (18.61%), Karnataka (11.35%), Maharashtra (10.73%), Uttar Pradesh (10.23%) and Tamil Nadu (9.57%), were the States with the highest size of H/TG people, contributing around 61% of the total size in the country. Delhi had the highest number of H/TG (2.75) per 1,000 adult men in the country.

Highest number of hotspots in PMPSE were mapped in Uttar Pradesh (around 14% of the total hotspots), followed by Karnataka (around 13%), and 10% each in Odisha, Delhi and Tamil Nadu. In, Telangana there were around 25 H/TG per hotspot, highest in the country, followed by 24 H/TG per hotspot in Delhi, 21 in Gujarat and 20 each per hotspot in Andhra Pradesh and Maharashtra.

In Karnataka (115) and Uttar Pradesh, more than 100 network operators were mapped. This was followed by Odisha (91), Tamil Nadu (59), Delhi (56) and Rajasthan (51) where 50 or more network operators were identified. In other states, less than 10 network operators were mapped.

Except for West Bengal (2), all other states had only 1 H/TG person per LWS village operating exclusively within that village.

The PMPSE estimated presence of H/TG people in 468 districts of the country, mostly with a size estimate of less than 500. Districts not having size estimates for PMPSE were largely in the north-eastern regions but also in eastern, central and northern region (S8 Table). Seventeen districts (1-Andhra Pradesh, 1-Assam, 9-Delhi, 1-Gujarat, 1-Karnataka, 2-Maharasthra, 1-Odisha and 1-Tamil Nadu), PMPSE estimated at least 1000 hijra/transgender people. PMPSE estimated H/TG people in range of in range of 500 to < 1000 in 24 districts and less than 500 in 427 districts (Table 9).

## Discussion

By design, the PMPSE was implemented by community (peer educators and cluster link workers) to estimate the size of key populations, including female sex workers, men who have sex with men, people who inject drugs, and hijra/transgender people, across all Indian States and Union Territories, except for four out of the eight Union Territories. The PMPSE estimated the key population size specifically at hotspots located in urban and peri-urban locations, within the most populated villages, and those operating via network operators in a total of 651 districts. As of now, it stands as the most comprehensive and extensive size estimation activity of key population ever undertaken globally.

Key population size estimates are not a new activity under NACP in India. Yet, historically, the previous initiatives were primarily undertaken by individual States by employing varying methodologies and were primarily concentrated on larger cities [11,21]. On the contrary, PMPSE 2020–22, first of its kind in India, was conducted following uniform methodology across all States/ UTs with a technical support from the national agency. Moreover, PMPSE 2020–22 had a broader geographic scope that went beyond the major urban centres and also had the unique feature of assessment and quantification of key populations linked to network operators for the first time in India. This initiative has been instrumental in revising the coverage targets for key populations at national, state, and district levels and has also provided insights into the evolving trends of partner-seeking via network operators.

While country-wide PMPSE was implemented for the first time in India, it has been recommended and employed extensively in other countries [12,13,22]. African countries like Kenya, Tanzania, Kosovo, South Africa, have implemented one or more rounds of PMPSE for FSWs, MSM, PWID, and transgender persons to study the geographical distribution, size, and operational dynamics [23–27]. In each of these settings, PMPSE has been adopted as a rapid, efficient, systematic, and scientific approach with a specific focus on informing the prevention strategies and service delivery program.

With respect to the size estimates, the PMPSE estimated 15% (4%-25%) higher FSWs, 63% (43% - 83%) higher PWIDs and 37% (22% - 53%) higher hijra/transgender persons than the previous estimates [5]. Higher FSW populations were estimated in the southern and western regions of the country compared to other regions. Most districts have MSM populations of 1,000 or fewer, except for some in the south, west, central, and east. PWID populations were estimated in 393 districts, mainly in the north, east, and north-east. PMPSE assessed the H/TG population in 471 districts, with most reporting fewer than 500 individuals. Districts lacking PMPSE estimates for H/TG people were primarily in the north-east, east, central, and north regions. For MSM, the PMPSE estimates were slightly lower than the previous estimates used by the programme. The higher number of the FSWs, PWIDs and H/TG persons estimated could be due to increased geographical coverage, use of peers and other grass root workers in data collection. The reduction in size estimates of MSM could be due to changes in the approach for seeking partner as there are growing evidence of use of the Virtual Apps by MSM to find partners [28–30].

The number of FSWs per 1000 adult women across various States/UTs ranged from 0.34 to 17.25; MSM numbers spanned from 0.07 to 7.35 per 1000 adult men, H/TG persons ranged from 0.03 to 2.75 per 1000 adult men, and PWIDs ranged between 0.01 and 31.30 per 1000 adult men. The observed variation underscores heterogeneity in the HIV epidemic in India and is consistent with global patterns. The higher proportions of FSWs, MSM, PWIDs, and H/TG persons in some States align with proportions seen in other regions. In Kenya’s PMPSE report from 2018 estimated 11.6 FSWs per 1000 adult females, 2.37 MSM per 1000 adult men, 0.34 transgender individuals per 1000 adult men, and 1.09 PWIDs per 1000 adult men [25]. In Tanzania, the PMPSE estimated that about 6% of adult women were FSWs, whereas approximately 1% of the adult male population identified as MSM [23]. Globally, the size estimates of FSW ranges between 0.59% in Asia and the Pacific to 3.14% in Caribbean, MSM ranges between 1.02% in Middle East and North Africa to 4.5% in Western and central Europe and North America, PWID ranges between 2.99% in Eastern Europe and Central Asia to 0.32% in Latin America and Transgender women ranged between 0.18% in Latin America to 0.36% in Caribbean [31]. As evident, India’s PMPSE for MSM are much lower than the regional averages.

PMPSE (2020–22) has provided critical new evidence of programmatic relevance which is essential for designing and scaling-up the prevention services for key populations in India. For the first time, PMPSE has not only quantified the presence of network operators but also demonstrated that a segment of the key population (approximately 14% of FSWs, 7% of MSM, and 8% of H/TG individuals) can only be reached via these operators. Traditionally, prevention programs under NACP in India have focused on venue-based key populations. However, as indicated by PMPSE findings, prevention services must evolve and adapt to current trends to achieve penetration and expand coverage among network operators.

The PMPSE (2020–22) estimated a significantly larger number of PWIDs, and H/TG individuals compared to previous program estimates. Specifically, in eight north-eastern and northern states, PMPSE estimates for PWIDs are between 2–10 times higher than the earlier figures. For six States, estimates for H/TG people are 2–4 times higher than the previous estimates. Considering that PWIDs and H/TG individuals are among the most affected populations by the HIV/AIDS epidemic in India, these PMPSE findings underscored the urgent need to expand program coverage to these population. Consequently, the PMPSE results were swiftly integrated into the NACP planning process, leading to a significant increase in coverage across risk groups in 2022–23 compared to 2021–22 [5,32].

### Limitations

Key population size estimation is an evolving science with no gold standards and each method having its own strengths and limitations [33,34]. India’s PMPSE (2020–22), despite being the most comprehensive and extensive exercise for estimating key population sizes, is not without its specific limitations.

Firstly, its methodology depends on estimates provided by key informants rather than actual head counts, which may lead to variability (either overestimation or underestimation) compared to the actual size at the location, depending on the quality of knowledge possessed by the participating key informants. Information provided by key informants may also be influenced by recall bias, social desirability bias, or limited visibility of certain groups, this may have resulted in underestimation or overestimation of certain subpopulations. To mitigate this bias, PMPSE had in-built cross-validation mechanism during data collection. Specifically, at least three respondents, including at least two from the community, were involved in providing estimates at the hotspots. The field team facilitated a discussion among key informants to derive an agreed-upon estimate. This process reduced subjective errors, although some degree of bias may still be present.

Additionally, KIs might have reported on the key populations that are frequent or regular participants, potentially overlooking infrequent visitors. Moreover, PMPSE lacked the questions to identify concurrent multiple risk behaviours, such as recognising an individual as an MSM, a sex worker, and PWID simultaneously. Failure to consider infrequent visitors and multiple risk behaviours could result in potential underestimation. Data collection tool shall be modified to account for infrequent visitors and multiple risk behaviours in future rounds of PMPSE.

The PMPSE findings pertain to the size estimates at specific physical locations and with identifiable network operators which could be mapped during the activity following the prescribed methodology. It is possible that certain venues or network operators were extremely hidden and beyond reach and hence could not identified by PMPSE, which also may lead to an underestimation of the size of key populations. Thirdly, the PMPSE lacked a mechanism to estimate the size of key populations that operate via virtual platforms. Consequently, the overall size estimates of these key populations are likely to have been underestimated. Especially for the MSM and H/TG population groups, evidence shows that a significant proportion operate solely through Virtual Apps [35]. Recognising these emerging dynamics to seek partners, NACO is currently implementing pilot project to test the implementation and associated challenges to estimate the size of MSM and H/TG people operating on virtual platforms. The learnings from this exercise will be used in subsequent rounds of PMPSE to account for the size of KPs operating through virtual platforms.

Not having the mechanism to capture the refusal rates was another key limitation of PMPSE in India. The PMPSE was designed to be implemented by the community (peer educators) collecting information from the community (key informants). Since the implementation involved the community, it was assumed at design stage that participation would be universal. Consequently, the design did not include mechanism to document the extent or reasons for refusal. During field implementation, however, it became apparent that there were some refusals for various reasons. We believe that this refusal was minimal and did not affect the survey findings. Nevertheless, the methodology did not have tools to quantify or qualify these refusals, making it impossible to note the non-response rate. Without knowing the refusal rates, it becomes challenging to assess the extent of non-response bias. This point is especially relevant in the context of lower numbers observed in some states, which may be due to the refusal of key informants/network operators. Given the utility of response rate in helping the data interpretation, the methodology to incorporate the same shall be considered in future round of PMPSE.

Another significant limitation of the PMPSE is challenges in generalisability. For instance, the PMPSE estimated approximately ninety thousand FSWs operating specifically within 16,095 villages. However, these findings cannot be extrapolated to other villages because the selected villages were large and part of a link worker programme, which may have distinct characteristics compared to other villages.

The overlap between the implementation of PMPSE and the COVID-19 pandemic introduced several challenges that may have affected the estimates in various ways. The issues began with training, as many States had to resort to virtual platforms, which was not the recommended method. The implementation period was extended due to restricted mobility and movement during the pandemic waves. This situation exposed PMPSE to problems such as turnover among trained staff. Integrating new employees into a trust-based ecosystem, especially when interviewing network operators, became particularly difficult. Additionally, the requirement for written consent forms further complicated an already challenging environment. Although persistent community engagement and perseverance helped mitigate these challenges significantly, the conditions were far from ideal for implementing the first round of PMPSE on such a large scale.

## Conclusion

Despite the challenges and limitations, community-led PMPSE has provided crucial and wide-scale evidence on hotspots and network operators for scaling up HIV prevention services among key populations in India. As part of the integrated and enhanced surveillance & epidemiology framework and embedded within routine program implementation structures, this activity was entirely funded and managed by the national program. The simplicity of PMPSE process will enable its periodic repetition by grass roots workers, which is crucial not only for tracking population size and dynamics but also for providing essential denominator data. We recommend regular PMPSE, including for key population at virtual platforms as well as for clients of sex workers, to provide up-to-date data that enables policymakers to monitor the distribution and dynamics of key populations over time, identify emerging trends, and adjust strategies as needed. Generating up-to-date evidence will also help ensure resources are allocated efficiently, focusing on areas and populations with the greatest need. Additionally, periodic size estimations and using them for designing and expansion of national HIV prevention strategies will further enhance the credibility and reliability of NACP’s work, promoting trust and collaboration with key populations and stakeholders. Overall, periodic PMPSE implementation shall be a fundamental aspect of evidence-based decision-making and an essential element of an adaptable HIV/AIDS response, contributing to India’s goal of ending AIDS as a public health threat by 2030.

## Supporting information

S1 Appendix Hotspot information format. (PDF)

S2 Appendix Network operator format. (PDF)

S3 Appendix Village information format. (PDF)

S4 Appendix Method for calculating weighted proportion of KPs operating at multiple sites in a given district. (PDF)

S5 Appendix Participant information sheet and informed consent form. (PDF)

S6 Appendix Abbreviations. (PDF)

S1 Table State/UT-wise size estimates of FSW (At hotspots, exclusively with network operators, exclusively in LWS villages) and adult women. (PDF)

S2 Table District-wise size estimates of FSW, PMPSE 2020–22. (PDF)

S3 Table State/UT-wise size estimates of MSM (At hotspots, exclusively with network operators, exclusively in LWS villages) and adult women. (PDF)

S4 Table District-wise size estimates of MSM, PMPSE 2020–22. (PDF)

S5 Table State/UT-wise size estimates of PWID (At hotspots, exclusively with network operators, exclusively in LWS villages) and adult women. (PDF)

S6 Table District-wise size estimates of PWID, PMPSE 2020–22. (PDF)

S7 Table State/UT-wise size estimates of H/TG people (At hotspots, exclusively with network operators, exclusively in LWS villages) and adult women. (PDF)

S8 Table District-wise size estimates of H/TG people, PMPSE 2020–22. (PDF)
