# Supplementary material for: Programmatic mapping and population size estimation of key population in India: Method and findings
Source: PLOS Glob Public Health. 2025 May 7;5(5):e0004475. doi: 10.1371/journal.pgph.0004475 (PMC12057993; doi:10.1371/journal.pgph.0004475)
Supplement: S1 Appendix — (PDF) [file pgph.0004475.s001.pdf]

Annexure 1

Tool 1a: Hotspot Information Format (HIF) for FSW

PLEASE REMEMBER: ONE AND ONLY ONE HIF IS TO BE FILLED IN FOR ONE SPECIFIC HOTSPOT, BASED ON DISCUSSIONS WITH MULTIPLE (AT LEAST THREE) KEY INFORMANTS AT THE HOTSPOT. IN CASE MULTIPLE HIGH-RISK GROUPS ARE AVAILABLE AT ONE PARTICULAR HOTSPOT, ONE HIF IS TO BE FILLED IN FOR EACH HRG

**1. State**	
**2. District**	
**3. Hotspot Location**	1. TI Catchment Area<br>2. Non-TI Catchment Area
**4. Name of TI**	
**5. HRG**	1. FSW

**6. Date of Visit 1**		**Date of Visit 2**		**Date of Visit 3**	

**7a. Name of PE/ORW/PO**		**7b. Designation**	1. PO 2. ORW 3. PE
**8. Name of Hotspot**		**9. Hotspot Code**	
**10. Hotspot Coverage**	1. Currently covered by TI	2. Currently not covered by TI (New hotspot)	

**11. Hotspot Type**	1	Brothel	2	Home	3	Bar
	4	Lodge/dhaba/hotel	5	Street	6	Railway station
	7	Bus stand	8	Park	9	Market place
	10	Cinema	11	Abandoned area	12	Under the bridge
	13	Public toilet	14	Highway	15	Spa
	16	Massage parlour	17	Others (Specify)		

**12. Location**	*Please provide detailed address with a couple of clear landmarks*	City		Town		Village	

**13. Status of Hotspot**	1=Active<br>2=Inactive	If Inactive, since when: Month_________ Year _____________<br>Primary reason for inactive hotspot_________________________

14. If Active, whether 1= Accesible, 2= Inaccessible

Sl. No.	HOTSPOT PROFILE		
15.	**Since** how many months/years this hotspot is **operational** (Circle one relevant category)	1. < 3 months<br>2. 3-6 Months<br>3. 7-11 months	4. 1-2 years<br>5. 2-3 years<br>6. 3+ years

** At least three respondents are approached and two of them must be HRG and one from the secondary stakeholders*

**16.**	How many total FSWs are associated with (Solicit at) this particular hotspot?	**Type of Key Informant (KI)**		Min	Max
		KI1	1=HRG, 2=Community gate keeper 3=Others key stakeholder/ informant (Specify)____________________		
		KI2	1=HRG, 2=Community gate keeper 3=Others key stakeholder/ informant (Specify)____________________		
		KI3	1=HRG, 2=Community gate keeper 3=Others key stakeholder/ informant (Specify)____________________		
		KI4	1=HRG, 2=Community gate keeper 3=Others key stakeholder/ informant (Specify)____________________		
		KI5	1=HRG, 2=Community gate keeper 3=Others key stakeholder/ informant (Specify)____________________		
		KI6	1=HRG, 2=Community gate keeper 3=Others key stakeholder/ informant (Specify)____________________		
		**Agreed number of FSW at the hotspot (after consensus)**			

**17.**	What day of the week can we find the maximum number of FSW at the hotspot (**Peak Day**)?<br>**(Multiple Answers Possible)**<br>**CIRCLE AS APPLICABLE**	MONDAY……………………………........ A<br>TUESDAY…………………………......... B<br>WEDNESDAY………………………...…. C<br>THURSDAY………………………........... D<br>FRIDAY ……………………………........E<br>SATURDAY……………………….......… F<br>SUNDAY………………………………..… G<br>ALL DAYS…………………………….. H
**18.**	What is the **peak time** of the day when we find the maximum number of FSW at the hotspot?<br>**(Multiple Answers Possible)**<br>**CIRCLE AS APPLICABLE**	MORNING..A<br>AFTERNOON......................................B<br>EVENING...C<br>NIGHT..D<br>ALL 24 hrs...E
**19.**	Of the FSWs who are associated with this hotspot, how many (min – max) also work at/visit other **hotspots within the district?**	MIN [ ] MAX [ ]
**20.**	Of the HRG who are associated with this hotspot, are (min – max) aged **below 25 years?**	MIN [ ] MAX [ ]
**21.**	Besides FSW, what are other HRG populations associated with this hotspot (multiple response possible)?	2. MSM 3. H/TG<br>4. IDU 9. No other HRG

Information of other spots for new hotspot listing

Please let us know any other place like this in this area, where HRG work/visit.*

	HOTSPOT NAME	ADDRESS	NAMES OF POTENTIAL STAKEHOLDER(S) AND CONTACT DETAILS	TYPOLOGY of HRG# (FSW/ MSM/IDU/TG)	TYPE OF HOTSPOT
1					
2					
3					
4					
5					

* The data collector must define the area according to the target area being covered, e.g. TI- covered area or non-TI covered area. This can be asked, specifying the names of the blocks/wards/cities/villages that data is being collected for.
# If one hotspot has more than one typology, then please mention them in a separate row.

Hotspot Map:

Tool 1b: Hotspot Information Format (HIF) for MSM

PLEASE REMEMBER: ONE AND ONLY ONE HIF IS TO BE FILLED IN FOR ONE SPECIFIC HOTSPOT, BASED ON DISCUSSIONS WITH MULTIPLE (AT LEAST THREE) KEY INFORMANTS AT THE HOTSPOT. IN CASE MULTIPLE HIGH-RISK GROUPS ARE AVAILABLE AT ONE PARTICULAR HOTSPOT, ONE HIF IS TO BE FILLED IN FOR EACH HRG

<table>
<tr><td>1. State</td><td colspan="8"></td></tr>
<tr><td>2. District</td><td colspan="8"></td></tr>
<tr><td>3. Hotspot Location</td><td colspan="8">1. TI Catchment Area
2. Non-TI Catchment Area</td></tr>
<tr><td>4. Name of TI</td><td colspan="8"></td></tr>
<tr><td>5. HRG</td><td colspan="8">2. MSM</td></tr>
<tr><td>6. Date of Visit 1</td><td colspan="2"></td><td>Date of Visit 2</td><td colspan="2"></td><td>Date of Visit 3</td><td colspan="2"></td></tr>
<tr><td>7a. Name of PE/ORW/PO</td><td colspan="3"></td><td colspan="2">7b. Designation</td><td colspan="3">1. PO 2. ORW 3. PE</td></tr>
<tr><td colspan="6">8. Name of Hotspot</td><td colspan="3">9. Hotspot Code</td></tr>
<tr><td>10. Hotspot Coverage</td><td colspan="3">1. Currently covered by TI</td><td colspan="5">2. Currently not covered by TI (New hotspot)</td></tr>
<tr><td rowspan="6">11. Hotspot Type</td><td>1</td><td>Brothel</td><td>2</td><td>Home</td><td>3</td><td colspan="3">Bar</td></tr>
<tr><td>4</td><td>Lodge/dhaba/hotel</td><td>5</td><td>Street</td><td>6</td><td colspan="3">Railway station</td></tr>
<tr><td>7</td><td>Bus Stand</td><td>8</td><td>Park</td><td>9</td><td colspan="3">Market Place</td></tr>
<tr><td>10</td><td>Cinema</td><td>11</td><td>Abandoned area</td><td>12</td><td colspan="3">Under the bridge</td></tr>
<tr><td>13</td><td>Public toilet</td><td>14</td><td>Highway</td><td>15</td><td colspan="3">Spa</td></tr>
<tr><td>16</td><td>Massage parlour</td><td>17</td><td colspan="5">Others (Specify)</td></tr>
<tr><td>12. Location</td><td colspan="3">Please provide detailed address with a couple of clear landmarks</td><td>City</td><td></td><td>Town</td><td></td><td>Village</td></tr>
<tr><td>13. Status of Hotspot</td><td colspan="2">1=Active
2=Inactive</td><td colspan="6">If Inactive, Since when Month_________ Year _____________

Primary reason for inactive hotspot_________________________</td></tr>
<tr><td colspan="9">14. If Active, whether 1= Accessible 2= Inaccessible</td></tr>
</table>

Sl. No.	HOTSPOT PROFILE		
15.	**Since** how many months/years this hotspot is **operational** (Circle one relevant Category)	1. < 3 months<br>2. 3-6 Months<br>3. 7-11 months	4. 1-2 years<br>5. 2-3 years<br>6. 3+ years

** At least three respondents are approached and two of them must be HRG and one from the secondary stakeholders*

<table>
<tr><td rowspan="8">16.</td><td rowspan="8">How many MSM are associated with (Solicit at) this particular hotspot?</td><td colspan="2">Type of Key Informant (KI)</td><td>Min</td><td>Max</td></tr>
<tr><td>KI1</td><td>1=HRG, 2=Community gate keeper 3= Others key stakeholder/ informant (Specify)______________________</td><td></td><td></td></tr>
<tr><td>KI2</td><td>1=HRG, 2=Community gate keeper 3= Others key stakeholder/ informant (Specify)______________________</td><td></td><td></td></tr>
<tr><td>KI3</td><td>1 = HRG, 2 = Community gate keeper 3 = Others key stakeholder/informant (Specify)______________________</td><td></td><td></td></tr>
<tr><td>KI4</td><td>1=HRG, 2=Community gate keeper 3=Others key stakeholder/ informant (Specify)______________________</td><td></td><td></td></tr>
<tr><td>KI5</td><td>1=HRG, 2=Community gate keeper 3=Others key stakeholder/ informant (Specify)______________________</td><td></td><td></td></tr>
<tr><td>KI6</td><td>1=HRG, 2=Community gate keeper 3=Others key stakeholder/ informant (Specify)______________________</td><td></td><td></td></tr>
<tr><td colspan="2">Agreed number of MSM at the hotspot (after consensus)</td><td></td><td></td></tr>
</table>

**17.**	What day of the week can we find the maximum number of MSM at the hotspot (**Peak Day**)?<br>**(Multiple Answers Possible)**<br>**CIRCLE AS APPLICABLE**	MONDAY……………………………......A<br>TUESDAY……………………………........B<br>WEDNESDAY…………………….......…C<br>THURSDAY……………………….…..........D<br>FRIDAY ………………………………........E<br>SATURDAY…………………………........F<br>SUNDAY……………………………......G<br>ALL DAYS………………………………..H
**18.**	What is the **peak time** of the day when we find the maximum number of MSM at the hotspot?<br>**(Multiple Answers Possible)**<br>**CIRCLE AS APPLICABLE**	MORNING..A<br>AFTERNOON......................................B<br>EVENING..C<br>NIGHT..D<br>ALL 24 hrs..E
**19.**	Of the MSM who are associated with this hotspot, how many (min – max) also work at/visit other **hotspots within the district?**	MIN [ ] MAX [ ]
**20.**	Of the MSM who are associated with this hotspot, are (min – max) aged **below 25 years?**	MIN [ ] MAX [ ]
**21.**	Besides MSM, what are other HRG populations associated with this hotspot (multiple response possible)?	1. FSW 3. H/TG<br>4. IDU 9. No other HRG

Information of other spots for new hotspot listing

Please let us know any other place like this in this area, where HRG work/visit.*

	HOTSPOT NAME	ADDRESS	NAMES OF POTENTIAL STAKEHOLDER(S) AND CONTACT DETAILS	TYPOLOGY of HRG # (FSW/ MSM/IDU/TG)	TYPE OF HOTSPOT
1					
2					
3					
4					
5					

* The data collector must define the area according to the target area being covered, e.g. TI- covered area or non-TI covered area. This can be asked, specifying the names of the blocks/wards/cities/villages that data is being collected for.

Hotspot Map:

Tool 1c: Hotspot Information Format (HIF) for Hijra/ TG

PLEASE REMEMBER: ONE AND ONLY ONE HIF IS TO BE FILLED IN FOR ONE SPECIFIC HOTSPOT, BASED ON DISCUSSIONS WITH MULTIPLE (AT LEAST THREE) KEY INFORMANTS AT THE HOTSPOT. IN CASE MULTIPLE HIGH-RISK GROUPS ARE AVAILABLE AT ONE PARTICULAR HOTSPOT, ONE HIF IS TO BE FILLED IN FOR EACH HRG

**1. State**					
**2. District**					
**3. Hotspot Location**	1. TI Catchment Area<br>2. Non-TI Catchment Area				
**4. Name of TI**					
**5. HRG**	3. H/TG				
**6. Date of Visit 1**		**Date of Visit 2**		**Date of Visit 3**	
**7a. Name of PE/ORW/PO**		**7b. Designation**	1. PO 2. ORW 3. PE		
**8. Name of Hotspot**			**9. Hotspot Code**		
**10. Hotspot Coverage**	1. Currently covered by TI	2. Currently not covered by TI (New hotspot)			

**11. Hotspot Type**						
	1	Brothel	2	Home	3	Bar
	4	Lodge/dhaba/hotel	5	Street	6	Railway station
	7	Bus Stand	8	Park	9	Market Place
	10	Cinema	11	Abandoned area	12	Under the bridge
	13	Public toilet	14	Highway	15	Spa
	16	Massage parlour	17	Others (Specify)		

**12. Location**	*Please provide detailed address with a couple of clear landmarks*	**City**		**Town**		**Village**	
**13. Status of Hotspot**	1=Active<br>2=Inactive	If Inactive, Since when Month_________ Year _____________<br>Primary reason for inactive hotspot__________________________					
**14.** If Active, whether 1= Accessible 2= Inaccessible							

**Sl. No.**	**HOTSPOT PROFILE**		
**15.**	**Since** how many months/years this hotspot is **operational** (Circle one relevant Category)	1. < 3 months<br>2. 3-6 Months<br>3. 7-11 months	4. 1-2 years<br>5. 2-3 years<br>6. 3+ years

** At least three respondents are approached and two of them must be HRG and one from the secondary stakeholders*

**16.**	How many H/TG are associated with (Solicit at) this particular hotspot?	**Type of Key Informant (KI)**		Min	Max
		KI1	1=HRG, 2=Community gate keeper 3= Others key stakeholder/ informant (Specify)______________________		
		KI2	1=HRG, 2=Community gate keeper 3= Others key stakeholder/ informant (Specify)______________________		
		KI3	1 = HRG, 2 = Community gate keeper 3 = Others key stakeholder/informant (Specify)______________________		
		KI4	1=HRG, 2=Community gate keeper 3=Others key stakeholder/ informant (Specify)______________________		
		KI5	1=HRG, 2=Community gate keeper 3=Others key stakeholder/ informant (Specify)______________________		
		KI6	1=HRG, 2=Community gate keeper 3=Others key stakeholder/ informant (Specify)______________________		
		**Agreed number of H/TG at the hotspot (after consensus)**			

**17.**	What day of the week can we find the maximum number of H/TG at the hotspot (**Peak Day**)?<br>**(Multiple Answers Possible)**<br>**CIRCLE AS APPLICABLE**	MONDAY…………………………...........A<br>TUESDAY……………………..................B<br>WEDNESDAY……………………........C<br>THURSDAY………………………........... D<br>FRIDAY …………………………......... E<br>SATURDAY………………………........ F<br>SUNDAY………………………………..… G<br>ALL DAYS………………………………..H
**18.**	What is the **peak time** of the day when we find the maximum number of H/TG at the hotspot?<br>**(Multiple Answers Possible)**<br>**CIRCLE AS APPLICABLE**	MORNING...A<br>AFTERNOON.......................................B<br>EVENING..C<br>NIGHT... D<br>ALL 24 hrs..E
**19.**	Of the H/TG who are associated with this hotspot, how many (min – max) also work at/visit other **hotspots within the district, on the same day?**	MIN ☐ MAX ☐
**20.**	Of the H/TG who are associated with this hotspot, are (min – max) aged **below 25 years?**	MIN ☐ MAX ☐
**21.**	Besides H/TG, what are other HRG populations associated with this hotspot (multiple response possible)?	1. FSW 2. MSM<br>4. IDU 9. No other HRG

Information of other spots for new hotspot listing

Please let us know any other place like this in this area, where HRG work/visit.*

	HOTSPOT NAME	ADDRESS	NAMES OF POTENTIAL STAKEHOLDER(S) AND CONTACT DETAILS	TYPOLOGY of HRG # (FSW/ MSM/IDU/TG)	TYPE OF HOTSPOT
1					
2					
3					
4					
5					

* The data collector must define the area according to the target area being covered, e.g. TI- covered area or non-TI covered area. This can be asked, specifying the names of the blocks/wards/cities/villages that data is being collected for.

Hotspot Map:

Tool 1d: Hotspot Information Format (HIF) for IDU

PLEASE REMEMBER: ONE AND ONLY ONE HIF IS TO BE FILLED IN FOR ONE SPECIFIC HOTSPOT, BASED ON DISCUSSIONS WITH MULTIPLE (AT LEAST THREE) KEY INFORMANTS AT THE HOTSPOT. IN CASE MULTIPLE HIGH-RISK GROUPS ARE AVAILABLE AT ONE PARTICULAR HOTSPOT, ONE HIF IS TO BE FILLED IN FOR EACH HRG

<table>
<tr><td>1. State</td><td colspan="3"></td></tr>
<tr><td>2. District</td><td colspan="3"></td></tr>
<tr><td>3. Hotspot Location</td><td colspan="3">1. TI Catchment Area
2. Non-TI Catchment Area</td></tr>
<tr><td>4. Name of TI</td><td colspan="3"></td></tr>
<tr><td>5. HRG</td><td colspan="3">4. IDU</td></tr>
<tr><td>6. Date of Visit 1</td><td></td><td>Date of Visit 2</td><td></td><td>Date of Visit 3</td><td></td></tr>
<tr><td>7a. Name of PE/ORW/PO</td><td></td><td>7b. Designation</td><td colspan="3">1. PO 2. ORW 3. PE</td></tr>
<tr><td colspan="3">8. Name of Hotspot</td><td colspan="3">9. Hotspot Code</td></tr>
<tr><td>10. Hotspot Coverage</td><td colspan="2">1. Currently covered by TI</td><td colspan="3">2. Currently not covered by TI
(New hotspot)</td></tr>
</table>

<table>
<tr><td rowspan="6">11. Hotspot Type</td><td>1</td><td>Brothel</td><td>2</td><td>Home</td><td>3</td><td>Bar</td></tr>
<tr><td>4</td><td>Lodge/dhaba/hotel</td><td>5</td><td>Street</td><td>6</td><td>Railway station</td></tr>
<tr><td>7</td><td>Bus Stand</td><td>8</td><td>Park</td><td>9</td><td>Market Place</td></tr>
<tr><td>10</td><td>Cinema</td><td>11</td><td>Abandoned area</td><td>12</td><td>Under the bridge</td></tr>
<tr><td>13</td><td>Public toilet</td><td>14</td><td>Highway</td><td>15</td><td>Spa</td></tr>
<tr><td>16</td><td>Massage parlour</td><td>17</td><td colspan="3">Others (Specify)</td></tr>
</table>

<table>
<tr><td>12. Location</td><td>Please provide detailed address with a couple of clear landmarks</td><td>City</td><td></td><td>Town</td><td></td><td>Village</td><td></td></tr>
<tr><td>13. Status of Hotspot</td><td>1=Active
2=Inactive</td><td colspan="6">If Inactive, Since when Month__________ Year ______________
Primary reason for inactive hotspot___________________________</td></tr>
<tr><td colspan="8">14. If Active, whether 1= Accessible 2= Inaccessible</td></tr>
</table>

<table>
<tr><th>Sl. No.</th><th colspan="3">HOTSPOT PROFILE</th></tr>
<tr><td>15.</td><td>Since how many months/years this hotspot is operational (Circle one relevant Category)</td><td>1. < 3 months
2. 3-6 Months
3. 7-11 months</td><td>4. 1-2 years
5. 2-3 years
6. 3+ years</td></tr>
<tr><td colspan="4">* At least three respondents are approached and two of them must be HRG and one from the secondary stakeholders</td></tr>
</table>

<table>
<tr><td rowspan="8">16.</td><td rowspan="8">How many IDU are associated with (Solicit at) this particular hotspot?</td><td colspan="2">Type of Key Informant (KI)</td><td>Min</td><td>Max</td></tr>
<tr><td>KI1</td><td>1=HRG, 2=Community gate keeper 3=Others key stakeholder/ informant (Specify)______________________</td><td></td><td></td></tr>
<tr><td>KI2</td><td>1=HRG, 2=Community gate keeper 3=Others key stakeholder/ informant (Specify)______________________</td><td></td><td></td></tr>
<tr><td>KI3</td><td>1=HRG, 2=Community gate keeper 3=Others key stakeholder/ informant (Specify)______________________</td><td></td><td></td></tr>
<tr><td>KI4</td><td>1=HRG, 2=Community gate keeper 3=Others key stakeholder/ informant (Specify)______________________</td><td></td><td></td></tr>
<tr><td>KI5</td><td>1=HRG, 2=Community gate keeper 3=Others key stakeholder/ informant (Specify)______________________</td><td></td><td></td></tr>
<tr><td>KI6</td><td>1=HRG, 2=Community gate keeper 3=Others key stakeholder/ informant (Specify)______________________</td><td></td><td></td></tr>
<tr><td colspan="2">Agreed number of IDU at the hotspot (after consensus)</td><td></td><td></td></tr>
</table>

17.	Of the IDU who are associated with this hotspot, how many are females?	**Type of Key Informant (KI)**	Min	Max
		1=HRG, 2=Community gate keeper 3=Others key stakeholder/informant (Specify)________________		
		1=HRG, 2=Community gate keeper 3=Others key stakeholder/informant (Specify)________________		
		1=HRG, 2=Community gate keeper 3=Others key stakeholder/informant (Specify)________________		
		1=HRG, 2=Community gate keeper 3=Others key stakeholder/informant (Specify)________________		
		1=HRG, 2=Community gate keeper 3=Others key stakeholder/informant (Specify)________________		
		1=HRG, 2=Community gate keeper 3=Others key stakeholder/informant (Specify)________________		
		**Agreed number of HRG (After Consensus)**		

18.	What day of the week can we find the maximum number of IDU at the hotspot (**Peak Day**)? **(Multiple Answers Possible)** **CIRCLE AS APPLICABLE**	MONDAY.. A<br>TUESDAY.. B<br>WEDNESDAY.................................... C<br>THURSDAY....................................... D<br>FRIDAY ....E<br>SATURDAY....................................... F<br>SUNDAY..G<br>ALL DAYS..H
19.	What is the **peak time** of the day when we find the maximum number of IDU at the hotspot? **(Multiple Answers Possible)** **CIRCLE AS APPLICABLE**	MORNING..A<br>AFTERNOON......................................B<br>EVENING...C<br>NIGHT.. D<br>ALL 24 hrs...E
20.	Of the IDU who are associated with this hotspot, how many (min – max) also work at/visit other **hotspots within the district?**	MIN [ ] MAX [ ]
21.	Of the IDU who are associated with this hotspot, are (min – max) aged **below 25 years?**	MIN [ ] MAX [ ]
22.	Besides IDU, what are other HRG populations associated with this hotspot (multiple response possible)?	1. FSW 2. MSM<br>3. H/TG 9. No other HRG

Information of other spots for new hotspot listing

Please let us know any other place like this in this area, where HRG work/visit.*

	HOTSPOT NAME	ADDRESS	NAMES OF POTENTIAL STAKEHOLDER(S) AND CONTACT DETAILS	TYPOLOGY of HRG# (FSW/ MSM/IDU/TG)	TYPE OF HOTSPOT
1					
2					
3					
4					
5					

* The data collector must define the area according to the target area being covered, e.g. TI- covered area or non-TI covered area. This can be asked, specifying the names of the blocks/wards/cities/villages that data is being collected for.

Hotspot Map:
