# Supplementary material for: Programmatic mapping and population size estimation of key population in India: Method and findings
Source: PLOS Glob Public Health. 2025 May 7;5(5):e0004475. doi: 10.1371/journal.pgph.0004475 (PMC12057993; doi:10.1371/journal.pgph.0004475)
Supplement: S2 Appendix — (PDF) [file pgph.0004475.s002.pdf]

Annexure 2

Tool 2a: Network Operator Format for FSW

**1. State**	
**2. District**	
**3. Name of Network Operator**	
**4. Contact Details**	
**5. Gender**	1. Male 2. Female 3. H/TG
**6. Age**	

**Section A: Network Size**		
**Sl. No**	**QUESTION**	**NUMBER**
1	Number of girls currently associated with/in your network	
2	Among the girls in your network, how many of them are in other networks?	
3	Among the girls in your network, how many of them also solicit at any physical hotspot e.g. brothel or street-based site/home/hotel/dhaba etc.	

**Section B (List of Network Operator)**		
Do you know any other network operator? If yes, please provide means to contact him or her.		
**S. No**	**Contact Details**	
A		
B		
C		
D		
E		
F		

Remark if Any:

Counsellor/ORW Name and Signature:

Tool 2b: Network Operator Format for MSM

**1. State**	
**2. District**	
**3. Name of Network Operator**	
**4. Contact Details**	
**5. Gender**	1. Male 2. Female 3. H/TG
**6. Age**	

Section A: Network Size

Sl. No	QUESTION	NUMBER
1	Number of boys/ MSM currently associated with/in your network	
2	Among the boys/ MSM in your network, how many of them are in other networks?	
3	Among the boys/ MSM in your network, how many of them also solicit at any physical hotspot e.g. street-based site/home/hotel/dhaba etc.	

Section B (List of Network Operator)

Do you know any other network operator? If yes, please provide means to contact him or her.

S. No	Contact Details	
A		
B		
C		
D		
E		
F		

Remark if Any:

Counsellor/ORW Name and Signature:

Tool 2c: Network Operator Format H/TG

**1. State**	
**2. District**	
**3. Name of Network Operator**	
**4. Contact Details**	
**5. Gender**	1. Male 2. Female 3. H/TG
**6. Age**	

Section A: Network Size

Sl. No	QUESTION	NUMBER
1	Number of H/TG currently associated with/in your network	
2	Among the H/TG in your network, how many of them are in other networks?	
3	Among the H/TG in your network, how many of them also solicit at any physical hotspot e.g. brothel or street-based site/home/hotel/dhaba etc.	

Section B (List of Network Operator)

Do you know any other network operator? If yes, please provide means to contact him or her.

S. No	Contact Details	
A		
B		
C		
D		
E		
F		

Remark if Any:

Counsellor/ORW Name and Signature:

Tool 2d: Network Operator Format IDU

**1. State**		
**2. District**		
**3. Name of Network Operator**		
**4. Contact Details**		
**5. Gender**	1. Male 2. Female 3. H/TG	
**6. Age**		
**Section A: Network Size**		
**Sl. No**	**QUESTION**	**NUMBER**
1	Number of IDUs currently associated with/in your network	
2	Among the IDUs in your network, how many of them are in other networks?	
3	Among the IDUs in your network, how many of them also inject at any physical hotspot?	
**Section B (List of Network Operator)**		
Do you know any other network operator? If yes, please provide means to contact him or her.		
**S. No**	**Contact Details**	
A		
B		
C		
D		
E		
F		

Remark if Any:

Counsellor/ORW Name and Signature:
