# Supplementary material for: Programmatic mapping and population size estimation of key population in India: Method and findings
Source: PLOS Glob Public Health. 2025 May 7;5(5):e0004475. doi: 10.1371/journal.pgph.0004475 (PMC12057993; doi:10.1371/journal.pgph.0004475)
Supplement: S3 Appendix — (PDF) [file pgph.0004475.s003.pdf]

Annexure 10 Village Information Format

Section A: Village Identification				
Cluster Link Worker				
State				
MPSE Population Type (Encircle all as applicable)	1. FSW	2. MSM	3.H/TG	4. IDU
District				
Block or Mandal or Tehsils				
Village				
Households				
Population				
Village type	Currently covered under LSW		Currently not covered under LSW	

Section B: People engaged in high-risk behaviour in the village	
Number of FSW in the village	
Out of above, number of FSW who go to a nearby urban areas for high-risk behaviour	
Number of MSM in the village	
Out of above, number of MSM who go to a nearby urban areas for high-risk behaviour	
Number of IDU in the village	
Out of above, number of IDU who go to a nearby urban areas for high-risk behaviour	
Number of H/TG in the village	
Out of above, number of H/TG who go to a nearby urban areas for high-risk behaviour	

Section C: Information of other spots for new hotspot listing

Please let us know any other place like this in this area, where HRG work/visit.*

	HOTSPOT NAME	ADDRESS	NAMES OF POTENTIAL STAKEHOLDER(S) AND CONTACT DETAILS	TYPOLOGY of HRG	TYPE OF HOTSPOT
1					
2					
3					
4					
5					

* The data collector must define the area according to the target area being covered, e.g. TI- covered area or non-TI covered area. This can be asked, specifying the names of the blocks/wards/cities/villages that data is being collected for.
