# Supplementary material for: Programmatic mapping and population size estimation of key population in India: Method and findings
Source: PLOS Glob Public Health. 2025 May 7;5(5):e0004475. doi: 10.1371/journal.pgph.0004475 (PMC12057993; doi:10.1371/journal.pgph.0004475)
Supplement: S4 Appendix — (PDF) [file pgph.0004475.s004.pdf]

Calculating Weighted Proportion** (W$_i$) **of KPs Operating at Multiple Sites in a given district

**Hotspots** In a District	**Unadjusted Size of No. of** KPs **(UHS$_i$)**	**Proportion going out (Q)**	**UHS$_i$Q**
A	10	20	200
B	20	10	200
C	30	10	300
D	40	15	600
E	50	15	750
	∑p=150		∑pq=2050

UHS_i**= Unadjusted size of KPs at physical hotspot in a given district**

W_i= ∑UHS_iQ /∑UHS_i *100 = 2050/150*100 = 0.136.
