# Supplementary material for: Programmatic mapping and population size estimation of key population in India: Method and findings
Source: PLOS Glob Public Health. 2025 May 7;5(5):e0004475. doi: 10.1371/journal.pgph.0004475 (PMC12057993; doi:10.1371/journal.pgph.0004475)
Supplement: S5 Appendix — (PDF) [file pgph.0004475.s005.pdf]

Annexure 11 Participant Information Sheet and Informed Consent Form for Key Informant Interview and Group Discussions under p-MPSE

Participant Information Sheet

Through this document, we would like to provide information about programmatic Mapping and Population Size Estimation (p-MPSE) which is being conducted by National AIDS Control Organization (NACO), Ministry of Health & Family Welfare, Govt. of India, the nodal national agency for control of HIV in India. This form explains the purpose and details of this activity and your role and participation in the same. Please read the following information carefully or we will read it for you so that you may understand all this before you decide to participate. After you have understood this information, we will request you to provide consent and participate in the Group Discussion (GD) or Key Informant Interview (KII). If you require, we can provide you with a copy of a signed consent form.

NACO is conducting a programmatic MPSE to estimate the size of population groups of Female Sex Workers (FSW), Men who have Sex with Men (MSM), Intravenous Drug Users (IDU), and Hijra/Transgender (H/TG) people so that government can take appropriate actions to augment prevention, detection and treatment related HIV/AIDS services among these population.

This activity is being carried across all States of India. In order to ensure that the size estimates are robust, p-MPSE aims to identify comprehensively all the hotspots/ areas of congregation of the community in this area. We are approaching you, as you are either a member of the concerned community, or closely engaged with them, or you are involved in provision of prevention care and other services to their members and hence would be able to provide key and pertinent information about the locations of hotspots, help plan field work and understand the challenges and needs of community.

Your participation: If you volunteer to participate, you will be asked to take part in a one-time in-depth interview or group discussion, which may take around 1-2 hours. You may leave at any time during the interview/discussion. The information you give will be kept fully confidential, and hence you can answer our questions without any hesitation. We may record the discussion using audio recorder and take notes because we do not want to miss any of the information provided by you. The information you provide will not be linked to your name/alias/position and we will not ask you to state your name in the discussion. You do not have to answer any questions if you do not want to do so. However, your honest answers to these questions will help us better understand the community and plan the surveillance-survey in a better way.

Confidentiality: Please note that all the information provided by you will be kept completely confidential and will not be shared with anyone outside the implementation team. Your name will only be collected on consent form but not in the data form. We are trained to maintain confidentiality of data and conversations with you and will not disclose. Reports/publications discussions that come out of this activity will not have your name or any information that might be used to identify you.

Possible risks and discomforts: We will make every effort to protect your privacy and confidentiality during discussions. By participating, no risk to you is expected but you may feel uncomfortable speaking in front of others. However, it is possible that others may learn of your participation and may treat you unfairly or discriminate against you. Though we don't anticipate any risk to you due to your participation, we have taken adequate care to ensure that you don't face any trouble. In case you face any trouble due to your participation, you are requested to immediately report the same as per the details given below and adequate and appropriate care will be given to you.

Possible Benefits: Though there is no direct benefit to you, by answering these questions, you will help us plan better for the survey. Results from this survey will help the government to improve and augment programmes to prevent HIV/AIDS in not only in your community but also in India as a whole. If you refuse to answer our questions, no harm will come to you or your community, or it will not affect provision of any service under any government programme.

Voluntariness: Your participation in this exercise is entirely voluntary. It is your choice whether to participate or not. If you wish not to take part, you can freely do so, we respect your rights. Additionally, you may also stop participating in the discussion any time you choose. Your refusal to participate will not affect the provision of standard health care services offered to you at this or other government facilities.

Compensation: There is no compensation for you to participate in this exercise.

Contact Details: If you ever have any question about this survey, or if you face any trouble due to your participation in the discussion, you are requested to immediately contact: Dr Pradeep Kumar, Programme Officer (Surveillance), National AIDS Control Organization, New Delhi – 110001, Tel. – 011- 43509906

If you have any questions about your rights as survey participant you can contact: ______________ ______________________, Phone No. – ________________________

Do you have any Questions?

If you are now willing to participate in this discussion, we request you to sign/provide your thumb impression with date in the informed consent form below.

Informed Consent Form

I, ____, aged _________ yrs*, have read the foregoing information, or it has been explained to me in the language I understand. I have had the opportunity to ask questions and all my questions have been answered satisfactorily. I have fully understood all the information, benefits and risks associated with participation in this exercise. I understand that I can withdraw my participation anytime, for any reason. I have understood my role in this programmatic MPSE exercise including the method of data collection and willingly agree to participate and respond to the questions asked. I also know that the information collected from me will be kept anonymous and confidential. I understand that after combining with information from other respondents, it will be utilized by the NACO, Government of India for improving programmes or for reporting. I provide my consent for publication/dissemination of anonymized and combined data resulting from my participation.

Signature/ thumb impression: ______________________________________ Date: ___________

This is the left thumb impression of ____

Name of witness: ____

Signature: ___________________ Date: ___________

(Signature of witness is required if the respondent is illiterate. Witness should be literate and not related to Investigator)

Investigators Name: _____________________________________

Signature: ___________________ Date: ____________
