# Supplementary material for: Programmatic mapping and population size estimation of key population in India: Method and findings
Source: PLOS Glob Public Health. 2025 May 7;5(5):e0004475. doi: 10.1371/journal.pgph.0004475 (PMC12057993; doi:10.1371/journal.pgph.0004475)
Supplement: S6 Appendix — (PDF) [file pgph.0004475.s006.pdf]

Table of abbreviations

AIDS: Acquired immune deficiency syndrome

CAB: Community Advisory Boards

CBO: Community-based Organisations

CLW: Cluster Link Workers

FSW: Female sex workers

HIF: Hotspot Information Format

HIV: Human immunodeficiency virus

H/TG: Hijra/transgender people

ICF: Informed consent form

KP: Key populations

LWS: Link Workers Scheme

M&E: Monitoring and evaluation

MTA: Mid-term appraisal

MSM: Men who have sex with men

NACP: National AIDS and STD Control Program

NACO: National AIDS Control Organization

NGO: Non-Government Organisations

NOF: Network Operator Format

PE: Peer Educators

PIS: Participants information sheet

PMPSE: Programmatic mapping and population size estimation

PLHIV: People living with HIV

PSE: Population Size Estimation

PWID: People who inject drugs

RFA: Rapid Field Assessment

SACS: State AIDS Control Societies

TI: Targeted Interventions

TSU: Technical Support Units

UT: Union territories

VIF: Village Information Format
