# Supplementary material for: Programmatic mapping and population size estimation of key population in India: Method and findings
Source: PLOS Glob Public Health. 2025 May 7;5(5):e0004475. doi: 10.1371/journal.pgph.0004475 (PMC12057993; doi:10.1371/journal.pgph.0004475)
Supplement: S1 Table — (PDF) [file pgph.0004475.s007.pdf]

Supplementary Table S1. State/UT-wise size estimates of FSW (At hotspots, exclusively with network operators, exclusively in LWS villages) and adult women

State/UT	FSW Size Estimates			Adult Women Size
	At hotspots	Exclusively with network operators	Exclusively in LWS villages	
Andhra Pradesh	102512	861	15994	14761003
Arunachal Pradesh	6941	0	0	402358
Assam	38634	1087	0	9739000
Bihar	9253	320	2109	30446999
Chandigarh	3051	282	0	329843
Chhattisgarh	17008	422	945	7943002
Delhi	7539	80860	0	5719001
Goa	5037	4	0	431998
Gujarat	29999	1592	5527	18131001
Haryana	16171	1497	0	7758002
Himachal Pradesh	13083	128	0	2002001
Jammu And Kashmir	4239	395	0	3674000
Jharkhand	11860	0	0	10212001
Karnataka	114171	19975	19191	18382004
Kerala	16055	568	0	9221002
Madhya Pradesh	46054	4651	2750	21811001
Maharashtra	76014	10637	8700	33414000
Manipur	5343	40	279	876597
Meghalaya	2963	61	273	831046
Mizoram	1065	0	369	327580
Nagaland	2139	107	0	585081
Odisha	21820	980	1820	12483002
Puducherry	2427	87	0	469338
Punjab	22696	2133	2475	8038001
Rajasthan	19156	1878	0	20778000
Sikkim	732	0	0	187341
Tamil Nadu	47452	3091	10232	20866000
Telangana	61900	3390	10091	10780002
Tripura	5490	159	593	1129363
Uttar Pradesh	35315	3021	2144	59864998
Uttarakhand	7094	119	0	3163000
West Bengal	18165	590	1697	27339000
India	771375	138935	85189	362096565
