# Supplementary material for: Programmatic mapping and population size estimation of key population in India: Method and findings
Source: PLOS Glob Public Health. 2025 May 7;5(5):e0004475. doi: 10.1371/journal.pgph.0004475 (PMC12057993; doi:10.1371/journal.pgph.0004475)
Supplement: S2 Table — (PDF) [file pgph.0004475.s008.pdf]

Supplementary Table S1. District-wise size estimates of FSW, PMPSE 2020-22

State	Distict	Number of Hot-Spots	Number of Network Operators	Villages	Estimates
Andhra Pradesh	Anantapur	206	17	103	4452 (4075-4828)
Andhra Pradesh	Annamayya	232	2	60	3421 (2893-3949)
Andhra Pradesh	Bapatla	130	4		3873 (3535-4211)
Andhra Pradesh	Chittoor	185	10		2343 (1987-2700)
Andhra Pradesh	East Godavari	30			928 (764-1092)
Andhra Pradesh	Eluru	32		115	1548 (1451-1645)
Andhra Pradesh	Guntur	177		95	4094 (3379-4809)
Andhra Pradesh	Kakinada	175	12	111	4682 (4083-5280)
Andhra Pradesh	Konaseema	32	6		959 (835-1084)
Andhra Pradesh	Krishna	365	37	108	15437 (13309-17565)
Andhra Pradesh	Kurnool	332	15	159	17274 (14933-19614)
Andhra Pradesh	Nandyal	393	17		26587 (22637-30537)
Andhra Pradesh	Palnadu	124			3320 (2791-3849)
Andhra Pradesh	Prakasam	114	4	107	3829 (3515-4143)
Andhra Pradesh	Spsr Nellore	166	1	136	5568 (4842-6293)
Andhra Pradesh	Sri Satya Sai	187	15		4373 (3773-4974)
Andhra Pradesh	Srikakulam	85	1	132	1736 (1411-2061)
Andhra Pradesh	Visakhapatanam	269	6	178	5580 (5097-6064)
Andhra Pradesh	Vizianagaram	132	1	140	2337 (1886-2789)
Andhra Pradesh	West Godavari	92			2855 (2166-3544)
Andhra Pradesh	Y.S.R.	135		154	4170 (3524-4816)
Arunachal Pradesh	Changlang	25			310 (271-349)
Arunachal Pradesh	East Kameng	23			317 (285-349)
Arunachal Pradesh	East Siang	23			293 (269-316)
Arunachal Pradesh	Leparada	13			212 (196-229)
Arunachal Pradesh	Lohit	67			602 (544-659)
Arunachal Pradesh	Lower Dibang Valley	8			62 (57-68)

State	Distict	Number of Hot-Spots	Number of Network Operators	Villages	Estimates
Arunachal Pradesh	Lower Siang	17			268 (242-294)
Arunachal Pradesh	Lower Subansiri	56			542 (471-613)
Arunachal Pradesh	Namsai	74			788 (728-848)
Arunachal Pradesh	Papum Pare	130			1483 (1310-1657)
Arunachal Pradesh	Siang	5			33 (30-35)
Arunachal Pradesh	Tirap	10			197 (181-212)
Arunachal Pradesh	Upper Siang	22			306 (286-326)
Arunachal Pradesh	Upper Subansiri	32			385 (335-435)
Arunachal Pradesh	West Kameng	26			505 (461-549)
Arunachal Pradesh	West Siang	39			639 (598-680)
Assam	Barpeta	105	1		2183 (1949-2418)
Assam	Bongaigaon	64			1695 (1512-1878)
Assam	Cachar	141	6		2316 (2063-2568)
Assam	Chirang	22			525 (468-581)
Assam	Darrang	101			1914 (1691-2136)
Assam	Dhemaji	81	15		1081 (966-1196)
Assam	Dhubri	88	4		1733 (1549-1916)
Assam	Dibrugarh	221			2481 (1678-3285)
Assam	Dima Hasao	17			256 (227-285)
Assam	East Karbi Anglong	90	16		2256 (1991-2521)
Assam	Goalpara	53	6		1529 (1413-1645)
Assam	Golaghat	97	9		1018 (901-1134)
Assam	Hailakandi	32			683 (622-743)
Assam	Hojai	26			742 (638-847)
Assam	Jorhat	41	21		841 (787-894)
Assam	Kamrup	49	1		1832 (1517-2147)
Assam	Kamrup Metro	245	59		3553 (3102-4004)
Assam	Lakhimpur	38	11		750 (655-845)
Assam	Majuli	77			634 (500-767)

State	Distict	Number of Hot-Spots	Number of Network Operators	Villages	Estimates
Assam	Marigaon	88	12		954 (871-1037)
Assam	Nagaon	81	10		2066 (1894-2237)
Assam	Nalbari	54	1		2532 (2447-2617)
Assam	Sivasagar	50	3		2485 (2277-2693)
Assam	Sonitpur	97	6		1591 (1288-1893)
Assam	Tinsukia	56			1829 (1689-1969)
Assam	Udalguri	20	1		244 (194-293)
Bihar	Aurangabad	8			179 (170-188)
Bihar	Begusarai	71	6	100	2149 (2039-2258)
Bihar	Bhojpur	25	3		590 (566-613)
Bihar	Buxar	10	3		233 (218-248)
Bihar	Darbhanga	36		100	624 (604-644)
Bihar	Kaimur (Bhabua)	3	1		42 (31-53)
Bihar	Lakhisarai	31	2		642 (593-691)
Bihar	Nalanda	38	4		755 (709-800)
Bihar	Patna	14	3		421 (398-445)
Bihar	Purbi Champaran	61	2		1078 (984-1173)
Bihar	Purnia	19	5		399 (358-441)
Bihar	Rohtas	62	3		1266 (1182-1350)
Bihar	Saran	33	1	100	920 (885-955)
Bihar	Sheikhpura	6	1		184 (173-195)
Bihar	Sheohar	9			152 (140-164)
Bihar	Sitamarhi	33		100	772 (766-778)
Bihar	Siwan	34	3		744 (706-782)
Bihar	Vaishali	23	2		531 (497-564)
Chandigarh	Chandigarh	131	44		3333 (2997-3668)
Chhattisgarh	Balod	21	2		182 (167-197)
Chhattisgarh	Baloda Bazar	43	3		929 (850-1009)
Chhattisgarh	Bastar	105	5		1772 (1642-1901)

State	Distict	Number of Hot-Spots	Number of Network Operators	Villages	Estimates
Chhattisgarh	Bemetara	7			135 (129-140)
Chhattisgarh	Bilaspur	47	3	64	1393 (1248-1538)
Chhattisgarh	Dantewada	49	5		927 (816-1037)
Chhattisgarh	Dhamtari	26	5		606 (570-642)
Chhattisgarh	Durg	33		98	1077 (1006-1149)
Chhattisgarh	Gariyaband	6			142 (134-150)
Chhattisgarh	Gaurela-Pendra-Marwahi	6	1		69 (63-76)
Chhattisgarh	Janjgir-Champa	25	2		392 (343-442)
Chhattisgarh	Jashpur	46	5		806 (744-867)
Chhattisgarh	Kabirdham	82	2		1444 (1347-1541)
Chhattisgarh	Kanker	43			920 (850-990)
Chhattisgarh	Korba	32	7		580 (513-647)
Chhattisgarh	Korea	27	2		520 (479-560)
Chhattisgarh	Mahasamund	12			144 (131-157)
Chhattisgarh	Mungeli	21	1		475 (426-524)
Chhattisgarh	Raigarh	65	7		1054 (946-1161)
Chhattisgarh	Raipur	131	6	63	2726 (2525-2928)
Chhattisgarh	Rajnandgaon	42	7	41	924 (875-972)
Chhattisgarh	Surajpur	24	2		386 (344-429)
Chhattisgarh	Surguja	36	3		772 (711-833)
Delhi	Central	94	353		12261 (12083-12438)
Delhi	East		370		11309 (11309-11309)
Delhi	New Delhi	12	81		2210 (2197-2224)
Delhi	North	35	176		7020 (6967-7072)
Delhi	North East	5	227		10760 (10747-10772)
Delhi	North West	14	366		11327 (11307-11348)
Delhi	Shahdara		79		2648 (2648-2648)
Delhi	South	16	298		5856 (5809-5902)
Delhi	South East	11	389		6774 (6752-6797)

State	Distict	Number of Hot-Spots	Number of Network Operators	Villages	Estimates
Delhi	South West	54	161		7032 (6895-7169)
Delhi	West	50	345		11203 (11097-11310)
Goa	North Goa	182	5		3907 (3313-4501)
Goa	South Goa	66			1133 (992-1275)
Gujarat	Ahmadabad	142		88	2383 (2130-2636)
Gujarat	Amreli	44		99	802 (762-843)
Gujarat	Anand	35		51	987 (878-1096)
Gujarat	Arvalli	33		62	832 (779-884)
Gujarat	Banas Kantha	38		126	1591 (1413-1769)
Gujarat	Bharuch	44	6		561 (470-652)
Gujarat	Bhavnagar	122		87	5104 (4815-5393)
Gujarat	Botad			12	81 (81-81)
Gujarat	Chhotaudepur	44		88	1054 (924-1185)
Gujarat	Dang	1			9 (8-9)
Gujarat	Devbhumi Dwarka	15			795 (753-837)
Gujarat	Dohad	57			1025 (846-1203)
Gujarat	Gandhinagar	25		56	630 (557-704)
Gujarat	Gir Somnath	21			571 (528-614)
Gujarat	Jamnagar	23			653 (601-705)
Gujarat	Junagadh	34			494 (436-552)
Gujarat	Kachchh	48		114	1156 (1063-1249)
Gujarat	Kheda	20		50	484 (463-504)
Gujarat	Mahesana	46		120	1083 (1012-1153)
Gujarat	Morbi	16			279 (243-315)
Gujarat	Narmada	3			164 (147-181)
Gujarat	Navsari	53			677 (564-790)
Gujarat	Panch Mahals	30			330 (275-385)
Gujarat	Patan	48			475 (415-535)
Gujarat	Porbandar	15			393 (355-431)

State	Distict	Number of Hot-Spots	Number of Network Operators	Villages	Estimates
Gujarat	Rajkot	50			957 (835-1078)
Gujarat	Sabar Kantha	35		63	1034 (978-1091)
Gujarat	Surat	370	75		9136 (8105-10166)
Gujarat	Surendranagar	13			278 (251-304)
Gujarat	Tapi	42			651 (604-697)
Gujarat	Vadodara	83		59	2043 (1872-2213)
Gujarat	Valsad	30			409 (281-537)
Haryana	Ambala	110	10		793 (693-894)
Haryana	Bhiwani	31	8		556 (499-614)
Haryana	Faridabad	59	38		1283 (1199-1368)
Haryana	Fatehabad	98	8		1134 (1031-1237)
Haryana	Gurugram	77	31		2017 (1897-2137)
Haryana	Hisar	41			321 (285-357)
Haryana	Jhajjar	31	2		231 (206-255)
Haryana	Jind	53			565 (461-669)
Haryana	Kaithal	100	6		1117 (979-1254)
Haryana	Karnal	86	3		753 (674-833)
Haryana	Kurukshetra	60	19		983 (896-1069)
Haryana	Mewat	28			664 (587-741)
Haryana	Palwal	54	13		1230 (1109-1351)
Haryana	Panchkula	63	25		934 (857-1010)
Haryana	Panipat	54	5		673 (611-736)
Haryana	Rewari	12	7		334 (310-359)
Haryana	Rohtak	68			664 (601-726)
Haryana	Sirsa	81	30		1176 (1106-1246)
Haryana	Sonipat	100	12		916 (862-969)
Haryana	Yamunanagar	120	4		1323 (1220-1425)
Himachal Pradesh	Bilaspur_HP	59	8		740 (678-803)
Himachal Pradesh	Chamba	78	5		992 (871-1114)

State	Distict	Number of Hot-Spots	Number of Network Operators	Villages	Estimates
Himachal Pradesh	Hamirpur	73	2		1258 (1203-1314)
Himachal Pradesh	Kangra	140			1858 (1634-2081)
Himachal Pradesh	Kullu	74	7		1187 (1087-1287)
Himachal Pradesh	Mandi	127	3		1533 (1361-1705)
Himachal Pradesh	Shimla	113	1		1860 (1632-2087)
Himachal Pradesh	Sirmaur	68			1144 (998-1290)
Himachal Pradesh	Solan	66			1618 (1501-1735)
Himachal Pradesh	Una	115	1		1020 (922-1117)
Jammu And Kashmir	Anantnag	62	11		949 (842-1055)
Jammu And Kashmir	Badgam	9			56 (26-85)
Jammu And Kashmir	Bandipora	1			11 (5-16)
Jammu And Kashmir	Baramulla	10	2		202 (118-286)
Jammu And Kashmir	Doda	4			72 (63-80)
Jammu And Kashmir	Ganderbal	6			47 (19-76)
Jammu And Kashmir	Jammu	26	8		529 (464-594)
Jammu And Kashmir	Kathua	54	9		869 (768-970)
Jammu And Kashmir	Kulgam	30	4		402 (356-448)
Jammu And Kashmir	Kupwara	3			21 (11-30)
Jammu And Kashmir	Poonch	2	1		52 (47-58)
Jammu And Kashmir	Pulwama	11			91 (76-106)
Jammu And Kashmir	Rajauri	10	3		218 (195-242)
Jammu And Kashmir	Reasi	6	2		121 (108-134)
Jammu And Kashmir	Samba	7			98 (85-112)
Jammu And Kashmir	Shopian	16	4		270 (246-293)
Jammu And Kashmir	Srinagar	31	2		319 (197-442)
Jammu And Kashmir	Udhampur	15	6		308 (273-342)
Jharkhand	Bokaro	64			930 (810-1050)
Jharkhand	Chatra	33			430 (400-460)
Jharkhand	Deoghar	101			847 (801-892)

State	Distict	Number of Hot-Spots	Number of Network Operators	Villages	Estimates
Jharkhand	Dhanbad	39			742 (665-820)
Jharkhand	East Singhbum	77			1301 (1152-1450)
Jharkhand	Garhwa	38			784 (701-867)
Jharkhand	Giridih	77			736 (676-797)
Jharkhand	Godda	29			447 (390-504)
Jharkhand	Hazaribagh	35			505 (407-603)
Jharkhand	Koderma	32			772 (723-821)
Jharkhand	Latehar	9			167 (153-182)
Jharkhand	Lohardaga	37			440 (373-506)
Jharkhand	Pakur	71			597 (520-674)
Jharkhand	Palamu	39			710 (635-784)
Jharkhand	Ramgarh	6			101 (83-120)
Jharkhand	Ranchi	46			781 (717-844)
Jharkhand	Sahebganj	31			587 (527-646)
Jharkhand	Saraikela Kharsawan	24			262 (221-302)
Jharkhand	Simdega	28			287 (248-325)
Jharkhand	West Singhbhum	30			436 (367-505)
Karnataka	Bagalkot	211	202	98	9756 (9370-10142)
Karnataka	Ballari	1,529	241	151	14413 (13592-15235)
Karnataka	Belagavi	394	286	100	18321 (17602-19041)
Karnataka	Bengaluru Rural	108	35		1086 (963-1209)
Karnataka	Bengaluru Urban	2,132	967		26013 (22871-29155)
Karnataka	Bidar	121	29		3343 (3194-3492)
Karnataka	Chamarajanagar	275	60	190	8174 (7687-8660)
Karnataka	Chikballapur	268	57	100	4111 (3565-4657)
Karnataka	Chikkamagaluru	152	42		2461 (2081-2841)
Karnataka	Chitradurga	183	92		3564 (3272-3856)
Karnataka	Dakshin Kannad	97	14		1351 (1244-1458)
Karnataka	Davangere	179	64		4421 (3995-4847)

State	Distict	Number of Hot-Spots	Number of Network Operators	Villages	Estimates
Karnataka	Dharwad	107	8		2154 (1963-2345)
Karnataka	Gadag	89	11	139	4108 (3947-4268)
Karnataka	Hassan	138	8	135	2832 (2637-3027)
Karnataka	Haveri	162	18		3004 (2751-3257)
Karnataka	Kalaburagi	96	96		4572 (4272-4871)
Karnataka	Kodagu	102	21		1185 (1085-1285)
Karnataka	Kolar	236	77		4571 (3807-5336)
Karnataka	Koppal	158	72		2755 (2551-2960)
Karnataka	Mandya	105	20	130	6049 (5833-6266)
Karnataka	Mysuru	484	55		4573 (3993-5152)
Karnataka	Raichur	221	35		3129 (2823-3435)
Karnataka	Ramanagara	55	11		601 (542-660)
Karnataka	Shivamogga	147	14		2509 (2190-2828)
Karnataka	Tumakuru	460			2499 (2162-2836)
Karnataka	Udupi	41			455 (390-519)
Karnataka	Uttar Kannad	323	83		3755 (2973-4538)
Karnataka	Vijayapura	121	19	136	5853 (5640-6065)
Karnataka	Yadgir	77	81		1719 (1580-1857)
Kerala	Alappuzha	109	14		850 (755-944)
Kerala	Ernakulam	64	6		835 (671-999)
Kerala	Idukki	56	5		1074 (680-1467)
Kerala	Kannur	53	12		1007 (957-1057)
Kerala	Kasaragod	90	9		751 (626-875)
Kerala	Kollam	154	11		1887 (1685-2089)
Kerala	Kottayam	77	5		1164 (1025-1302)
Kerala	Kozhikode	138	3		1698 (1526-1869)
Kerala	Malappuram	104	5		733 (629-836)
Kerala	Palakkad	94			921 (809-1032)
Kerala	Pathanamthitta	44	8		743 (632-853)

State	Distict	Number of Hot-Spots	Number of Network Operators	Villages	Estimates
Kerala	Thiruvananthapuram	390	22		2723 (2363-3083)
Kerala	Thrissur	61			924 (806-1042)
Kerala	Wayanad	85	7		1313 (1152-1474)
Madhya Pradesh	Agar Malwa	18	6		295 (265-324)
Madhya Pradesh	Alirajpur	45	12		996 (883-1109)
Madhya Pradesh	Anuppur	3			51 (43-59)
Madhya Pradesh	Ashoknagar	32	2		352 (322-381)
Madhya Pradesh	Balaghat	87	25	115	2270 (2146-2394)
Madhya Pradesh	Barwani	54	7	160	764 (676-851)
Madhya Pradesh	Betul	53	22		1134 (1039-1228)
Madhya Pradesh	Bhind	40	12	80	253 (231-275)
Madhya Pradesh	Bhopal	105	16		1710 (1493-1926)
Madhya Pradesh	Burhanpur	46	3		695 (641-750)
Madhya Pradesh	Chhatarpur	70	15		1651 (1515-1787)
Madhya Pradesh	Chhindwara	188	22	140	3599 (3124-4073)
Madhya Pradesh	Damoh	19			99 (48-149)
Madhya Pradesh	Datia	76	5		1064 (857-1271)
Madhya Pradesh	Dewas	58	12		954 (899-1008)
Madhya Pradesh	Dhar	79	12		1322 (1163-1482)
Madhya Pradesh	East Nimar	13	2		303 (262-343)
Madhya Pradesh	Guna	33	6		684 (544-824)
Madhya Pradesh	Gwalior	69	9		1223 (1023-1423)
Madhya Pradesh	Harda	5	3		82 (73-90)
Madhya Pradesh	Hoshangabad	51	17		1146 (1068-1223)
Madhya Pradesh	Indore	175	75		4385 (3856-4914)
Madhya Pradesh	Jabalpur	42	20	42	965 (836-1094)
Madhya Pradesh	Jhabua	32	17		832 (760-905)
Madhya Pradesh	Katni	39	4		533 (477-589)
Madhya Pradesh	Khargone	37	1		794 (712-875)

State	Distict	Number of Hot-Spots	Number of Network Operators	Villages	Estimates
Madhya Pradesh	Mandla	37	4	131	1187 (1105-1269)
Madhya Pradesh	Mandsaur	33	13	73	1584 (1506-1662)
Madhya Pradesh	Morena	45	14		848 (682-1013)
Madhya Pradesh	Narsinghpur	30	2		504 (457-550)
Madhya Pradesh	Neemuch	36	11		855 (758-951)
Madhya Pradesh	Panna	90	3		1897 (1684-2110)
Madhya Pradesh	Raisen	119	43		1925 (1795-2055)
Madhya Pradesh	Rajgarh	20	3		269 (233-306)
Madhya Pradesh	Ratlam	43	26	70	1212 (1131-1293)
Madhya Pradesh	Rewa	61	10		1393 (1230-1556)
Madhya Pradesh	Sagar	156	7		1633 (1114-2152)
Madhya Pradesh	Satna	75	4		1113 (971-1254)
Madhya Pradesh	Sehore	22	8		447 (397-497)
Madhya Pradesh	Seoni	37	12		687 (623-750)
Madhya Pradesh	Shahdol	26	1		433 (374-491)
Madhya Pradesh	Shajapur	39	15		709 (585-834)
Madhya Pradesh	Sheopur	35	8		733 (642-823)
Madhya Pradesh	Shivpuri	96	28		2247 (1952-2541)
Madhya Pradesh	Sidhi	40	5		425 (348-501)
Madhya Pradesh	Singrauli	61	3		937 (772-1103)
Madhya Pradesh	Tikamgarh	93	26	142	1180 (1033-1328)
Madhya Pradesh	Ujjain	93	30	193	1566 (1444-1688)
Madhya Pradesh	Umaria	18	1		262 (229-294)
Madhya Pradesh	Vidisha	67	15		1259 (1167-1351)
Maharashtra	Ahmednagar	66	15		2182 (1991-2372)
Maharashtra	Akola	75	3	128	2487 (2303-2670)
Maharashtra	Amravati	124	751	136	9587 (9457-9716)
Maharashtra	Aurangabad	95	14	156	3258 (3123-3393)
Maharashtra	Beed	131	3		1771 (1596-1947)

State	Distict	Number of Hot-Spots	Number of Network Operators	Villages	Estimates
Maharashtra	Buldhana	55	1	198	1780 (1669-1890)
Maharashtra	Chandrapur	25	32	114	1473 (1405-1542)
Maharashtra	Dhule	65	1	103	923 (891-955)
Maharashtra	Gondia	69	7		810 (755-866)
Maharashtra	Hingoli	23	5		855 (812-899)
Maharashtra	Jalgaon	154	11	104	2052 (1884-2221)
Maharashtra	Jalna	37	17	90	654 (574-734)
Maharashtra	Kolhapur	49	3	131	1144 (990-1298)
Maharashtra	Latur	57	31		1238 (1139-1337)
Maharashtra	Nagpur	116	78	120	5655 (5312-5997)
Maharashtra	Nanded	85	81	102	1860 (1763-1958)
Maharashtra	Nandurbar	113	6		1000 (901-1099)
Maharashtra	Nashik	135	4	99	1233 (1140-1326)
Maharashtra	Osmanabad	82	18		1090 (974-1206)
Maharashtra	Parbhani	65	13	131	1630 (1522-1737)
Maharashtra	Pune	97		90	4999 (4662-5337)
Maharashtra	Raigad	51	4	83	2653 (2418-2889)
Maharashtra	Ratnagiri	2	1		26 (23-28)
Maharashtra	Sangli	68	24	160	2252 (2066-2438)
Maharashtra	Satara	25	9	90	583 (545-620)
Maharashtra	Sindhudurg	25	4		108 (98-118)
Maharashtra	Solapur	175	25		2873 (2527-3220)
Maharashtra	Thane	479	27		15466 (13781-17151)
Maharashtra	Wardha	53	53	180	2436 (2367-2504)
Maharashtra	Washim	9	4	173	863 (851-874)
Maharashtra	Yavatmal	88			2158 (1970-2345)
Manipur	Bishnupur	39		90	667 (628-706)
Manipur	Chandel	21		32	242 (224-261)
Manipur	Churachandpur	46			706 (265-1148)

State	Distict	Number of Hot-Spots	Number of Network Operators	Villages	Estimates
Manipur	Imphal East			52	7 (7-7)
Manipur	Imphal West	88	4	98	791 (612-970)
Manipur	Jiribam	24		4	574 (477-671)
Manipur	Kakching	9	8	15	259 (243-275)
Manipur	Kamjong			40	2 (2-2)
Manipur	Kangpokpi	54		56	804 (715-892)
Manipur	Pherzawl			3	1 (1-1)
Manipur	Tamenglong			130	39 (39-39)
Manipur	Tengnoupal	26		34	852 (703-1001)
Manipur	Thoubal	21	20	59	598 (509-687)
Manipur	Ukhrul	13		48	120 (60-180)
Meghalaya	East Jaintia Hills	12		17	427 (392-462)
Meghalaya	East Khasi Hills	28	10		768 (588-949)
Meghalaya	Ri Bhoi	20			431 (347-514)
Meghalaya	South Garo Hills	15			487 (394-581)
Meghalaya	West Garo Hills	12			344 (294-395)
Meghalaya	West Jaintia Hills	25		29	753 (669-836)
Meghalaya	West Khasi Hills	4			86 (73-100)
Mizoram	Aizawl	57		40	634 (542-725)
Mizoram	Champhai	8		44	172 (153-190)
Mizoram	Kolasib	15		32	230 (213-247)
Mizoram	Lawngtlai	5		9	35 (31-39)
Mizoram	Lunglei	8		20	131 (119-142)
Mizoram	Mamit	9		49	129 (110-147)
Mizoram	Saiha	8			30 (24-36)
Mizoram	Serchhip	6		3	74 (63-84)
Maharashtra	Mumbai	580	46		18254 (16572-19935)
Nagaland	Mon	45			631 (525-736)
Nagaland	Dimapur	44	1		516 (361-670)

State	Distict	Number of Hot-Spots	Number of Network Operators	Villages	Estimates
Nagaland	Kiphire	8			39 (31-48)
Nagaland	Kohima	20			152 (114-190)
Nagaland	Mokokchung	51	14		458 (355-560)
Nagaland	Phek	12			55 (46-64)
Nagaland	Tuensang	15	3		266 (215-317)
Nagaland	Wokha	12			116 (108-123)
Nagaland	Zunheboto	4			14 (11-17)
Odisha	Anugul	30	2		581 (515-646)
Odisha	Balangir	110	1	100	648 (513-782)
Odisha	Baleshwar	118			1554 (1412-1696)
Odisha	Bargarh	12	1		176 (163-189)
Odisha	Bhadrak	67	7		975 (863-1087)
Odisha	Boudh	13			162 (147-176)
Odisha	Cuttack	85	13		701 (602-799)
Odisha	Deogarh	12			198 (185-210)
Odisha	Dhenkanal	61			605 (526-684)
Odisha	Gajapati	48			554 (506-601)
Odisha	Ganjam	60	10	211	872 (801-942)
Odisha	Jagatsinghapur	48			457 (309-605)
Odisha	Jajapur	103	2		1086 (969-1203)
Odisha	Jharsuguda	27			132 (99-164)
Odisha	Kalahandi	55	2	145	698 (648-747)
Odisha	Kandhamal	99	13		1139 (939-1338)
Odisha	Kendrapara	123			744 (619-870)
Odisha	Kendujhar	112	4		1347 (1096-1597)
Odisha	Khordha	91	31	183	1378 (1280-1475)
Odisha	Koraput	147	6		1537 (1267-1806)
Odisha	Malkangiri	94	2		852 (729-976)
Odisha	Mayurbhanj	142	4		1616 (1304-1928)

State	Distict	Number of Hot-Spots	Number of Network Operators	Villages	Estimates
Odisha	Nabarangpur	62	7		494 (426-562)
Odisha	Nayagarh	53	5		452 (424-480)
Odisha	Nuapada	63		208	1310 (1238-1381)
Odisha	Puri	106	70		1640 (1497-1782)
Odisha	Rayagada	44	2		522 (466-577)
Odisha	Sambalpur	12			151 (131-170)
Odisha	Sonepur	15			189 (167-210)
Odisha	Sundargarh	101	8	110	1857 (1686-2028)
Puducherry	Karaikal	22	4		567 (509-626)
Puducherry	Mahe	13			75 (58-92)
Puducherry	Pondicherry	48	9		1370 (1236-1503)
Puducherry	Yanam	18			502 (393-610)
Punjab	Amritsar	98	11	145	2990 (2604-3376)
Punjab	Barnala	68	13		1092 (988-1196)
Punjab	Bathinda	34	4		580 (473-687)
Punjab	Faridkot	24	5	60	298 (285-310)
Punjab	Fatehgarh Sahib	45	32		1186 (1066-1307)
Punjab	Firozepur	20	2	23	617 (591-643)
Punjab	Gurdaspur	56	16		1051 (933-1168)
Punjab	Hoshiarpur	86	17		1714 (1411-2017)
Punjab	Jalandhar	118	17		1720 (1446-1993)
Punjab	Kapurthala	45	12		829 (741-918)
Punjab	Ludhiana	67	5	105	1937 (1729-2146)
Punjab	Mansa	122	19		2085 (1868-2303)
Punjab	Moga	84	8	100	2704 (2548-2859)
Punjab	Nawanshahr	7	1		128 (112-143)
Punjab	Pathankot	39	10		794 (663-926)
Punjab	Patiala	111	49		2085 (1861-2308)
Punjab	Rupnagar	21	1		504 (457-551)

State	Distict	Number of Hot-Spots	Number of Network Operators	Villages	Estimates
Punjab	S.A.S Nagar	65	17		1537 (1371-1702)
Punjab	Sangrur	68	19		1045 (932-1158)
Punjab	Sri Muktsar Sahib	52	19	67	927 (863-991)
Punjab	Tarn Taran	66	8	96	1482 (1359-1605)
Rajasthan	Ajmer	34	7		481 (432-530)
Rajasthan	Alwar	34	4		955 (874-1037)
Rajasthan	Banswara	42	10		931 (844-1017)
Rajasthan	Barmer	9	2		290 (271-309)
Rajasthan	Bharatpur	37	4		727 (686-768)
Rajasthan	Bhilwara	64	9		567 (489-644)
Rajasthan	Bikaner	36	3		576 (528-625)
Rajasthan	Bundi	30	5		1018 (899-1136)
Rajasthan	Chittorgarh	34	4		479 (435-523)
Rajasthan	Churu	48	5		421 (327-514)
Rajasthan	Dholpur	43			290 (255-325)
Rajasthan	Dungarpur	41	9		807 (723-891)
Rajasthan	Ganganagar	51	9		1041 (941-1142)
Rajasthan	Hanumangarh	35	8		646 (578-714)
Rajasthan	Jaipur	54	29		1372 (1289-1454)
Rajasthan	Jaisalmer	27	7		777 (727-826)
Rajasthan	Jalore	30	4		240 (221-259)
Rajasthan	Jhalawar	14	14		483 (456-511)
Rajasthan	Jhunjhunu	52	13		1176 (1087-1265)
Rajasthan	Jodhpur	97	21		1487 (1368-1607)
Rajasthan	Karauli	14	7		372 (340-404)
Rajasthan	Kota	24			561 (523-598)
Rajasthan	Nagaur	41	5		1173 (1090-1256)
Rajasthan	Pali	49	8		707 (649-765)
Rajasthan	Pratapgarh	16	1		208 (191-225)

State	Distict	Number of Hot-Spots	Number of Network Operators	Villages	Estimates
Rajasthan	Rajsamand	46	11		538 (492-585)
Rajasthan	Sawai Madhopur	13	5		620 (573-666)
Rajasthan	Sikar	52	7		450 (385-515)
Rajasthan	Sirohi	32	3		574 (536-612)
Rajasthan	Tonk	23	12		465 (433-498)
Rajasthan	Udaipur	33	7		601 (552-651)
Sikkim	East District	54			456 (388-524)
Sikkim	South District	21			275 (246-304)
Tamil Nadu	Ariyalur	30	3		601 (538-664)
Tamil Nadu	Chennai	201	7		3554 (3094-4015)
Tamil Nadu	Coimbatore	143	25		1338 (1105-1571)
Tamil Nadu	Cuddalore	67	25		1588 (1428-1748)
Tamil Nadu	Dharmapuri	118	29	167	3710 (3467-3953)
Tamil Nadu	Dindigul	45	2	161	1984 (1880-2088)
Tamil Nadu	Erode	51	4	173	1703 (1587-1819)
Tamil Nadu	Kanchipuram	106	10		2714 (2418-3010)
Tamil Nadu	Kanniyakumari	53	7		1443 (1343-1543)
Tamil Nadu	Karur	54	4		1381 (1140-1623)
Tamil Nadu	Krishnagiri	145	77		3906 (3616-4197)
Tamil Nadu	Madurai	141	9		3485 (2807-4163)
Tamil Nadu	Nagapattinam	41			762 (618-907)
Tamil Nadu	Namakkal	139	14		1530 (1331-1729)
Tamil Nadu	Perambalur	55	1		834 (699-970)
Tamil Nadu	Pudukkottai	110	12		1090 (868-1311)
Tamil Nadu	Ramanathapuram	47	5	116	1658 (1545-1772)
Tamil Nadu	Salem	149	56	218	4327 (4084-4571)
Tamil Nadu	Sivaganga	45	2		751 (606-897)
Tamil Nadu	Thanjavur	18	1	149	1180 (1147-1214)
Tamil Nadu	The Nilgiris			140	252 (252-252)

State	Distict	Number of Hot-Spots	Number of Network Operators	Villages	Estimates
Tamil Nadu	Theni	37	7	115	1139 (1010-1267)
Tamil Nadu	Thiruvallur	81	11	132	2538 (2327-2749)
Tamil Nadu	Thiruvarur	10			233 (206-261)
Tamil Nadu	Tiruchirappalli	148	4		3509 (3072-3946)
Tamil Nadu	Tirunelveli	66	9	164	2319 (2150-2487)
Tamil Nadu	Tiruppur	30	3		386 (311-461)
Tamil Nadu	Tiruvannamalai	56	2	173	2403 (2341-2465)
Tamil Nadu	Tuticorin	85	6		1963 (1795-2131)
Tamil Nadu	Vellore	113	3	180	2957 (2631-3282)
Tamil Nadu	Villupuram	58	11	154	1995 (1925-2065)
Tamil Nadu	Virudhunagar	74	23		1541 (1294-1787)
Telangana	Adilabad	202	25	35	9081 (8095-10068)
Telangana	Bhadradri Kothagudem			44	558 (558-558)
Telangana	Hanumakonda			27	265 (265-265)
Telangana	Hyderabad	563	89		17941 (16205-19677)
Telangana	Jagitial			45	169 (169-169)
Telangana	Jangoan			32	226 (226-226)
Telangana	Jayashankar Bhoopalpally			20	133 (133-133)
Telangana	Jogulamba Gadwal			20	260 (260-260)
Telangana	Kamareddy		10	35	228 (228-228)
Telangana	Karimnagar	110	20	31	4480 (4258-4703)
Telangana	Khammam	119	101	95	6097 (5524-6669)
Telangana	Komaram Bheem Asifabad			11	53 (53-53)
Telangana	Mahabubabad			41	356 (356-356)
Telangana	Mahbubnagar	196	46	65	7862 (6908-8816)
Telangana	Mancherial			50	310 (310-310)
Telangana	Medak	123	25		2083 (1781-2384)
Telangana	Medchal-Malkajgiri		4	34	187 (187-187)
Telangana	Mulug			27	139 (139-139)

State	Distict	Number of Hot-Spots	Number of Network Operators	Villages	Estimates
Telangana	Nagarkurnool			67	501 (501-501)
Telangana	Nalgonda	79	72	63	7214 (6289-8140)
Telangana	Narayanpet		3	25	264 (264-264)
Telangana	Nirmal			47	265 (265-265)
Telangana	Nizamabad	79	31	84	4373 (3833-4913)
Telangana	Peddapalli			23	213 (213-213)
Telangana	Rajanna Sircilla			41	116 (116-116)
Telangana	Rangareddy	79	48	85	3914 (3620-4209)
Telangana	Suryapet		22	63	596 (596-596)
Telangana	Vikarabad		2	37	154 (154-154)
Telangana	Wanaparthy			36	278 (278-278)
Telangana	Warangal	74	51	57	6860 (6312-7409)
Telangana	Yadadri Bhuvanagiri		19	24	204 (204-204)
Tripura	Dhalai	77	4		650 (609-691)
Tripura	Gomati	81			693 (608-777)
Tripura	Khowai	50	4	41	474 (442-505)
Tripura	North Tripura	104	8	77	931 (881-982)
Tripura	Sepahijala	40	2	34	478 (452-504)
Tripura	South Tripura	222	5		1934 (1745-2124)
Tripura	Unakoti	45	7	67	413 (389-438)
Tripura	West Tripura	72	8	52	669 (625-712)
Uttar Pradesh	Agra	38	10		634 (569-698)
Uttar Pradesh	Aligarh	27	2		264 (246-282)
Uttar Pradesh	Allahabad	9	1		76 (62-90)
Uttar Pradesh	Ambedkar Nagar	48	9		969 (868-1070)
Uttar Pradesh	Amroha	57	5		578 (490-665)
Uttar Pradesh	Auraiya	49	5		546 (515-577)
Uttar Pradesh	Azamgarh	16	9		308 (281-335)
Uttar Pradesh	Bahraich	60	9		1469 (1341-1596)

State	Distict	Number of Hot-Spots	Number of Network Operators	Villages	Estimates
Uttar Pradesh	Ballia	33	4		426 (399-453)
Uttar Pradesh	Balrampur	10			71 (65-78)
Uttar Pradesh	Banda	28			447 (386-507)
Uttar Pradesh	Bareilly	81	4		542 (485-598)
Uttar Pradesh	Basti	13	6	220	246 (232-259)
Uttar Pradesh	Bhadohi	1			12 (9-14)
Uttar Pradesh	Budaun	14			302 (264-341)
Uttar Pradesh	Bulandshahr	25			359 (339-379)
Uttar Pradesh	Chandauli	30	3		422 (355-488)
Uttar Pradesh	Chitrakoot	8			79 (71-86)
Uttar Pradesh	Deoria	17	3		231 (221-241)
Uttar Pradesh	Etah	9	4		216 (211-221)
Uttar Pradesh	Etawah	34	6		472 (452-493)
Uttar Pradesh	Faizabad	28	4		561 (518-605)
Uttar Pradesh	Farrukhabad	20			244 (220-268)
Uttar Pradesh	Fatehpur	29	10		659 (592-726)
Uttar Pradesh	Firozabad	18	2		285 (255-315)
Uttar Pradesh	Gautam Buddha Nagar	20	9		512 (484-540)
Uttar Pradesh	Ghaziabad	41	19		595 (528-662)
Uttar Pradesh	Ghazipur	35	7		605 (547-664)
Uttar Pradesh	Gonda	28	3		370 (343-397)
Uttar Pradesh	Gorakhpur	33	3	220	384 (358-410)
Uttar Pradesh	Hamirpur	23	4		500 (468-531)
Uttar Pradesh	Hapur	28	2		337 (308-366)
Uttar Pradesh	Hardoi	27	4		822 (748-895)
Uttar Pradesh	Hathras	21	3		235 (222-248)
Uttar Pradesh	Jalaun	16	2		347 (321-372)
Uttar Pradesh	Jaunpur	63	2	180	1335 (1262-1408)
Uttar Pradesh	Jhansi	23	11		477 (457-498)

State	Distict	Number of Hot-Spots	Number of Network Operators	Villages	Estimates
Uttar Pradesh	Kannauj	15	2		235 (218-252)
Uttar Pradesh	Kanpur Dehat	19	3		256 (237-275)
Uttar Pradesh	Kanpur Nagar	99	35		1266 (1128-1404)
Uttar Pradesh	Kasganj	37	7		448 (419-477)
Uttar Pradesh	Kaushambi	35	1		478 (419-538)
Uttar Pradesh	Kheri	81	7		672 (618-726)
Uttar Pradesh	Kushi Nagar	96	4	95	1093 (1028-1157)
Uttar Pradesh	Lalitpur	45	2		646 (601-691)
Uttar Pradesh	Lucknow	86	32	180	3525 (3285-3766)
Uttar Pradesh	Maharajganj	39	7		570 (535-605)
Uttar Pradesh	Mahoba	22	8		379 (332-425)
Uttar Pradesh	Mainpuri	38	1		449 (412-485)
Uttar Pradesh	Mathura	20			285 (251-319)
Uttar Pradesh	Mau	24	2		371 (321-421)
Uttar Pradesh	Meerut	41	13		396 (323-469)
Uttar Pradesh	Mirzapur	2			8 (7-9)
Uttar Pradesh	Moradabad	80	8	136	1431 (1337-1525)
Uttar Pradesh	Muzaffarnagar	44	5		501 (469-533)
Uttar Pradesh	Pilibhit	34	3		928 (859-998)
Uttar Pradesh	Pratapgarh	18			418 (384-453)
Uttar Pradesh	Rae Bareli	73	8		1432 (1334-1530)
Uttar Pradesh	Rampur	56	1		405 (368-443)
Uttar Pradesh	Saharanpur	42	11		541 (493-589)
Uttar Pradesh	Sambhal	5			67 (61-74)
Uttar Pradesh	Sant Kabeer Nagar	23	1		430 (402-458)
Uttar Pradesh	Shahjahanpur	51	5		577 (543-611)
Uttar Pradesh	Shamli	8	2		56 (51-61)
Uttar Pradesh	Siddharth Nagar	16	11	140	713 (672-755)
Uttar Pradesh	Sitapur	36	11		891 (828-954)

State	Distict	Number of Hot-Spots	Number of Network Operators	Villages	Estimates
Uttar Pradesh	Sonbhadra	10	5		366 (291-441)
Uttar Pradesh	Sultanpur	27	1		760 (697-822)
Uttar Pradesh	Unnao	37	5		1652 (1583-1720)
Uttar Pradesh	Varanasi	93	18		1299 (1077-1521)
Uttarakhand	Almora	25	4		411 (336-486)
Uttarakhand	Champawat	26			348 (263-434)
Uttarakhand	Dehradun	59	23		1754 (1588-1919)
Uttarakhand	Haridwar	59	10		847 (796-899)
Uttarakhand	Nainital	47	13		872 (761-983)
Uttarakhand	Pauri Garhwal	48			479 (442-517)
Uttarakhand	Pithoragarh	44	3		793 (666-919)
Uttarakhand	Rudra Prayag	9			77 (69-86)
Uttarakhand	Tehri Garhwal	20			80 (67-93)
Uttarakhand	Udam Singh Nagar	77	9		1521 (1371-1670)
Uttarakhand	Uttar Kashi	7			31 (28-35)
West Bengal	24 Paraganas North	43	5	7	958 (880-1035)
West Bengal	24 Paraganas South	22	4	93	574 (528-621)
West Bengal	Alipurduar	4		35	154 (152-156)
West Bengal	Basirhat	35		5	834 (769-899)
West Bengal	Birbhum	12	3	29	505 (490-520)
West Bengal	Bishnupur_Bankura	6			183 (163-202)
West Bengal	Coochbehar	17			671 (619-723)
West Bengal	Darjeeling	31		21	572 (502-643)
West Bengal	Diamond Harbour	14		33	515 (483-548)
West Bengal	Dinajpur Dakshin	8	8		147 (136-157)
West Bengal	Dinajpur Uttar	17	5	116	879 (855-903)
West Bengal	Hooghly	39	9	11	946 (852-1039)
West Bengal	Howrah	20	2		695 (639-751)
West Bengal	Jalpaiguri	9	1	53	424 (401-447)

State	Distict	Number of Hot-Spots	Number of Network Operators	Villages	Estimates
West Bengal	Kalimpong		2		4 (4-4)
West Bengal	Kolkata	264	5		7210 (6605-7816)
West Bengal	Maldah	12			251 (234-268)
West Bengal	Medinipur East	8	1		161 (137-184)
West Bengal	Medinipur West	2		90	194 (190-197)
West Bengal	Murshidabad	14		100	877 (816-938)
West Bengal	Nadia	18	1		330 (305-355)
West Bengal	Nandigram	14	12		482 (443-521)
West Bengal	Paschim Bardhaman	62		32	1827 (1718-1937)
West Bengal	Purba Bardhaman	32	6	123	890 (853-928)
West Bengal	Rampurhat	4	4	55	168 (161-175)
