# Supplementary material for: Programmatic mapping and population size estimation of key population in India: Method and findings
Source: PLOS Glob Public Health. 2025 May 7;5(5):e0004475. doi: 10.1371/journal.pgph.0004475 (PMC12057993; doi:10.1371/journal.pgph.0004475)
Supplement: S3 Table — (PDF) [file pgph.0004475.s009.pdf]

Supplementary Table S3. State/UT-wise size estimates of MSM (At hotspots, exclusively with network operators, exclusively in LWS villages) and adult men

State/UT	MSM Size Estimates			Adult Men Size
	At hotspots	Exclusively with network operators	Exclusively in LWS villages	
Andhra Pradesh	21083.83	307	669	14892000
Arunachal Pradesh	900.5	0	0	426895
Assam	16126.5	540	0	9911002
Bihar	1857.167	145	374	32930001
Chandigarh	2428	141	0	397081
Chhattisgarh	2813.833	0	75	8059000
Delhi	24442	2584	0	6501001
Goa	3331.167	8	0	454140
Gujarat	29989.17	0	4310	20418002
Haryana	7761.833	260	0	8880001
Himachal Pradesh	1242	10	0	2102000
Jammu And Kashmir	586.5	90	0	4078000
Jharkhand	1742.167	0	0	10676000
Karnataka	42647.83	1925	1058	19139001
Kerala	13653	183	0	8684001
Madhya Pradesh	16853.67	1132	99	23585000
Maharashtra	30846.33	8738	603	36896000
Manipur	1408.667	0	98	865449
Meghalaya	321.1667	21	1	827274
Mizoram	739.1667	0	102	332204
Nagaland	868.5	370	0	614887
Odisha	5285.333	101	591	12499001
Puducherry	2309.167	180	0	429251
Punjab	7431.167	408	206	9115001
Rajasthan	6948.667	402	0	22125000
Sikkim	0	0	0	211982
Tamil Nadu	33361.83	3274	1648	20806000
Telangana	14704.33	1238	485	10927000
Tripura	796.3333	33	47	1169296
Uttar Pradesh	18383.83	1182	103	65323000
Uttarakhand	2875.333	8	0	3314000
West Bengal	3003	454	75	28268001
India	316742	23734	10544	384856471
