# Supplementary material for: Programmatic mapping and population size estimation of key population in India: Method and findings
Source: PLOS Glob Public Health. 2025 May 7;5(5):e0004475. doi: 10.1371/journal.pgph.0004475 (PMC12057993; doi:10.1371/journal.pgph.0004475)
Supplement: S4 Table — (PDF) [file pgph.0004475.s010.pdf]

Supplementary Table S2. District-wise size estimates of MSM, PMPSE 2020-22

State	Distict	Number of Hot-Spots	Number of Network Operators	Villages	Estimates
**Andhra Pradesh**	**Anantapur**	33	1		379 (327-432)
**Andhra Pradesh**	**Annamayya**	57	1	3	873 (728-1019)
**Andhra Pradesh**	**Bapatla**	9	1		122 (105-138)
**Andhra Pradesh**	**Chittoor**	33			227 (195-259)
**Andhra Pradesh**	**East Godavari**	2			152 (125-178)
**Andhra Pradesh**	**Eluru**	4		41	140 (129-151)
**Andhra Pradesh**	**Guntur**	24		55	723 (649-796)
**Andhra Pradesh**	**Kakinada**	27	2	5	432 (359-505)
**Andhra Pradesh**	**Konaseema**	18			681 (571-791)
**Andhra Pradesh**	**Krishna**	82	8	46	2877 (2531-3223)
**Andhra Pradesh**	**Kurnool**	183		121	5992 (5095-6888)
**Andhra Pradesh**	**Nandyal**	86	17		3793 (3121-4464)
**Andhra Pradesh**	**Palnadu**	34			996 (851-1141)
**Andhra Pradesh**	**Prakasam**	10	1	13	357 (317-398)
**Andhra Pradesh**	**Spsr Nellore**	22		9	519 (448-589)
**Andhra Pradesh**	**Sri Satya Sai**	15	4		289 (255-322)
**Andhra Pradesh**	**Srikakulam**	34		20	617 (481-753)
**Andhra Pradesh**	**Visakhapatanam**	88		1	1204 (1026-1382)
**Andhra Pradesh**	**Vizianagaram**	33	3	3	460 (371-549)
**Andhra Pradesh**	**West Godavari**	20	1		476 (358-594)
**Andhra Pradesh**	**Y.S.R.**	42		53	752 (522-981)
**Arunachal Pradesh**	**East Siang**	18			219 (204-234)
**Arunachal Pradesh**	**Lower Subansiri**	1			4 (3-4)
**Arunachal Pradesh**	**Namsai**	5			24 (19-28)
**Arunachal Pradesh**	**Papum Pare**	95			577 (511-642)
**Arunachal Pradesh**	**West Siang**	6			78 (72-83)
**Assam**	**Barpeta**	88			1537 (1371-1704)

State	Distict	Number of Hot-Spots	Number of Network Operators	Villages	Estimates
Assam	Bongaigaon	1			9 (8-10)
Assam	Cachar	31	1		434 (371-496)
Assam	Darrang	2			28 (25-30)
Assam	Dhemaji	7			78 (67-89)
Assam	Dhubri	8	1		100 (89-111)
Assam	Dibrugarh	145			2078 (1572-2583)
Assam	East Karbi Anglong	9	6		136 (116-155)
Assam	Goalpara	35	1		499 (432-565)
Assam	Golaghat	45	5		432 (384-480)
Assam	Hailakandi	4			74 (65-82)
Assam	Jorhat	15	11		345 (311-379)
Assam	Kamrup	18			510 (365-654)
Assam	Kamrup Metro	227	49		3300 (2857-3742)
Assam	Lakhimpur	14			187 (165-210)
Assam	Majuli	51			396 (322-470)
Assam	Marigaon	8			150 (139-161)
Assam	Nagaon	9	7		292 (279-304)
Assam	Nalbari	42	2		3258 (3124-3392)
Assam	Sivasagar	50			2000 (1795-2204)
Assam	Sonitpur	33	6		635 (517-753)
Assam	Tinsukia	16			193 (171-215)
Bihar	Begusarai	12		7	248 (231-265)
Bihar	Bhojpur	10	2		250 (237-263)
Bihar	Buxar	5	4		138 (133-143)
Bihar	Darbhanga	1		29	46 (46-46)
Bihar	Kaimur (Bhabua)	1			12 (9-15)
Bihar	Lakhisarai	8			103 (91-114)
Bihar	Nalanda	5	1		93 (86-100)
Bihar	Patna	21	2		561 (523-599)

State	Distict	Number of Hot-Spots	Number of Network Operators	Villages	Estimates
**Bihar**	**Purbi Champaran**	3			49 (44-54)
**Bihar**	**Purnia**	1			21 (19-23)
**Bihar**	**Rohtas**	1	3		39 (37-42)
**Bihar**	**Saran**	4		86	181 (180-181)
**Bihar**	**Sheikhpura**	3			54 (50-57)
**Bihar**	**Sheohar**	7			120 (112-128)
**Bihar**	**Sitamarhi**			93	144 (144-144)
**Bihar**	**Siwan**	10	1		113 (103-123)
**Bihar**	**Vaishali**	9	2		206 (192-219)
**Chandigarh**	**Chandigarh**	106	16		2569 (2306-2832)
**Chhattisgarh**	**Balod**	8			43 (38-48)
**Chhattisgarh**	**Baloda Bazar**	5			73 (63-82)
**Chhattisgarh**	**Bastar**	26			325 (281-369)
**Chhattisgarh**	**Bemetara**	5			37 (33-40)
**Chhattisgarh**	**Bilaspur**	24	5	40	368 (327-410)
**Chhattisgarh**	**Dhamtari**	2			36 (34-38)
**Chhattisgarh**	**Durg**	31		26	442 (414-471)
**Chhattisgarh**	**Gaurela-Pendra-Marwahi**	4			66 (59-72)
**Chhattisgarh**	**Janjgir-Champa**	3			51 (46-56)
**Chhattisgarh**	**Kanker**	4			58 (53-62)
**Chhattisgarh**	**Korba**	14			204 (178-230)
**Chhattisgarh**	**Korea**	5			98 (93-102)
**Chhattisgarh**	**Raigarh**	3			69 (65-73)
**Chhattisgarh**	**Raipur**	31		17	628 (589-667)
**Chhattisgarh**	**Rajnandgaon**	14	3	16	256 (237-274)
**Chhattisgarh**	**Surajpur**	5			71 (64-78)
**Chhattisgarh**	**Surguja**	5			66 (57-75)
**Delhi**	**Central**	115	7		4023 (3746-4299)
**Delhi**	**East**	79	14		2526 (2338-2714)

State	Distict	Number of Hot-Spots	Number of Network Operators	Villages	Estimates
**Delhi**	**New Delhi**	45			1143 (1026-1260)
**Delhi**	**North**	60	12		2307 (2219-2396)
**Delhi**	**North East**	36			1156 (1041-1271)
**Delhi**	**North West**	150	10		4416 (4047-4785)
**Delhi**	**Shahdara**	34	1		1019 (929-1109)
**Delhi**	**South**	107	33		2506 (2378-2633)
**Delhi**	**South East**	92	19		2061 (1857-2265)
**Delhi**	**South West**	59			2304 (2131-2478)
**Delhi**	**West**	74	14		3565 (3272-3858)
**Goa**	**North Goa**	70	2		1947 (1537-2357)
**Goa**	**South Goa**	52			1392 (1250-1534)
**Gujarat**	**Ahmadabad**	204		83	1885 (1476-2294)
**Gujarat**	**Amreli**	61		98	1152 (1070-1234)
**Gujarat**	**Anand**	70		58	1450 (1247-1653)
**Gujarat**	**Arvalli**	24		46	416 (378-454)
**Gujarat**	**Banas Kantha**	28		123	871 (791-950)
**Gujarat**	**Bharuch**	21			380 (318-441)
**Gujarat**	**Bhavnagar**	174		87	5209 (4742-5676)
**Gujarat**	**Botad**	1		12	110 (106-113)
**Gujarat**	**Chhotaudepur**	32		35	263 (206-320)
**Gujarat**	**Devbhumi Dwarka**	10			591 (567-614)
**Gujarat**	**Dohad**	28			519 (428-611)
**Gujarat**	**Gandhinagar**	5		54	295 (279-311)
**Gujarat**	**Gir Somnath**	29			487 (437-537)
**Gujarat**	**Jamnagar**	49			741 (682-800)
**Gujarat**	**Junagadh**	25			317 (292-341)
**Gujarat**	**Kachchh**	45		116	1312 (1217-1406)
**Gujarat**	**Kheda**	28		67	797 (767-826)
**Gujarat**	**Mahesana**	38		117	1077 (1012-1141)

State	Distict	Number of Hot-Spots	Number of Network Operators	Villages	Estimates
Gujarat	Mahisagar	3			27 (22-32)
Gujarat	Morbi	13			338 (309-366)
Gujarat	Narmada	3			103 (93-112)
Gujarat	Navsari	26			285 (229-342)
Gujarat	Panch Mahals	22			256 (214-297)
Gujarat	Patan	83			923 (751-1094)
Gujarat	Porbandar	20			437 (382-492)
Gujarat	Rajkot	51			1500 (1387-1612)
Gujarat	Sabar Kantha	22		53	655 (612-698)
Gujarat	Surat	217			7068 (6340-7796)
Gujarat	Surendranagar	34			622 (566-677)
Gujarat	Tapi	7			140 (133-148)
Gujarat	Vadodara	86		46	3237 (2940-3534)
Gujarat	Valsad	46			840 (667-1013)
Haryana	Ambala	74	4		348 (302-393)
Haryana	Faridabad	80			1508 (1386-1631)
Haryana	Fatehabad	8			45 (39-50)
Haryana	Gurugram	42			956 (867-1045)
Haryana	Hisar	14			84 (73-95)
Haryana	Jhajjar	13	1		82 (72-93)
Haryana	Jind	56			578 (471-685)
Haryana	Kaithal	79	4		928 (807-1048)
Haryana	Karnal	20			349 (314-384)
Haryana	Kurukshetra	12	4		317 (298-337)
Haryana	Mewat	1			11 (9-13)
Haryana	Palwal	13			221 (195-247)
Haryana	Panchkula	4	1		20 (18-21)
Haryana	Panipat	70			488 (432-544)
Haryana	Rewari	1			6 (5-6)

State	Distict	Number of Hot-Spots	Number of Network Operators	Villages	Estimates
Haryana	Rohtak	59			415 (373-456)
Haryana	Sirsa	74	28		842 (783-900)
Haryana	Sonipat	24			214 (202-226)
Haryana	Yamunanagar	94			611 (559-664)
Himachal Pradesh	Bilaspur_HP	2			17 (15-20)
Himachal Pradesh	Chamba	10	1		71 (64-79)
Himachal Pradesh	Hamirpur	39	1		327 (295-359)
Himachal Pradesh	Kangra	16			145 (130-160)
Himachal Pradesh	Kullu	7	2		75 (68-83)
Himachal Pradesh	Mandi	9			39 (34-44)
Himachal Pradesh	Shimla	40			318 (245-391)
Himachal Pradesh	Sirmaur	6			109 (99-120)
Himachal Pradesh	Solan	9			34 (26-42)
Himachal Pradesh	Una	15			116 (107-125)
Jammu And Kashmir	Badgam	7			44 (32-57)
Jammu And Kashmir	Bandipora	1			9 (4-14)
Jammu And Kashmir	Baramulla	2			30 (18-41)
Jammu And Kashmir	Ganderbal	5			20 (11-28)
Jammu And Kashmir	Jammu	22	4		228 (189-267)
Jammu And Kashmir	Kupwara	2			15 (9-22)
Jammu And Kashmir	Srinagar	19	6		322 (279-366)
Jammu And Kashmir	Udhampur	1			8 (6-10)
Jharkhand	Bokaro	21			203 (182-223)
Jharkhand	Dhanbad	63			591 (479-702)
Jharkhand	East Singhbum	9			155 (136-174)
Jharkhand	Giridih	24			214 (193-234)
Jharkhand	Hazaribagh	8			55 (42-68)
Jharkhand	Ranchi	3			48 (41-55)
Jharkhand	West Singhbhum	29			477 (420-534)

State	Distict	Number of Hot-Spots	Number of Network Operators	Villages	Estimates
**Karnataka**	**Bagalkot**	43	14	46	1525 (1448-1602)
**Karnataka**	**Ballari**	400	42	33	3275 (3060-3490)
**Karnataka**	**Belagavi**	147	10	90	2817 (2556-3078)
**Karnataka**	**Bengaluru Rural**	60			645 (556-734)
**Karnataka**	**Bengaluru Urban**	173	1		4232 (3859-4605)
**Karnataka**	**Bidar**	37	18		1117 (1048-1186)
**Karnataka**	**Chamarajanagar**	149	3	113	2291 (2144-2439)
**Karnataka**	**Chikballapur**	96		81	2209 (1785-2633)
**Karnataka**	**Chikkamagaluru**	35	12		567 (482-652)
**Karnataka**	**Chitradurga**	49	5		741 (671-812)
**Karnataka**	**Dakshin Kannad**	73	15		1574 (1456-1692)
**Karnataka**	**Davangere**	41	7		995 (932-1057)
**Karnataka**	**Dharwad**	59			1980 (1912-2048)
**Karnataka**	**Gadag**	21		127	1355 (1288-1422)
**Karnataka**	**Hassan**	39		34	441 (375-506)
**Karnataka**	**Haveri**	49	1		1074 (962-1185)
**Karnataka**	**Kalaburagi**	53	12		2145 (1953-2338)
**Karnataka**	**Kodagu**	28			300 (277-322)
**Karnataka**	**Kolar**	77			1613 (1501-1724)
**Karnataka**	**Koppal**	70	12		847 (763-930)
**Karnataka**	**Mandya**	51	1	4	822 (691-953)
**Karnataka**	**Mysuru**	203			1894 (1520-2267)
**Karnataka**	**Raichur**	95	5		2427 (2255-2600)
**Karnataka**	**Ramanagara**	39	13		758 (707-810)
**Karnataka**	**Shivamogga**	44			576 (471-680)
**Karnataka**	**Tumakuru**	462			2683 (2327-3038)
**Karnataka**	**Udupi**	25			448 (381-514)
**Karnataka**	**Uttar Kannad**	45			308 (272-345)
**Karnataka**	**Vijayapura**	101		55	3451 (3299-3603)

State	Distict	Number of Hot-Spots	Number of Network Operators	Villages	Estimates
**Karnataka**	**Yadgir**	46	27		521 (444-597)
**Kerala**	**Alappuzha**	85	3		960 (839-1080)
**Kerala**	**Ernakulam**	51	1		619 (468-769)
**Kerala**	**Kannur**	71	5		1312 (1183-1441)
**Kerala**	**Kasaragod**	108			1067 (890-1244)
**Kerala**	**Kollam**	60			1060 (917-1204)
**Kerala**	**Kottayam**	42	2		1127 (1000-1254)
**Kerala**	**Kozhikode**	73	8		1592 (1138-2046)
**Kerala**	**Malappuram**	127	14		1785 (1488-2081)
**Kerala**	**Palakkad**	74			698 (581-815)
**Kerala**	**Pathanamthitta**	33			802 (693-911)
**Kerala**	**Thiruvananthapuram**	60	21		1433 (1186-1679)
**Kerala**	**Thrissur**	75			1311 (1200-1422)
**Kerala**	**Wayanad**	4	1		70 (64-75)
**Madhya Pradesh**	**Agar Malwa**	12	2		146 (127-166)
**Madhya Pradesh**	**Alirajpur**	15			178 (152-204)
**Madhya Pradesh**	**Anuppur**	1			3 (2-4)
**Madhya Pradesh**	**Balaghat**	9	3		207 (194-219)
**Madhya Pradesh**	**Barwani**	24		48	202 (181-223)
**Madhya Pradesh**	**Betul**	12	6		375 (353-397)
**Madhya Pradesh**	**Bhind**	9	3	24	69 (65-73)
**Madhya Pradesh**	**Bhopal**	45	3		993 (888-1098)
**Madhya Pradesh**	**Burhanpur**	6			149 (136-161)
**Madhya Pradesh**	**Chhatarpur**	23			365 (305-425)
**Madhya Pradesh**	**Chhindwara**	43	3	1	564 (465-663)
**Madhya Pradesh**	**Damoh**	5			24 (13-35)
**Madhya Pradesh**	**Datia**	26	3		248 (195-301)
**Madhya Pradesh**	**Dewas**	15	2		271 (253-289)
**Madhya Pradesh**	**Dhar**	29	6		410 (354-465)

State	Distict	Number of Hot-Spots	Number of Network Operators	Villages	Estimates
**Madhya Pradesh**	**East Nimar**	8	1		156 (133-179)
**Madhya Pradesh**	**Guna**	1			5 (4-6)
**Madhya Pradesh**	**Gwalior**	60	2		1221 (957-1485)
**Madhya Pradesh**	**Harda**	11	1		204 (182-227)
**Madhya Pradesh**	**Hoshangabad**	56	17		1162 (1075-1250)
**Madhya Pradesh**	**Indore**	83	21		1862 (1621-2104)
**Madhya Pradesh**	**Jabalpur**	65	24		1152 (991-1313)
**Madhya Pradesh**	**Jhabua**	6	2		195 (176-215)
**Madhya Pradesh**	**Katni**	19			156 (140-171)
**Madhya Pradesh**	**Khargone**	14			249 (227-271)
**Madhya Pradesh**	**Mandsaur**	17	2		371 (331-410)
**Madhya Pradesh**	**Morena**	27	6		619 (504-734)
**Madhya Pradesh**	**Neemuch**	1			15 (10-20)
**Madhya Pradesh**	**Panna**	19			308 (266-350)
**Madhya Pradesh**	**Raisen**	59	16		676 (633-719)
**Madhya Pradesh**	**Rajgarh**	2			21 (19-23)
**Madhya Pradesh**	**Ratlam**	20	5		338 (296-379)
**Madhya Pradesh**	**Rewa**	30			630 (546-713)
**Madhya Pradesh**	**Sagar**	104	1		1001 (843-1158)
**Madhya Pradesh**	**Satna**	17	2		297 (245-350)
**Madhya Pradesh**	**Sehore**	6	1		86 (76-95)
**Madhya Pradesh**	**Seoni**	4	1		79 (70-88)
**Madhya Pradesh**	**Shahdol**	3	1		35 (27-42)
**Madhya Pradesh**	**Shajapur**	8	2		166 (140-193)
**Madhya Pradesh**	**Sheopur**	1			3 (1-5)
**Madhya Pradesh**	**Shivpuri**	12	6		406 (357-454)
**Madhya Pradesh**	**Sidhi**	12	1		51 (37-64)
**Madhya Pradesh**	**Singrauli**	7	1		69 (57-81)
**Madhya Pradesh**	**Tikamgarh**	90	6	1	542 (489-595)

State	Distict	Number of Hot-Spots	Number of Network Operators	Villages	Estimates
**Madhya Pradesh**	**Ujjain**	38	1	35	716 (637-794)
**Madhya Pradesh**	**Umaria**	1			12 (11-13)
**Madhya Pradesh**	**Vidisha**	113	10		1081 (995-1167)
**Maharashtra**	**Ahmednagar**	42	6		1434 (1342-1525)
**Maharashtra**	**Akola**	58		45	841 (786-895)
**Maharashtra**	**Amravati**	45	152		4852 (4694-5010)
**Maharashtra**	**Aurangabad**	24	6	1	1215 (1158-1271)
**Maharashtra**	**Beed**	7			88 (76-100)
**Maharashtra**	**Buldhana**	4		17	106 (98-113)
**Maharashtra**	**Chandrapur**	31	68	12	2109 (2025-2193)
**Maharashtra**	**Dhule**	34	1	65	478 (449-507)
**Maharashtra**	**Gondia**	2			19 (16-22)
**Maharashtra**	**Hingoli**	9			88 (81-96)
**Maharashtra**	**Jalgaon**	66		14	792 (730-854)
**Maharashtra**	**Jalna**	4	6	14	261 (261-261)
**Maharashtra**	**Kolhapur**	40		20	643 (526-761)
**Maharashtra**	**Latur**	42	29		963 (898-1027)
**Maharashtra**	**Nagpur**	99		42	5915 (5399-6431)
**Maharashtra**	**Nanded**	75	76	79	1703 (1616-1790)
**Maharashtra**	**Nandurbar**	56			383 (335-431)
**Maharashtra**	**Nashik**	51		31	788 (659-918)
**Maharashtra**	**Osmanabad**	35	11		436 (387-485)
**Maharashtra**	**Parbhani**	30		26	748 (689-807)
**Maharashtra**	**Pune**	70		37	1267 (1031-1503)
**Maharashtra**	**Raigad**	11			151 (128-173)
**Maharashtra**	**Ratnagiri**	20			418 (353-483)
**Maharashtra**	**Sangli**	49		21	388 (314-462)
**Maharashtra**	**Satara**	23	1	18	278 (256-301)
**Maharashtra**	**Sindhudurg**	41	4		148 (130-165)

State	Distict	Number of Hot-Spots	Number of Network Operators	Villages	Estimates
Maharashtra	Solapur	50			1195 (1049-1340)
Maharashtra	Thane	71	5		3870 (3634-4106)
Maharashtra	Wardha			3	3 (3-3)
Maharashtra	Washim			24	33 (33-33)
Maharashtra	Yavatmal	100			1535 (1402-1669)
Manipur	Bishnupur	17		53	196 (169-223)
Manipur	Chandel			2	2 (2-2)
Manipur	Churachandpur	6			146 (109-182)
Manipur	Imphal East	1		31	29 (27-32)
Manipur	Imphal West	50		69	351 (279-422)
Manipur	Kakching	9		3	260 (224-296)
Manipur	Kangpokpi	10		2	117 (102-131)
Manipur	Tamenglong			23	2 (2-2)
Manipur	Tengnoupal			8	13 (13-13)
Manipur	Thoubal	14		18	391 (331-451)
Meghalaya	East Khasi Hills	16	4		343 (251-434)
Meghalaya	West Jaintia Hills			1	1 (1-1)
Mizoram	Aizawl	77			661 (549-772)
Mizoram	Champhai			16	40 (40-40)
Mizoram	Kolasib			20	60 (60-60)
Mizoram	Lunglei	6		1	80 (67-92)
Mizoram	Mamit			17	1 (1-1)
Maharashtra	Mumbai	83	18		7040 (6703-7377)
Nagaland	Dimapur	28			247 (198-296)
Nagaland	Kohima	34			253 (189-317)
Nagaland	Mokokchung	30	1		666 (596-736)
Nagaland	Tuensang	10			73 (69-77)
Odisha	Anugul	7			43 (35-50)
Odisha	Balangir	64		13	409 (350-468)

State	Distict	Number of Hot-Spots	Number of Network Operators	Villages	Estimates
**Odisha**	**Baleshwar**	22			256 (236-276)
**Odisha**	**Bargarh**	2			30 (28-32)
**Odisha**	**Bhadrak**	37	2		506 (436-576)
**Odisha**	**Boudh**	2			39 (37-40)
**Odisha**	**Cuttack**	16			108 (94-122)
**Odisha**	**Deogarh**	7			107 (100-113)
**Odisha**	**Dhenkanal**	29			157 (130-183)
**Odisha**	**Gajapati**	19	1		142 (125-160)
**Odisha**	**Ganjam**	35	2	206	413 (382-445)
**Odisha**	**Jagatsinghapur**	10			100 (66-134)
**Odisha**	**Jajapur**	15	1		130 (117-143)
**Odisha**	**Jharsuguda**	1			2 (1-2)
**Odisha**	**Kalahandi**	74	2	99	692 (618-766)
**Odisha**	**Kendujhar**	39			328 (254-403)
**Odisha**	**Khordha**	26	4	73	439 (413-465)
**Odisha**	**Koraput**	27			161 (137-185)
**Odisha**	**Malkangiri**	5			32 (26-37)
**Odisha**	**Mayurbhanj**	27			298 (267-329)
**Odisha**	**Nabarangpur**	8			44 (40-48)
**Odisha**	**Nayagarh**	25	3		264 (243-285)
**Odisha**	**Nuapada**	25		67	332 (299-366)
**Odisha**	**Puri**	33	8		242 (208-275)
**Odisha**	**Rayagada**	19			184 (162-207)
**Odisha**	**Sambalpur**	3			19 (15-22)
**Odisha**	**Sonepur**	17			217 (192-242)
**Odisha**	**Sundargarh**	24	1		285 (250-319)
**Puducherry**	**Karaikal**	20			485 (432-539)
**Puducherry**	**Mahe**	11			126 (111-141)
**Puducherry**	**Pondicherry**	59	4		1452 (1294-1610)

State	Distict	Number of Hot-Spots	Number of Network Operators	Villages	Estimates
Puducherry	Yanam	8			425 (342-508)
Punjab	Amritsar	36	1	43	644 (562-725)
Punjab	Barnala	28	2		345 (303-387)
Punjab	Bathinda	5			93 (76-110)
Punjab	Faridkot	10		35	59 (55-63)
Punjab	Fatehgarh Sahib	9			103 (85-121)
Punjab	Firozepur	2		9	75 (72-78)
Punjab	Gurdaspur	28	6		515 (441-590)
Punjab	Hoshiarpur	85	30		1869 (1596-2142)
Punjab	Jalandhar	46			491 (436-546)
Punjab	Kapurthala	25			499 (418-580)
Punjab	Ludhiana	29		46	411 (372-450)
Punjab	Mansa	74	2		565 (494-635)
Punjab	Moga	9		7	66 (57-75)
Punjab	Nawanshahr	12	2		264 (238-290)
Punjab	Pathankot	31	4		359 (294-423)
Punjab	Patiala	24	4		474 (425-524)
Punjab	Rupnagar	7			174 (162-186)
Punjab	S.A.S Nagar	21	5		362 (313-411)
Punjab	Sangrur	13	2		157 (129-186)
Punjab	Sri Muktsar Sahib	18	5	27	254 (238-270)
Punjab	Tarn Taran	30		71	266 (248-283)
Rajasthan	Ajmer	38	4		473 (408-538)
Rajasthan	Alwar	11	3		265 (245-285)
Rajasthan	Banswara	13	3		318 (283-352)
Rajasthan	Barmer	1	2		48 (44-53)
Rajasthan	Bharatpur	15	1		394 (351-437)
Rajasthan	Bhilwara	47	4		397 (336-458)
Rajasthan	Bikaner	15	2		126 (112-140)

State	Distict	Number of Hot-Spots	Number of Network Operators	Villages	Estimates
Rajasthan	Bundi	7	1		215 (198-233)
Rajasthan	Chittorgarh	22	1		255 (237-274)
Rajasthan	Churu	43	4		230 (169-291)
Rajasthan	Dholpur	13			127 (100-153)
Rajasthan	Dungarpur	18	2		347 (313-380)
Rajasthan	Ganganagar	28	4		344 (292-396)
Rajasthan	Hanumangarh	39	3		398 (340-455)
Rajasthan	Jaipur	56	9		486 (420-551)
Rajasthan	Jaisalmer	9			164 (154-173)
Rajasthan	Jalore	4	1		13 (11-14)
Rajasthan	Jhalawar	2			17 (15-19)
Rajasthan	Jhunjhunu	23	4		375 (341-408)
Rajasthan	Jodhpur	14	1		392 (341-443)
Rajasthan	Karauli	7	1		37 (34-41)
Rajasthan	Kota	21			515 (481-548)
Rajasthan	Nagaur	7			50 (43-57)
Rajasthan	Pali	2	1		14 (13-14)
Rajasthan	Pratapgarh	12	1		113 (103-122)
Rajasthan	Rajsamand	1	1		2 (2-3)
Rajasthan	Sawai Madhopur	7	2		275 (259-290)
Rajasthan	Sikar	47	2		290 (244-336)
Rajasthan	Sirohi	1			7 (6-8)
Rajasthan	Tonk	22	4		217 (196-237)
Rajasthan	Udaipur	30	2		451 (420-481)
Tamil Nadu	Ariyalur	2			22 (17-26)
Tamil Nadu	Chennai	258	9		6551 (5664-7437)
Tamil Nadu	Coimbatore	19			478 (445-511)
Tamil Nadu	Cuddalore	55	5		1239 (1116-1363)
Tamil Nadu	Dharmapuri	20	3	75	589 (547-630)

State	Distict	Number of Hot-Spots	Number of Network Operators	Villages	Estimates
**Tamil Nadu**	**Dindigul**	9		70	329 (306-353)
**Tamil Nadu**	**Erode**	32	6	126	748 (688-807)
**Tamil Nadu**	**Kanchipuram**	74	4		1681 (1485-1877)
**Tamil Nadu**	**Kanniyakumari**	46			1232 (1145-1319)
**Tamil Nadu**	**Karur**	70	5		1833 (1567-2099)
**Tamil Nadu**	**Krishnagiri**	12	4		238 (204-272)
**Tamil Nadu**	**Madurai**	47			675 (560-790)
**Tamil Nadu**	**Nagapattinam**	35	3		1039 (918-1160)
**Tamil Nadu**	**Namakkal**	17	15		448 (409-488)
**Tamil Nadu**	**Perambalur**	35	8		697 (605-788)
**Tamil Nadu**	**Pudukkottai**	35	2		380 (304-456)
**Tamil Nadu**	**Ramanathapuram**	54	1	26	1244 (1120-1369)
**Tamil Nadu**	**Salem**	148	33	211	3516 (3237-3795)
**Tamil Nadu**	**Sivaganga**	44			835 (727-942)
**Tamil Nadu**	**Thanjavur**	50	2	41	1383 (1140-1626)
**Tamil Nadu**	**The Nilgiris**			4	8 (8-8)
**Tamil Nadu**	**Theni**	10	2	36	187 (160-213)
**Tamil Nadu**	**Thiruvallur**	65	5	129	1675 (1512-1838)
**Tamil Nadu**	**Thiruvarur**	7			198 (163-233)
**Tamil Nadu**	**Tiruchirappalli**	86	13		1816 (1527-2105)
**Tamil Nadu**	**Tirunelveli**	74	5	156	2357 (2179-2534)
**Tamil Nadu**	**Tiruppur**	38	5		458 (352-564)
**Tamil Nadu**	**Tiruvannamalai**	52	5	50	1367 (1299-1436)
**Tamil Nadu**	**Tuticorin**	46			905 (786-1025)
**Tamil Nadu**	**Vellore**	59	7	91	1132 (970-1295)
**Tamil Nadu**	**Villupuram**	80	6	98	2087 (1984-2190)
**Tamil Nadu**	**Virudhunagar**	50			938 (784-1093)
**Telangana**	**Adilabad**	24		26	870 (780-959)
**Telangana**	**Bhadradri Kothagudem**			5	11 (11-11)

State	Distict	Number of Hot-Spots	Number of Network Operators	Villages	Estimates
Telangana	Hanumakonda			16	15 (15-15)
Telangana	Hyderabad	67	14		5139 (5023-5255)
Telangana	Jagitial			17	5 (5-5)
Telangana	Jangoan			10	11 (11-11)
Telangana	Jayashankar Bhoopalpally			17	25 (25-25)
Telangana	Kamareddy		2	1	2 (2-2)
Telangana	Karimnagar	61	1	1	2167 (2017-2317)
Telangana	Khammam	27	15	17	801 (724-879)
Telangana	Komaram Bheem Asifabad			8	11 (11-11)
Telangana	Mahabubabad			28	32 (32-32)
Telangana	Mahbubnagar	3	1		94 (81-107)
Telangana	Mancherial			26	37 (37-37)
Telangana	Medak	9			125 (113-137)
Telangana	Mulug			22	36 (36-36)
Telangana	Nagarkurnool			1	1 (1-1)
Telangana	Nalgonda	28	5		2032 (1794-2270)
Telangana	Nirmal			42	72 (72-72)
Telangana	Nizamabad	28	15	12	1964 (1796-2131)
Telangana	Peddapalli			2	2 (2-2)
Telangana	Rajanna Sircilla			22	67 (67-67)
Telangana	Rangareddy	1		1	50 (46-54)
Telangana	Suryapet			1	4 (4-4)
Telangana	Wanaparthy			3	2 (2-2)
Telangana	Warangal	44	23	45	2852 (2574-3131)
Tripura	Dhalai	6			30 (27-33)
Tripura	Gomati	13			67 (60-74)
Tripura	Khowai	6	1		40 (36-44)
Tripura	North Tripura	45	1	33	349 (328-370)
Tripura	Sepahijala	3		3	19 (18-21)

State	Distict	Number of Hot-Spots	Number of Network Operators	Villages	Estimates
**Tripura**	**South Tripura**	14	1		78 (66-89)
**Tripura**	**Unakoti**	6		15	35 (32-37)
**Tripura**	**West Tripura**	33	1	10	258 (241-275)
**Uttar Pradesh**	**Agra**	10			239 (224-253)
**Uttar Pradesh**	**Aligarh**	19	2		339 (327-350)
**Uttar Pradesh**	**Allahabad**	5			60 (46-73)
**Uttar Pradesh**	**Ambedkar Nagar**	6	3		54 (48-60)
**Uttar Pradesh**	**Amroha**	31	5		264 (231-298)
**Uttar Pradesh**	**Auraiya**	20			97 (88-106)
**Uttar Pradesh**	**Azamgarh**	16	4		395 (361-428)
**Uttar Pradesh**	**Bahraich**	4			90 (84-95)
**Uttar Pradesh**	**Ballia**	11	3		236 (216-255)
**Uttar Pradesh**	**Balrampur**	6			54 (48-60)
**Uttar Pradesh**	**Banda**	12			201 (178-223)
**Uttar Pradesh**	**Bareilly**	99			645 (576-714)
**Uttar Pradesh**	**Basti**	9		76	245 (239-252)
**Uttar Pradesh**	**Bhadohi**	1			22 (19-24)
**Uttar Pradesh**	**Budaun**	11			216 (190-241)
**Uttar Pradesh**	**Bulandshahr**	64			644 (610-678)
**Uttar Pradesh**	**Chandauli**	19			175 (144-205)
**Uttar Pradesh**	**Deoria**	15			222 (209-234)
**Uttar Pradesh**	**Etah**	12			269 (262-277)
**Uttar Pradesh**	**Etawah**	27			310 (294-325)
**Uttar Pradesh**	**Faizabad**	16			105 (88-121)
**Uttar Pradesh**	**Farrukhabad**	17			168 (152-184)
**Uttar Pradesh**	**Fatehpur**	13	4		182 (147-217)
**Uttar Pradesh**	**Firozabad**	17			196 (180-211)
**Uttar Pradesh**	**Gautam Buddha Nagar**	19			275 (258-291)
**Uttar Pradesh**	**Ghaziabad**	45			451 (359-543)

State	Distict	Number of Hot-Spots	Number of Network Operators	Villages	Estimates
**Uttar Pradesh**	**Ghazipur**	38	7		399 (326-471)
**Uttar Pradesh**	**Gonda**	2	1		15 (14-16)
**Uttar Pradesh**	**Gorakhpur**	20		58	188 (179-198)
**Uttar Pradesh**	**Hamirpur**	8			123 (116-131)
**Uttar Pradesh**	**Hapur**	39			507 (464-550)
**Uttar Pradesh**	**Hardoi**	29			597 (522-672)
**Uttar Pradesh**	**Hathras**	22			276 (255-297)
**Uttar Pradesh**	**Jalaun**	3			23 (21-24)
**Uttar Pradesh**	**Jaunpur**	17		22	342 (310-373)
**Uttar Pradesh**	**Jhansi**	19			291 (275-307)
**Uttar Pradesh**	**Kannauj**	25			230 (213-248)
**Uttar Pradesh**	**Kanpur Dehat**	15			162 (148-175)
**Uttar Pradesh**	**Kanpur Nagar**	81	5		1347 (1241-1452)
**Uttar Pradesh**	**Kasganj**	32	3		437 (409-465)
**Uttar Pradesh**	**Kaushambi**	1	1		15 (14-15)
**Uttar Pradesh**	**Kheri**	20	1		132 (120-144)
**Uttar Pradesh**	**Kushi Nagar**	26		5	431 (409-453)
**Uttar Pradesh**	**Lalitpur**	19			273 (245-301)
**Uttar Pradesh**	**Lucknow**	99	17	12	1955 (1759-2151)
**Uttar Pradesh**	**Maharajganj**	18			258 (241-275)
**Uttar Pradesh**	**Mainpuri**	19			191 (176-205)
**Uttar Pradesh**	**Mathura**	20			194 (168-219)
**Uttar Pradesh**	**Mau**	40	8		569 (457-681)
**Uttar Pradesh**	**Meerut**	12	1		61 (49-74)
**Uttar Pradesh**	**Moradabad**	39	2	19	356 (319-393)
**Uttar Pradesh**	**Muzaffarnagar**	26			308 (256-359)
**Uttar Pradesh**	**Pilibhit**	18			322 (289-354)
**Uttar Pradesh**	**Rae Bareli**	14	3		287 (259-315)
**Uttar Pradesh**	**Rampur**	20			219 (199-238)

State	Distict	Number of Hot-Spots	Number of Network Operators	Villages	Estimates
Uttar Pradesh	Saharanpur	24	2		207 (186-228)
Uttar Pradesh	Sambhal	24	2		169 (157-181)
Uttar Pradesh	Sant Kabeer Nagar	1			16 (14-18)
Uttar Pradesh	Shahjahanpur	36			296 (267-324)
Uttar Pradesh	Shamli	16			184 (161-207)
Uttar Pradesh	Siddharth Nagar	13	7	24	373 (347-399)
Uttar Pradesh	Sitapur	28	6		588 (545-631)
Uttar Pradesh	Sonbhadra	8	3		55 (42-68)
Uttar Pradesh	Sultanpur	13			274 (237-310)
Uttar Pradesh	Unnao	27	7		744 (699-788)
Uttar Pradesh	Varanasi	13			109 (90-128)
Uttarakhand	Champawat	3			41 (32-50)
Uttarakhand	Dehradun	46			815 (737-894)
Uttarakhand	Haridwar	59	8		937 (888-986)
Uttarakhand	Nainital	16			311 (284-337)
Uttarakhand	Pauri Garhwal	27			183 (172-195)
Uttarakhand	Pithoragarh	3	2		44 (37-50)
Uttarakhand	Rudra Prayag	4			13 (11-14)
Uttarakhand	Tehri Garhwal	3			11 (10-12)
Uttarakhand	Udam Singh Nagar	26	3		524 (473-575)
Uttarakhand	Uttar Kashi	1			5 (4-5)
West Bengal	24 Paraganas North	10	6	2	401 (379-422)
West Bengal	24 Paraganas South	11		28	169 (154-184)
West Bengal	Basirhat	1			12 (11-12)
West Bengal	Birbhum	2	1	1	87 (85-89)
West Bengal	Coochbehar	4			56 (51-61)
West Bengal	Darjeeling	27	9	10	444 (421-466)
West Bengal	Diamond Harbour	5		18	57 (51-63)
West Bengal	Dinajpur Dakshin	1			6 (5-6)

State	Distict	Number of Hot-Spots	Number of Network Operators	Villages	Estimates
**West Bengal**	**Dinajpur Uttar**	10	2		180 (157-203)
**West Bengal**	**Hooghly**	23		1	398 (358-437)
**West Bengal**	**Howrah**	5			110 (101-119)
**West Bengal**	**Jalpaiguri**	8		3	106 (93-119)
**West Bengal**	**Kalimpong**			2	2 (2-2)
**West Bengal**	**Kolkata**	28	1		579 (509-649)
**West Bengal**	**Maldah**	4			78 (68-87)
**West Bengal**	**Medinipur East**	3			108 (101-115)
**West Bengal**	**Murshidabad**	2			24 (19-29)
**West Bengal**	**Nadia**	5			107 (100-115)
**West Bengal**	**Paschim Bardhaman**	16	11		471 (435-507)
**West Bengal**	**Purba Bardhaman**	2	1	3	84 (82-85)
**West Bengal**	**Rampurhat**	3		1	55 (51-59)
