# Supplementary material for: Programmatic mapping and population size estimation of key population in India: Method and findings
Source: PLOS Glob Public Health. 2025 May 7;5(5):e0004475. doi: 10.1371/journal.pgph.0004475 (PMC12057993; doi:10.1371/journal.pgph.0004475)
Supplement: S5 Table — (PDF) [file pgph.0004475.s011.pdf]

Supplementary Table S5. State/UT-wise size estimates of PWID (At hotspots, exclusively with network operators, exclusively in LWS villages) and adult men

State/UT	PWID Estimates			Adult Men Size
	At hotspots	Exclusively with network operators	Exclusively in LWS villages	
Andhra Pradesh	1,292	40	-	1,48,92,000
Arunachal Pradesh	5,143	-	-	4,26,895
Assam	25,293	862	-	99,11,002
Bihar	4,235	186	165	3,29,30,001
Chandigarh	1,871	37	-	3,97,081
Chhattisgarh	3,718	199	6	80,59,000
Delhi	31,743	738	-	65,01,001
Goa	302	-	-	4,54,140
Gujarat	779	-	-	2,04,18,002
Haryana	18,412	613	-	88,80,001
Himachal Pradesh	3,650	12	-	21,02,000
Jammu And Kashmir	8,797	1,365	-	40,78,000
Jharkhand	779	-	-	1,06,76,000
Karnataka	4,015	316	-	1,91,39,001
Kerala	3,017	258	-	86,84,001
Madhya Pradesh	10,808	1,103	8	2,35,85,000
Maharashtra	1,072	17	7	3,68,96,000
Manipur	23,198	107	1,679	8,65,449
Meghalaya	2,962	-	213	8,27,274
Mizoram	9,871	-	526	3,32,204
Nagaland	16,448	354	-	6,14,887
Odisha	4,094	426	6	1,24,99,001
Puducherry	21	-	-	4,29,251
Punjab	35,045	2,049	8,004	91,15,001
Rajasthan	2,839	184	-	2,21,25,000
Sikkim	821	-	-	2,11,982
Tamil Nadu	114	-	1	2,08,06,000
Telangana	816	-	-	1,09,27,000
Tripura	5,885	277	337	11,69,296
Uttar Pradesh	34,375	911	126	6,53,23,000
Uttarakhand	3,835	-	-	33,14,000
West Bengal	2,083	123	128	2,82,68,001
India	2,67,333	10,178	11,206	38,48,56,471
