# Supplementary material for: Programmatic mapping and population size estimation of key population in India: Method and findings
Source: PLOS Glob Public Health. 2025 May 7;5(5):e0004475. doi: 10.1371/journal.pgph.0004475 (PMC12057993; doi:10.1371/journal.pgph.0004475)
Supplement: S6 Table — (PDF) [file pgph.0004475.s012.pdf]

Supplementary Table S3. District-wise size estimates of PWID, PMPSE 2020-22

State	Distict	Number of Hot-Spots	Number of Network Operators	Villages	Estimates
Andhra Pradesh	Krishna	61	1		664 (553-775)
Andhra Pradesh	Spsr Nellore	26			277 (204-350)
Andhra Pradesh	Visakhapatanam	60			392 (304-480)
Arunachal Pradesh	Changlang	16			210 (187-232)
Arunachal Pradesh	East Kameng	15			237 (212-261)
Arunachal Pradesh	East Siang	59			956 (867-1045)
Arunachal Pradesh	Leparada	6			47 (40-54)
Arunachal Pradesh	Lohit	9			78 (67-89)
Arunachal Pradesh	Lower Dibang Valley	2			11 (9-12)
Arunachal Pradesh	Lower Siang	9			88 (78-98)
Arunachal Pradesh	Lower Subansiri	10			149 (134-163)
Arunachal Pradesh	Namsai	73			799 (742-856)
Arunachal Pradesh	Papum Pare	109			1282 (1175-1390)
Arunachal Pradesh	Siang	3			29 (25-33)
Arunachal Pradesh	Tirap	6			98 (90-105)
Arunachal Pradesh	Upper Siang	9			78 (71-84)
Arunachal Pradesh	Upper Subansiri	28			312 (266-357)
Arunachal Pradesh	West Kameng	17			265 (244-287)
Arunachal Pradesh	West Siang	36			506 (471-541)
Assam	Barpeta	38			998 (875-1121)
Assam	Cachar	96			1659 (1458-1860)
Assam	Chirang	2			30 (25-35)
Assam	Darrang	18			287 (245-329)
Assam	Dhemaji	79	3		999 (897-1100)
Assam	Dhubri	12	1		239 (214-264)
Assam	Dibrugarh	179			2645 (2145-3144)
Assam	Dima Hasao	11	1		341 (320-362)

State	Distict	Number of Hot-Spots	Number of Network Operators	Villages	Estimates
Assam	East Karbi Anglong	66	3		1265 (1036-1494)
Assam	Goalpara	24	2		508 (448-569)
Assam	Golaghat	36	4		1637 (1482-1792)
Assam	Hailakandi	37			839 (739-938)
Assam	Hojai	11			169 (145-193)
Assam	Kamrup	17			892 (687-1098)
Assam	Kamrup Metro	256	67		5763 (5110-6415)
Assam	Lakhimpur	24			418 (356-480)
Assam	Marigaon	10			588 (555-621)
Assam	Nagaon	54	17		1983 (1847-2119)
Assam	Nalbari	54			1545 (1464-1626)
Assam	Sivasagar	44			1473 (1321-1624)
Assam	Sonitpur	47	7		913 (738-1087)
Assam	Tinsukia	16			845 (756-935)
Assam	Udalguri	14			121 (101-141)
Bihar	Aurangabad	6			180 (166-195)
Bihar	Begusarai	15	2	4	300 (273-327)
Bihar	Bhojpur	17			326 (302-350)
Bihar	Buxar	13	4		329 (320-337)
Bihar	Darbhanga			14	22 (22-22)
Bihar	Kaimur (Bhabua)	38	7		829 (749-909)
Bihar	Lakhisarai	16	1		251 (225-278)
Bihar	Nalanda	26	3		361 (336-385)
Bihar	Patna	59	2		1348 (1254-1443)
Bihar	Purbi Champaran	1			16 (14-17)
Bihar	Purnia	1			10 (9-11)
Bihar	Rohtas	8			228 (209-247)
Bihar	Saran			41	49 (49-49)
Bihar	Sheikhpura	2			42 (37-46)

State	Distict	Number of Hot-Spots	Number of Network Operators	Villages	Estimates
Bihar	Sitamarhi	1		61	109 (109-109)
Bihar	Siwan	11	2		177 (168-186)
Bihar	Vaishali	1			9 (8-10)
Chandigarh	Chandigarh	82	6		1908 (1701-2115)
Chhattisgarh	Bilaspur	92	10	15	1634 (1507-1761)
Chhattisgarh	Durg	19			280 (220-339)
Chhattisgarh	Gaurela-Pendra-Marwahi	7	2		94 (87-100)
Chhattisgarh	Janjgir-Champa	1			15 (13-16)
Chhattisgarh	Korba	23	6		506 (434-578)
Chhattisgarh	Korea	30	3		570 (508-631)
Chhattisgarh	Raigarh	11			232 (210-254)
Chhattisgarh	Raipur	1			5 (4-5)
Chhattisgarh	Surajpur	34	1		534 (503-564)
Chhattisgarh	Surguja	4			55 (49-61)
Delhi	Central	195			3884 (3432-4335)
Delhi	East	56	6		1989 (1858-2121)
Delhi	New Delhi	43			864 (788-940)
Delhi	North	85			2108 (1865-2350)
Delhi	North East	143	10		4290 (3766-4814)
Delhi	North West	124	3		3378 (3013-3743)
Delhi	Shahdara	123	1		3075 (2785-3364)
Delhi	South	119			3324 (3086-3561)
Delhi	South East	183			4847 (4460-5233)
Delhi	South West	47			1430 (1362-1497)
Delhi	West	131	2		3295 (3032-3558)
Goa	North Goa	13			229 (199-260)
Goa	South Goa	9			72 (48-97)
Gujarat	Ahmadabad	29			258 (214-302)
Gujarat	Surat	33			364 (290-438)

State	Distict	Number of Hot-Spots	Number of Network Operators	Villages	Estimates
Gujarat	Vadodara	21			157 (133-181)
Haryana	Ambala	106	6		1017 (891-1143)
Haryana	Bhiwani	3			22 (19-25)
Haryana	Faridabad	91			1268 (1152-1384)
Haryana	Fatehabad	159			1975 (1774-2175)
Haryana	Gurugram	21			366 (334-398)
Haryana	Hisar	239	34		2685 (2389-2981)
Haryana	Jhajjar	51			521 (467-576)
Haryana	Jind	49			474 (392-556)
Haryana	Kaithal	72	3		760 (653-867)
Haryana	Karnal	29			397 (339-455)
Haryana	Kurukshetra	8	2		208 (186-230)
Haryana	Mewat	39			829 (726-932)
Haryana	Palwal	33			410 (368-452)
Haryana	Panchkula	39	3		471 (436-506)
Haryana	Panipat	48			631 (577-685)
Haryana	Rewari	11			244 (236-253)
Haryana	Rohtak	111			1334 (1227-1440)
Haryana	Sirsa	302	17		4286 (4030-4543)
Haryana	Sonipat	80	8		549 (483-615)
Haryana	Yamunanagar	44			579 (516-642)
Himachal Pradesh	Bilaspur_HP	11			152 (138-165)
Himachal Pradesh	Chamba	8			104 (90-117)
Himachal Pradesh	Hamirpur	16			116 (104-127)
Himachal Pradesh	Kangra	33			443 (385-501)
Himachal Pradesh	Kullu	26	4		267 (236-299)
Himachal Pradesh	Mandi	11			73 (63-83)
Himachal Pradesh	Shimla	84			737 (611-863)
Himachal Pradesh	Sirmaur	12			293 (255-331)

State	Distict	Number of Hot-Spots	Number of Network Operators	Villages	Estimates
Himachal Pradesh	Solan	38			763 (681-845)
Himachal Pradesh	Una	107			714 (650-778)
Jammu And Kashmir	Anantnag	56	7		832 (601-1063)
Jammu And Kashmir	Badgam	15	5		289 (198-380)
Jammu And Kashmir	Bandipora	16	2		246 (151-341)
Jammu And Kashmir	Baramulla	60	7		982 (667-1297)
Jammu And Kashmir	Doda	23	6		378 (322-435)
Jammu And Kashmir	Ganderbal	12	4		212 (140-285)
Jammu And Kashmir	Jammu	64	20		1169 (975-1363)
Jammu And Kashmir	Kathua	65	24		1247 (1143-1350)
Jammu And Kashmir	Kishtwar	7	2		137 (114-159)
Jammu And Kashmir	Kulgam	64	2		704 (612-797)
Jammu And Kashmir	Kupwara	25	3		421 (260-582)
Jammu And Kashmir	Poonch	9	7		208 (189-226)
Jammu And Kashmir	Pulwama	23	5		337 (267-407)
Jammu And Kashmir	Rajauri	22	10		406 (354-459)
Jammu And Kashmir	Reasi	19	5		350 (301-400)
Jammu And Kashmir	Samba	16	4		370 (321-419)
Jammu And Kashmir	Shopian	44	3		592 (517-667)
Jammu And Kashmir	Srinagar	50	8		782 (470-1093)
Jammu And Kashmir	Udhampur	27	9		501 (422-580)
Jharkhand	Bokaro	25			308 (241-375)
Jharkhand	East Singhbum	19			268 (237-299)
Jharkhand	Lohardaga	1			7 (5-8)
Jharkhand	Ranchi	16			197 (159-234)
Karnataka	Bengaluru Urban	411			2730 (2295-3166)
Karnataka	Kolar	67	12		1598 (1474-1722)
Karnataka	Tumakuru	1			2 (2-3)
Kerala	Alappuzha	9			43 (36-51)

State	Distict	Number of Hot-Spots	Number of Network Operators	Villages	Estimates
Kerala	Ernakulam	67			719 (604-833)
Kerala	Kannur	20			240 (211-268)
Kerala	Kollam	11			134 (89-179)
Kerala	Kozhikode	98	11		1022 (909-1135)
Kerala	Malappuram	62			315 (271-359)
Kerala	Thiruvananthapuram	103	5		803 (508-1097)
Madhya Pradesh	Anuppur	10	1		90 (77-103)
Madhya Pradesh	Ashoknagar	26	2		206 (189-224)
Madhya Pradesh	Bhind	2	2		23 (22-25)
Madhya Pradesh	Bhopal	95	14		1651 (1523-1779)
Madhya Pradesh	Chhatarpur	40	4		607 (543-670)
Madhya Pradesh	Datia	1	1		13 (12-14)
Madhya Pradesh	Dhar	1			14 (11-16)
Madhya Pradesh	Guna	22	7		561 (445-678)
Madhya Pradesh	Gwalior	20	3		378 (302-455)
Madhya Pradesh	Hoshangabad	45	17		856 (779-934)
Madhya Pradesh	Indore	5			18 (14-21)
Madhya Pradesh	Jabalpur	70	15	1	1793 (1571-2014)
Madhya Pradesh	Jhabua	1			32 (29-34)
Madhya Pradesh	Katni	17			169 (149-189)
Madhya Pradesh	Mandsaur	3	1		34 (30-37)
Madhya Pradesh	Narsinghpur	10			181 (163-199)
Madhya Pradesh	Neemuch	4			13 (9-16)
Madhya Pradesh	Panna	18			357 (298-417)
Madhya Pradesh	Rajgarh	1			18 (15-20)
Madhya Pradesh	Ratlam	31	4		376 (335-417)
Madhya Pradesh	Rewa	127	11		1608 (1420-1796)
Madhya Pradesh	Satna	32	2		454 (385-524)
Madhya Pradesh	Sehore	16	4		346 (307-384)

State	Distict	Number of Hot-Spots	Number of Network Operators	Villages	Estimates
Madhya Pradesh	Shahdol	25	1		271 (232-309)
Madhya Pradesh	Sheopur	47			496 (404-588)
Madhya Pradesh	Shivpuri	4			128 (116-139)
Madhya Pradesh	Sidhi	49	6		446 (369-523)
Madhya Pradesh	Singrauli	17	1		249 (221-277)
Madhya Pradesh	Ujjain	32	5		462 (421-503)
Madhya Pradesh	Umaria	2	1		20 (17-23)
Madhya Pradesh	Vidisha	11			53 (39-66)
Maharashtra	Jalgaon	1			11 (9-12)
Maharashtra	Pune	15			177 (130-224)
Maharashtra	Thane	2			170 (134-205)
Maharashtra	Washim			2	7 (7-7)
Manipur	Bishnupur	138		71	2542 (2252-2833)
Manipur	Chandel	58		42	874 (763-984)
Manipur	Churachandpur	60		92	1525 (1170-1879)
Manipur	Imphal East	295	7	75	5693 (4615-6771)
Manipur	Imphal West	303		95	4437 (3311-5563)
Manipur	Jiribam	27		12	406 (314-499)
Manipur	Kakching	119		16	1818 (1428-2207)
Manipur	Kamjong	42		43	424 (302-547)
Manipur	Kangpokpi	47		58	808 (686-931)
Manipur	Pherzawl			15	86 (86-86)
Manipur	Senapati	19		36	515 (384-645)
Manipur	Tamenglong	30		120	618 (541-694)
Manipur	Tengnoupal	71		35	1379 (1111-1648)
Manipur	Thoubal	86	7	80	2297 (2085-2510)
Manipur	Ukhrul	114		56	1563 (1399-1727)
Meghalaya	East Jaintia Hills	26		22	621 (560-682)
Meghalaya	East Khasi Hills	57			1550 (1344-1756)

State	Distict	Number of Hot-Spots	Number of Network Operators	Villages	Estimates
Meghalaya	Ri Bhoi	7			232 (171-293)
Meghalaya	South West Khasi Hills	1			48 (45-50)
Meghalaya	West Garo Hills	12			130 (101-158)
Meghalaya	West Jaintia Hills	28		7	310 (250-369)
Meghalaya	West Khasi Hills	11			285 (244-326)
Mizoram	Aizawl	323		11	4301 (3559-5042)
Mizoram	Champhai	64		39	1392 (1256-1527)
Mizoram	Kolasib	39		19	1080 (1004-1157)
Mizoram	Lawngtlai	23		6	457 (411-504)
Mizoram	Lunglei	81		32	1234 (1091-1377)
Mizoram	Mamit	55		42	843 (716-970)
Mizoram	Saiha	33		12	590 (466-713)
Mizoram	Serchhip	35		11	501 (424-577)
Maharashtra	Mumbai	46	4		732 (630-834)
Nagaland	Mon	125			2629 (2089-3170)
Nagaland	Dimapur	212	1		2645 (2125-3165)
Nagaland	Kiphire	34			275 (240-310)
Nagaland	Kohima	106			1390 (1120-1661)
Nagaland	Longleng	44			761 (707-815)
Nagaland	Mokokchung	145	31		2699 (2303-3095)
Nagaland	Noklak	42			501 (449-552)
Nagaland	Peren	68			808 (664-953)
Nagaland	Phek	98			1020 (890-1149)
Nagaland	Tuensang	146	4		2231 (2029-2433)
Nagaland	Wokha	121			1122 (931-1313)
Nagaland	Zunheboto	53			721 (590-851)
Odisha	Anugul	2			13 (12-14)
Odisha	Balangir	3		9	12 (11-13)
Odisha	Bargarh	32	3		611 (574-648)

State	Distict	Number of Hot-Spots	Number of Network Operators	Villages	Estimates
Odisha	Bhadrak	2			10 (8-12)
Odisha	Cuttack	103	2		736 (564-907)
Odisha	Dhenkanal	4			53 (47-59)
Odisha	Jagatsinghapur	4			28 (19-36)
Odisha	Jajapur	4			33 (27-40)
Odisha	Jharsuguda	7			30 (21-38)
Odisha	Kendrapara	1			3 (2-3)
Odisha	Khordha	106	14		1422 (1193-1650)
Odisha	Koraput	1			12 (12-12)
Odisha	Nabarangpur	3			26 (24-27)
Odisha	Puri	79	27		825 (775-875)
Odisha	Sambalpur	30			529 (471-587)
Odisha	Sundargarh	20			185 (158-211)
Puducherry	Mahe	8			21 (13-28)
Punjab	Amritsar	182	4	145	5236 (4669-5802)
Punjab	Barnala	17	4		133 (74-191)
Punjab	Bathinda	29	3		593 (448-739)
Punjab	Faridkot	78		100	1115 (1054-1175)
Punjab	Fatehgarh Sahib	54	5		1251 (1119-1383)
Punjab	Firozepur	57		100	2262 (2175-2349)
Punjab	Gurdaspur	104	30		2871 (2439-3303)
Punjab	Hoshiarpur	137	23		2249 (1837-2661)
Punjab	Jalandhar	290	6		4983 (4251-5715)
Punjab	Kapurthala	84	21		1782 (1547-2017)
Punjab	Ludhiana	127	8	105	3789 (3375-4202)
Punjab	Mansa	117			1794 (1580-2009)
Punjab	Moga	85	5	100	3685 (3515-3855)
Punjab	Nawanshahr	29	3		829 (743-914)
Punjab	Pathankot	35	20		1472 (1385-1559)

State	Distict	Number of Hot-Spots	Number of Network Operators	Villages	Estimates
Punjab	Patiala	103	1		1395 (1207-1583)
Punjab	Rupnagar	43	3		1082 (984-1180)
Punjab	S.A.S Nagar	33	5		828 (752-904)
Punjab	Sangrur	36	2		457 (387-527)
Punjab	Sri Muktsar Sahib	42	11	92	902 (862-943)
Punjab	Tarn Taran	233	8	100	6392 (5922-6861)
Rajasthan	Ajmer	31			492 (411-572)
Rajasthan	Alwar	2			14 (12-16)
Rajasthan	Banswara	1	1		3 (3-4)
Rajasthan	Bharatpur	12			232 (214-249)
Rajasthan	Bhilwara	6	1		29 (24-34)
Rajasthan	Bikaner	19			204 (176-231)
Rajasthan	Bundi	7	1		42 (37-46)
Rajasthan	Chittorgarh	3	2		30 (28-31)
Rajasthan	Dholpur	12			20 (15-25)
Rajasthan	Dungarpur	1	1		32 (29-34)
Rajasthan	Ganganagar	25	4		354 (323-385)
Rajasthan	Hanumangarh	18	2		271 (247-294)
Rajasthan	Jaipur	7	3		232 (220-243)
Rajasthan	Jaisalmer	1			11 (10-11)
Rajasthan	Jalore	3	1		7 (6-8)
Rajasthan	Jhalawar	3			8 (6-10)
Rajasthan	Jhunjhunu	1			10 (9-11)
Rajasthan	Jodhpur	6	2		70 (61-79)
Rajasthan	Karauli	3			37 (31-42)
Rajasthan	Kota	42	4		756 (696-817)
Rajasthan	Nagaur	4			26 (19-32)
Rajasthan	Pali	4			30 (26-34)
Rajasthan	Rajsamand	4	1		10 (8-11)

State	Distict	Number of Hot-Spots	Number of Network Operators	Villages	Estimates
Rajasthan	Sawai Madhopur	3	3		68 (64-72)
Rajasthan	Sikar	4			13 (11-14)
Rajasthan	Sirohi	1			23 (21-25)
Rajasthan	Udaipur	1			4 (3-4)
Sikkim	East District	38			507 (435-579)
Sikkim	South District	20			314 (278-350)
Tamil Nadu	Thanjavur	1			9 (4-14)
Tamil Nadu	Tiruchirappalli	3			5 (3-6)
Tamil Nadu	Tirunelveli			1	1 (1-1)
Tamil Nadu	Tuticorin	10			101 (84-117)
Telangana	Hyderabad	64			816 (657-974)
Tripura	Dhalai	116			1074 (997-1150)
Tripura	Gomati	58			572 (494-649)
Tripura	Khowai	30	4	24	344 (322-366)
Tripura	North Tripura	66	1	51	929 (855-1002)
Tripura	Sepahijala	52	5		502 (470-534)
Tripura	South Tripura	109	9		867 (780-954)
Tripura	Unakoti	56		50	582 (544-621)
Tripura	West Tripura	166	15	19	1630 (1490-1771)
Uttar Pradesh	Agra	81	3		1153 (1045-1262)
Uttar Pradesh	Aligarh	34			433 (411-454)
Uttar Pradesh	Allahabad	83	11		1834 (1530-2137)
Uttar Pradesh	Ambedkar Nagar	12			184 (158-211)
Uttar Pradesh	Amroha	41	6		340 (298-383)
Uttar Pradesh	Auraiya	32			247 (231-264)
Uttar Pradesh	Azamgarh	12			309 (283-334)
Uttar Pradesh	Bahraich	108			2324 (2160-2487)
Uttar Pradesh	Ballia	38	2		517 (443-591)
Uttar Pradesh	Balrampur	10			149 (140-158)

State	Distict	Number of Hot-Spots	Number of Network Operators	Villages	Estimates
Uttar Pradesh	Banda	19			239 (210-267)
Uttar Pradesh	Bareilly	44			482 (450-513)
Uttar Pradesh	Basti	15		108	316 (288-343)
Uttar Pradesh	Bhadohi	2			49 (44-54)
Uttar Pradesh	Budaun	5			113 (101-125)
Uttar Pradesh	Bulandshahr	26			422 (405-438)
Uttar Pradesh	Chandauli	22			293 (244-341)
Uttar Pradesh	Chitrakoot	5			55 (50-60)
Uttar Pradesh	Deoria	16			243 (227-260)
Uttar Pradesh	Etah	37			473 (438-509)
Uttar Pradesh	Etawah	18			245 (236-254)
Uttar Pradesh	Faizabad	43			495 (445-545)
Uttar Pradesh	Farrukhabad	28			358 (322-393)
Uttar Pradesh	Fatehpur	21			671 (601-742)
Uttar Pradesh	Firozabad	15			299 (280-318)
Uttar Pradesh	Gautam Buddha Nagar	8			79 (72-86)
Uttar Pradesh	Ghaziabad	62			1359 (1210-1508)
Uttar Pradesh	Ghazipur	32	2		609 (481-738)
Uttar Pradesh	Gonda	40	4		615 (570-660)
Uttar Pradesh	Gorakhpur	81		22	1814 (1593-2035)
Uttar Pradesh	Hamirpur	10			226 (217-234)
Uttar Pradesh	Hapur	29			312 (283-340)
Uttar Pradesh	Hardoi	32			574 (527-621)
Uttar Pradesh	Hathras	23			277 (257-296)
Uttar Pradesh	Jalaun	31			456 (426-486)
Uttar Pradesh	Jaunpur	41		3	345 (304-387)
Uttar Pradesh	Jhansi	20			115 (104-127)
Uttar Pradesh	Kannauj	11			141 (128-153)
Uttar Pradesh	Kanpur Dehat	24			276 (254-297)

State	Distict	Number of Hot-Spots	Number of Network Operators	Villages	Estimates
Uttar Pradesh	Kanpur Nagar	108			1631 (1468-1793)
Uttar Pradesh	Kasganj	33	3		479 (457-501)
Uttar Pradesh	Kaushambi	31	12		443 (410-476)
Uttar Pradesh	Kheri	123	8		1111 (1009-1213)
Uttar Pradesh	Kushi Nagar	26		4	350 (327-373)
Uttar Pradesh	Lucknow	99		12	2587 (2325-2849)
Uttar Pradesh	Maharajganj	10			138 (130-146)
Uttar Pradesh	Mahoba	22	2		288 (249-327)
Uttar Pradesh	Mainpuri	28			249 (234-263)
Uttar Pradesh	Mathura	15			153 (131-174)
Uttar Pradesh	Mau	10			184 (140-227)
Uttar Pradesh	Meerut	45	5		550 (437-664)
Uttar Pradesh	Moradabad	196	12	49	1697 (1444-1950)
Uttar Pradesh	Muzaffarnagar	35			395 (352-437)
Uttar Pradesh	Pilibhit	42			619 (573-665)
Uttar Pradesh	Pratapgarh	13			111 (105-117)
Uttar Pradesh	Rae Bareli	12			201 (187-214)
Uttar Pradesh	Rampur	77			799 (687-912)
Uttar Pradesh	Saharanpur	22	2		209 (190-228)
Uttar Pradesh	Sambhal	17			120 (107-132)
Uttar Pradesh	Sant Kabeer Nagar	15			234 (194-273)
Uttar Pradesh	Shahjahanpur	65	2		508 (453-562)
Uttar Pradesh	Shamli	17			208 (185-230)
Uttar Pradesh	Siddharth Nagar	10	4	13	305 (291-320)
Uttar Pradesh	Sitapur	32			623 (571-675)
Uttar Pradesh	Sonbhadra	18	8		604 (495-712)
Uttar Pradesh	Sultanpur	14	1		359 (323-394)
Uttar Pradesh	Unnao	23			568 (513-623)
Uttar Pradesh	Varanasi	22			254 (216-292)

State	Distict	Number of Hot-Spots	Number of Network Operators	Villages	Estimates
Uttarakhand	Champawat	18			121 (76-166)
Uttarakhand	Dehradun	32			753 (699-807)
Uttarakhand	Haridwar	38			721 (643-799)
Uttarakhand	Nainital	35			889 (816-961)
Uttarakhand	Rudra Prayag	1			11 (9-13)
Uttarakhand	Tehri Garhwal	13			83 (69-98)
Uttarakhand	Udam Singh Nagar	68	10		1219 (1063-1376)
Uttarakhand	Uttar Kashi	8			38 (34-43)
West Bengal	24 Paraganas South	1		10	18 (17-19)
West Bengal	Birbhum			3	30 (30-30)
West Bengal	Darjeeling	69	15	19	1272 (1078-1465)
West Bengal	Diamond Harbour			17	23 (23-23)
West Bengal	Howrah	18	2		397 (362-433)
West Bengal	Jalpaiguri	4			109 (102-116)
West Bengal	Kalimpong	4		4	73 (65-81)
West Bengal	Kolkata	16			278 (220-335)
West Bengal	Murshidabad	7			124 (90-158)
West Bengal	Rampurhat	1			10 (9-11)
