# Supplementary material for: Programmatic mapping and population size estimation of key population in India: Method and findings
Source: PLOS Glob Public Health. 2025 May 7;5(5):e0004475. doi: 10.1371/journal.pgph.0004475 (PMC12057993; doi:10.1371/journal.pgph.0004475)
Supplement: S7 Table — (PDF) [file pgph.0004475.s013.pdf]

Supplementary Table S7. State/UT-wise size estimates of H/TG people (At hotspots, exclusively with network operators, exclusively in LWS villages) and adult men

State/UT	H/TG people estimate			Adult Men Size
	At hotspots	Exclusively with network operators	Exclusively in LWS villages	
Andhra Pradesh	5,253	62	49	1,48,92,000
Arunachal Pradesh	140	-	-	4,26,895
Assam	2,246	235	-	99,11,002
Bihar	756	89	1	3,29,30,001
Chandigarh	155	9	-	3,97,081
Chhattisgarh	1,082	9	27	80,59,000
Delhi	16,166	1,741	-	65,01,001
Goa	119	13	-	4,54,140
Gujarat	2,436	-	169	2,04,18,002
Haryana	1,410	25	-	88,80,001
Himachal Pradesh	251	7	-	21,02,000
Jammu And Kashmir	551	63	-	40,78,000
Jharkhand	483	-	-	1,06,76,000
Karnataka	10,017	894	15	1,91,39,001
Kerala	2,597	7	-	86,84,001
Madhya Pradesh	1,183	425	6	2,35,85,000
Maharashtra	9,360	698	265	3,68,96,000
Manipur	468	-	-	8,65,449
Meghalaya	67	42	1	8,27,274
Mizoram	-	-	-	3,32,204
Nagaland	82	-	-	6,14,887
Odisha	6,550	459	200	1,24,99,001
Puducherry	203	-	-	4,29,251
Punjab	1,228	85	3	91,15,001
Rajasthan	1,855	271	-	2,21,25,000
Sikkim	-	-	-	2,11,982
Tamil Nadu	8,287	827	97	2,08,06,000
Telangana	787	193	15	1,09,27,000
Tripura	41	118	-	11,69,296
Uttar Pradesh	8,937	894	15	6,53,23,000
Uttarakhand	319	2	-	33,14,000
West Bengal	4,357	600	178	2,82,68,001
India	87,385	7,767	1,041	38,48,56,471
