# Supplementary material for: Programmatic mapping and population size estimation of key population in India: Method and findings
Source: PLOS Glob Public Health. 2025 May 7;5(5):e0004475. doi: 10.1371/journal.pgph.0004475 (PMC12057993; doi:10.1371/journal.pgph.0004475)
Supplement: S8 Table — (PDF) [file pgph.0004475.s014.pdf]

Supplementary Table S4. District-wise size estimates of H/TG people, PMPSE 2020-22

State	Distict	Number of Hot-Spots	Number of Network Operators	Villages	Estimates
Andhra Pradesh	Anantapur	12	3		181 (164-197)
Andhra Pradesh	Annamayya	3			22 (20-25)
Andhra Pradesh	Bapatla	4			52 (46-58)
Andhra Pradesh	East Godavari	4			62 (51-74)
Andhra Pradesh	Eluru	5			105 (91-118)
Andhra Pradesh	Guntur	5		9	76 (66-86)
Andhra Pradesh	Kakinada	32	4	2	622 (542-701)
Andhra Pradesh	Konaseema	2			37 (32-42)
Andhra Pradesh	Krishna	30		6	368 (317-419)
Andhra Pradesh	Kurnool	55		29	1457 (1200-1713)
Andhra Pradesh	Nandyal	22			917 (695-1139)
Andhra Pradesh	Palnadu	3			126 (105-148)
Andhra Pradesh	Prakasam	3	1	3	75 (66-84)
Andhra Pradesh	Spsr Nellore	11		2	221 (186-255)
Andhra Pradesh	Sri Satya Sai	1			31 (26-35)
Andhra Pradesh	Srikakulam	11		1	125 (102-148)
Andhra Pradesh	Visakhapatanam	39			632 (540-723)
Andhra Pradesh	Vizianagaram	7			99 (76-122)
Andhra Pradesh	West Godavari	5	1		66 (48-83)
Andhra Pradesh	Y.S.R.	11		1	91 (44-139)
Arunachal Pradesh	East Siang	7			42 (38-47)
Arunachal Pradesh	Namsai	3			9 (7-11)
Arunachal Pradesh	Papum Pare	41			88 (67-110)
Assam	Barpeta	19			111 (93-128)
Assam	Bongaigaon	2			16 (14-18)
Assam	Cachar	2			6 (3-8)
Assam	Darrang	3			43 (36-51)

State	Distict	Number of Hot-Spots	Number of Network Operators	Villages	Estimates
Assam	Dhubri	1	1		22 (20-24)
Assam	East Karbi Anglong	8	3		109 (95-124)
Assam	Goalpara	2			65 (57-72)
Assam	Golaghat	4			19 (17-21)
Assam	Hojai	2			39 (35-43)
Assam	Kamrup	10			61 (47-74)
Assam	Kamrup Metro	127	36		1440 (1249-1631)
Assam	Nagaon	4	2		108 (101-114)
Assam	Nalbari	6	1		303 (289-317)
Assam	Sivasagar	3			93 (89-97)
Assam	Sonitpur	2			33 (26-39)
Assam	Tinsukia	2			16 (13-18)
Bihar	Aurangabad	2			19 (16-21)
Bihar	Begusarai	8			64 (57-70)
Bihar	Bhojpur	3	1		80 (77-83)
Bihar	Buxar	8	3		136 (126-145)
Bihar	Darbhanga	3		1	13 (11-15)
Bihar	Kaimur (Bhabua)	3	1		50 (39-61)
Bihar	Lakhisarai	1			17 (16-18)
Bihar	Nalanda	2			26 (25-27)
Bihar	Patna	2			55 (52-58)
Bihar	Purbi Champaran	4			51 (47-56)
Bihar	Purnia	1	1		22 (18-25)
Bihar	Rohtas	2			31 (28-35)
Bihar	Saran	3	3		31 (30-32)
Bihar	Sheikhpura	1			11 (10-12)
Bihar	Sheohar		1		7 (7-7)
Bihar	Sitamarhi			1	1 (1-1)
Bihar	Siwan	10	2		165 (153-177)

State	Distict	Number of Hot-Spots	Number of Network Operators	Villages	Estimates
Bihar	Vaishali	5			68 (61-74)
Chandigarh	Chandigarh	15	1		164 (142-185)
Chhattisgarh	Balod	6			42 (38-46)
Chhattisgarh	Baloda Bazar	2			14 (12-15)
Chhattisgarh	Bastar	10			81 (65-97)
Chhattisgarh	Bemetara	2			18 (17-20)
Chhattisgarh	Bilaspur	14	3	7	138 (114-161)
Chhattisgarh	Dhamtari	3			55 (51-59)
Chhattisgarh	Durg	23		2	219 (204-234)
Chhattisgarh	Gaurela-Pendra-Marwahi	1			4 (3-4)
Chhattisgarh	Janjgir-Champa	1			8 (6-9)
Chhattisgarh	Kanker	6			73 (68-78)
Chhattisgarh	Korba	1			4 (2-5)
Chhattisgarh	Korea	1	1		32 (31-32)
Chhattisgarh	Raigarh	2			48 (45-51)
Chhattisgarh	Raipur	20		22	260 (228-292)
Chhattisgarh	Rajnandgaon	8	1	2	106 (90-122)
Chhattisgarh	Surguja	2			18 (16-20)
Delhi	Central	73	3		1400 (1280-1520)
Delhi	East	44	2		856 (777-935)
Delhi	New Delhi	72			1291 (1115-1466)
Delhi	North	60	10		1877 (1731-2023)
Delhi	North East	33			1144 (1049-1239)
Delhi	North West	88	4		2034 (1792-2275)
Delhi	Shahdara	16			352 (324-379)
Delhi	South	54			1186 (1014-1358)
Delhi	South East	64			1103 (934-1272)
Delhi	South West	40	1		2403 (2237-2570)
Delhi	West	123	36		4262 (3935-4589)

State	Distict	Number of Hot-Spots	Number of Network Operators	Villages	Estimates
Goa	North Goa	6			95 (81-108)
Goa	South Goa	3	2		37 (35-40)
Gujarat	Ahmadabad	15		15	218 (187-249)
Gujarat	Amreli	1		18	24 (23-24)
Gujarat	Anand	2		8	51 (46-55)
Gujarat	Arvalli			1	8 (8-8)
Gujarat	Banas Kantha	2		8	31 (28-33)
Gujarat	Bharuch	3			47 (42-52)
Gujarat	Bhavnagar	2		26	44 (42-46)
Gujarat	Devbhumi Dwarka	5			20 (19-21)
Gujarat	Dohad	1			18 (11-24)
Gujarat	Gandhinagar	6		11	77 (64-90)
Gujarat	Jamnagar	6			14 (12-17)
Gujarat	Kachchh	8		25	109 (92-125)
Gujarat	Kheda	1			6 (6-7)
Gujarat	Mahesana	1		18	33 (32-34)
Gujarat	Mahisagar	1			8 (7-10)
Gujarat	Narmada	1			9 (7-10)
Gujarat	Navsari	2			29 (25-32)
Gujarat	Porbandar	2			11 (9-13)
Gujarat	Rajkot	7			92 (83-102)
Gujarat	Sabar Kantha			4	14 (14-14)
Gujarat	Surat	32			1351 (1273-1430)
Gujarat	Surendranagar	14			72 (58-85)
Gujarat	Vadodara	5			321 (308-334)
Haryana	Ambala	2			13 (12-14)
Haryana	Faridabad	63			661 (588-734)
Haryana	Gurugram	12			120 (104-137)
Haryana	Hisar	12			65 (56-74)

State	Distict	Number of Hot-Spots	Number of Network Operators	Villages	Estimates
Haryana	Jhajjar	5	2		71 (66-75)
Haryana	Jind	2			25 (23-27)
Haryana	Kaithal	6			48 (39-56)
Haryana	Karnal	2			27 (23-30)
Haryana	Kurukshetra		2		8 (8-8)
Haryana	Palwal	7			87 (72-101)
Haryana	Panipat	10	2		75 (68-81)
Haryana	Rewari	4			64 (61-67)
Haryana	Rohtak	1			9 (8-10)
Haryana	Sirsa	21			129 (116-141)
Haryana	Yamunanagar	4			36 (32-40)
Himachal Pradesh	Bilaspur_HP	4			26 (22-29)
Himachal Pradesh	Chamba	2			21 (18-24)
Himachal Pradesh	Hamirpur	3	1		44 (39-49)
Himachal Pradesh	Kangra	6			55 (46-64)
Himachal Pradesh	Kullu	2			15 (13-17)
Himachal Pradesh	Mandi	2			9 (8-10)
Himachal Pradesh	Shimla	4			22 (20-23)
Himachal Pradesh	Sirmaur	2			40 (33-47)
Himachal Pradesh	Solan	3			15 (13-16)
Himachal Pradesh	Una	5			12 (11-13)
Jammu And Kashmir	Badgam	4			14 (11-16)
Jammu And Kashmir	Ganderbal	1			4 (3-5)
Jammu And Kashmir	Jammu	16	5		228 (200-255)
Jammu And Kashmir	Kathua	5	1		63 (57-69)
Jammu And Kashmir	Poonch	1			26 (24-29)
Jammu And Kashmir	Rajauri	2	1		42 (37-47)
Jammu And Kashmir	Samba	3			55 (50-61)
Jammu And Kashmir	Srinagar	13			171 (140-201)

State	Distict	Number of Hot-Spots	Number of Network Operators	Villages	Estimates
Jammu And Kashmir	Udhampur	1			12 (9-14)
Jharkhand	Bokaro	12			82 (70-93)
Jharkhand	Chatra	1			7 (7-8)
Jharkhand	Dhanbad	6			60 (51-68)
Jharkhand	East Singhbum	8			138 (124-153)
Jharkhand	Giridih	12			134 (119-148)
Jharkhand	Hazaribagh	2			37 (34-40)
Jharkhand	Koderma	1			14 (6-22)
Jharkhand	West Singhbhum	1			11 (10-11)
Karnataka	Bagalkot	12	1	5	356 (333-379)
Karnataka	Ballari	48	9	6	506 (478-534)
Karnataka	Belagavi	7	1	7	162 (146-178)
Karnataka	Bengaluru Rural	61			342 (300-384)
Karnataka	Bengaluru Urban	307			4034 (3692-4376)
Karnataka	Bidar	7	2		188 (179-197)
Karnataka	Chamarajanagar	8	2		117 (108-125)
Karnataka	Chikballapur	7			46 (36-55)
Karnataka	Chikkamagaluru	6	4		88 (80-96)
Karnataka	Chitradurga	19	7		353 (330-376)
Karnataka	Dakshin Kannad	5	8		129 (118-140)
Karnataka	Davangere	4	8		142 (133-151)
Karnataka	Dharwad	2			13 (12-15)
Karnataka	Gadag	4			71 (57-85)
Karnataka	Haveri	2	1		92 (88-96)
Karnataka	Kalaburagi	50	2		872 (778-966)
Karnataka	Kolar	55	50		917 (839-994)
Karnataka	Koppal	20	6		261 (236-286)
Karnataka	Mandya	12			154 (131-177)
Karnataka	Mysuru	34			243 (195-290)

State	Distict	Number of Hot-Spots	Number of Network Operators	Villages	Estimates
Karnataka	Raichur	54	3		575 (502-649)
Karnataka	Ramanagara	10	3		110 (101-118)
Karnataka	Shivamogga	6			43 (35-51)
Karnataka	Tumakuru	46			326 (245-406)
Karnataka	Udupi	4			18 (15-22)
Karnataka	Uttar Kannad	8			46 (29-64)
Karnataka	Vijayapura	17		5	304 (283-325)
Karnataka	Yadgir	45	8		418 (355-481)
Kerala	Alappuzha	6			33 (28-38)
Kerala	Kannur	24			266 (223-309)
Kerala	Kasaragod	13			154 (135-173)
Kerala	Kollam	56			593 (550-635)
Kerala	Kottayam	36			329 (274-384)
Kerala	Kozhikode	18			277 (220-333)
Kerala	Malappuram	36			263 (237-288)
Kerala	Thiruvananthapuram	45			337 (195-478)
Kerala	Thrissur	39	1		352 (305-398)
Madhya Pradesh	Alirajpur	2	1		14 (9-19)
Madhya Pradesh	Anuppur		1		6 (6-6)
Madhya Pradesh	Ashoknagar	2			5 (4-5)
Madhya Pradesh	Bhind		1		3 (3-3)
Madhya Pradesh	Bhopal	5	2		252 (232-272)
Madhya Pradesh	Damoh	1			2 (1-2)
Madhya Pradesh	Datia	13	3		109 (95-124)
Madhya Pradesh	East Nimar	1			32 (24-39)
Madhya Pradesh	Guna	1			15 (14-16)
Madhya Pradesh	Gwalior	11	1		92 (72-112)
Madhya Pradesh	Indore	44	11		491 (432-551)
Madhya Pradesh	Jabalpur	7	1		197 (183-212)

State	Distict	Number of Hot-Spots	Number of Network Operators	Villages	Estimates
Madhya Pradesh	Jhabua	1			6 (5-7)
Madhya Pradesh	Katni	2	1		33 (30-35)
Madhya Pradesh	Khargone	2			5 (4-5)
Madhya Pradesh	Morena	8			44 (35-52)
Madhya Pradesh	Neemuch	3			14 (10-17)
Madhya Pradesh	Ratlam	4			37 (32-42)
Madhya Pradesh	Rewa	1	1		33 (32-34)
Madhya Pradesh	Sagar	4	2		44 (41-47)
Madhya Pradesh	Satna	1	1		38 (34-42)
Madhya Pradesh	Sehore	1			4 (3-5)
Madhya Pradesh	Shahdol	1	1		3 (2-5)
Madhya Pradesh	Sheopur	1			11 (10-12)
Madhya Pradesh	Shivpuri	2			9 (6-11)
Madhya Pradesh	Sidhi	1	1		10 (7-12)
Madhya Pradesh	Singrauli	1	2		8 (8-9)
Madhya Pradesh	Tikamgarh	4	3	1	27 (25-30)
Madhya Pradesh	Ujjain	4	3	3	63 (57-68)
Madhya Pradesh	Umaria	1	1		10 (9-10)
Maharashtra	Ahmednagar	7			192 (176-207)
Maharashtra	Amravati	1	4		160 (158-162)
Maharashtra	Aurangabad	5			171 (171-172)
Maharashtra	Buldhana	1			23 (20-25)
Maharashtra	Chandrapur	4	2	9	152 (146-158)
Maharashtra	Gondia	3			55 (52-58)
Maharashtra	Jalgaon	50			273 (239-307)
Maharashtra	Kolhapur	35	3	16	329 (261-396)
Maharashtra	Latur	5	4		110 (100-119)
Maharashtra	Nagpur	12	4	2	532 (479-584)
Maharashtra	Nanded	13	12	4	311 (290-332)

State	Distict	Number of Hot-Spots	Number of Network Operators	Villages	Estimates
Maharashtra	Nashik	9			126 (109-143)
Maharashtra	Osmanabad	27	3		227 (195-259)
Maharashtra	Pune	34		69	547 (474-620)
Maharashtra	Raigad	21			271 (231-310)
Maharashtra	Sangli	31	3	47	194 (180-207)
Maharashtra	Satara	16		32	236 (212-261)
Maharashtra	Solapur	23			303 (260-346)
Maharashtra	Thane	99	5		2455 (2102-2808)
Maharashtra	Washim			4	4 (4-4)
Manipur	Bishnupur	17			181 (158-205)
Manipur	Imphal West	42			287 (237-336)
Meghalaya	East Khasi Hills	5	5		92 (74-111)
Meghalaya	Ri Bhoi	3			17 (10-23)
Meghalaya	West Jaintia Hills			1	1 (1-1)
Maharashtra	Mumbai	81	6		3655 (3390-3919)
Nagaland	Dimapur	12			60 (44-76)
Nagaland	Mokokchung	1			22 (18-26)
Odisha	Anugul	57	3		1046 (927-1165)
Odisha	Balangir	62	1	29	291 (238-344)
Odisha	Baleshwar	13			123 (110-136)
Odisha	Bargarh	4	1		63 (60-66)
Odisha	Bhadrak	17	2		174 (148-199)
Odisha	Boudh	2			40 (38-42)
Odisha	Cuttack	39	17		471 (418-524)
Odisha	Deogarh	7			124 (117-131)
Odisha	Dhenkanal	44	3		335 (291-379)
Odisha	Gajapati	21	2		171 (146-196)
Odisha	Ganjam	61	12	118	503 (447-559)
Odisha	Jagatsinghapur	15			143 (86-201)

State	Distict	Number of Hot-Spots	Number of Network Operators	Villages	Estimates
Odisha	Jajapur	17	2		143 (124-162)
Odisha	Jharsuguda	7			40 (30-50)
Odisha	Kalahandi	52	3	27	321 (251-392)
Odisha	Kandhamal	10	1		97 (84-111)
Odisha	Kendrapara	4	1		31 (27-35)
Odisha	Kendujhar	43			403 (330-477)
Odisha	Khordha	44	12		561 (510-612)
Odisha	Koraput	24	2		134 (113-154)
Odisha	Malkangiri	8	1		87 (75-99)
Odisha	Mayurbhanj	17	2		205 (189-222)
Odisha	Nabarangpur	11			62 (52-71)
Odisha	Nayagarh	19	5		156 (143-168)
Odisha	Nuapada	3		1	22 (20-24)
Odisha	Puri	31	19		419 (380-459)
Odisha	Rayagada	35	2		440 (392-488)
Odisha	Sambalpur	13			139 (121-156)
Odisha	Sonepur	5			64 (56-72)
Odisha	Sundargarh	32			399 (353-444)
Puducherry	Karaikal	8			20 (15-26)
Puducherry	Mahe	5			52 (44-59)
Puducherry	Pondicherry	8			131 (118-145)
Punjab	Amritsar	14			136 (108-164)
Punjab	Barnala	1			8 (7-9)
Punjab	Faridkot	2			5 (3-6)
Punjab	Firozepur	6		3	163 (152-174)
Punjab	Gurdaspur		1		2 (2-2)
Punjab	Jalandhar	8			69 (56-82)
Punjab	Kapurthala	1			17 (13-20)
Punjab	Ludhiana	23	6	1	554 (508-600)

State	Distict	Number of Hot-Spots	Number of Network Operators	Villages	Estimates
Punjab	Mansa	6			33 (29-37)
Punjab	Pathankot	1			10 (8-12)
Punjab	Patiala	13			160 (137-183)
Punjab	Rupnagar	1			6 (6-6)
Punjab	S.A.S Nagar	3			42 (36-47)
Punjab	Sangrur	1	1		24 (21-26)
Punjab	Sri Muktsar Sahib	1			6 (5-7)
Punjab	Tarn Taran	9		5	83 (76-90)
Rajasthan	Ajmer	11	4		175 (158-193)
Rajasthan	Alwar	4	2		35 (32-37)
Rajasthan	Banswara	1	1		4 (4-5)
Rajasthan	Bharatpur	2	1		14 (12-15)
Rajasthan	Bhilwara	3	1		12 (11-14)
Rajasthan	Bikaner	1			5 (5-5)
Rajasthan	Bundi	1			3 (2-3)
Rajasthan	Chittorgarh	6	3		49 (46-52)
Rajasthan	Churu	1			25 (20-30)
Rajasthan	Dholpur	5			105 (94-116)
Rajasthan	Dungarpur	6	1		39 (35-43)
Rajasthan	Ganganagar	28	4		274 (239-309)
Rajasthan	Hanumangarh	19	3		150 (121-179)
Rajasthan	Jaipur	61	14		660 (577-743)
Rajasthan	Jaisalmer	1			6 (5-6)
Rajasthan	Jalore	3	1		6 (5-8)
Rajasthan	Jhalawar	2			3 (2-4)
Rajasthan	Jhunjhunu	2	1		44 (40-49)
Rajasthan	Jodhpur	1	2		17 (15-19)
Rajasthan	Karauli	2	3		52 (51-52)
Rajasthan	Kota	7	4		121 (107-134)

State	Distict	Number of Hot-Spots	Number of Network Operators	Villages	Estimates
Rajasthan	Nagaur	1			6 (6-7)
Rajasthan	Pali	10	1		105 (97-114)
Rajasthan	Pratapgarh	3			10 (8-11)
Rajasthan	Rajsamand	5	1		20 (18-22)
Rajasthan	Sawai Madhopur	2	2		17 (16-18)
Rajasthan	Sikar	1			3 (3-3)
Rajasthan	Sirohi	1			19 (18-20)
Rajasthan	Tonk	3	1		23 (21-25)
Rajasthan	Udaipur	10	1		124 (116-131)
Tamil Nadu	Chennai	85	17		1677 (1529-1826)
Tamil Nadu	Coimbatore	15			219 (192-246)
Tamil Nadu	Cuddalore	14	2		288 (259-318)
Tamil Nadu	Dharmapuri	17	2	1	236 (210-263)
Tamil Nadu	Dindigul	4			128 (119-138)
Tamil Nadu	Erode	12	2	89	194 (181-208)
Tamil Nadu	Kanchipuram	52	5		782 (566-999)
Tamil Nadu	Kanniyakumari	10			175 (163-188)
Tamil Nadu	Karur	5	2		155 (135-175)
Tamil Nadu	Krishnagiri	14	7		272 (232-312)
Tamil Nadu	Madurai	21			203 (140-265)
Tamil Nadu	Nagapattinam	15	1		263 (181-345)
Tamil Nadu	Namakkal	31	1		381 (315-446)
Tamil Nadu	Perambalur	6			37 (30-43)
Tamil Nadu	Pudukkottai	3			21 (17-25)
Tamil Nadu	Ramanathapuram	16	1		61 (51-71)
Tamil Nadu	Salem	46	2	1	476 (390-563)
Tamil Nadu	Sivaganga	3			18 (15-21)
Tamil Nadu	Thanjavur	30	1		382 (246-518)
Tamil Nadu	Theni	5		1	50 (44-57)

State	Distict	Number of Hot-Spots	Number of Network Operators	Villages	Estimates
Tamil Nadu	Thiruvallur	45	5	29	727 (644-811)
Tamil Nadu	Thiruvarur	5			66 (54-77)
Tamil Nadu	Tiruchirappalli	23	1		289 (225-354)
Tamil Nadu	Tirunelveli	38	3	12	309 (268-349)
Tamil Nadu	Tiruppur	21	3		155 (123-187)
Tamil Nadu	Tiruvannamalai	19	1	1	210 (189-231)
Tamil Nadu	Tuticorin	16			276 (242-309)
Tamil Nadu	Vellore	31	3		467 (397-537)
Tamil Nadu	Villupuram	32			318 (277-358)
Tamil Nadu	Virudhunagar	43			375 (304-446)
Telangana	Adilabad		1	3	4 (4-4)
Telangana	Hyderabad	2	6		343 (341-346)
Telangana	Karimnagar	7			54 (44-64)
Telangana	Khammam	4		2	117 (98-135)
Telangana	Mancherial			3	2 (2-2)
Telangana	Medak	1			20 (18-22)
Telangana	Nalgonda	12	9		224 (201-247)
Telangana	Nirmal		1	2	3 (3-3)
Telangana	Suryapet			1	6 (6-6)
Telangana	Warangal	6	1	2	222 (202-243)
Tripura	Dhalai		1		12 (12-12)
Tripura	Gomati		2		20 (20-20)
Tripura	Khowai	1			3 (3-3)
Tripura	South Tripura		1		72 (72-72)
Tripura	West Tripura	8	1		52 (47-57)
Uttar Pradesh	Agra	4			29 (24-33)
Uttar Pradesh	Aligarh	3			57 (56-58)
Uttar Pradesh	Allahabad	1			45 (35-55)
Uttar Pradesh	Ambedkar Nagar	7	3		74 (63-85)

State	Distict	Number of Hot-Spots	Number of Network Operators	Villages	Estimates
Uttar Pradesh	Amroha	23	4		203 (180-227)
Uttar Pradesh	Auraiya	20			151 (141-160)
Uttar Pradesh	Azamgarh	15	5		234 (216-252)
Uttar Pradesh	Bahraich	4			45 (42-48)
Uttar Pradesh	Ballia	42	4		331 (283-378)
Uttar Pradesh	Balrampur	5	1		38 (34-42)
Uttar Pradesh	Banda	3			59 (55-63)
Uttar Pradesh	Bareilly	78			553 (503-603)
Uttar Pradesh	Basti	2			22 (18-25)
Uttar Pradesh	Bhadohi	2			34 (29-38)
Uttar Pradesh	Budaun	5			86 (78-93)
Uttar Pradesh	Bulandshahr	19			171 (162-181)
Uttar Pradesh	Chandauli	5			20 (17-22)
Uttar Pradesh	Chitrakoot	2			8 (6-9)
Uttar Pradesh	Deoria	5			61 (52-69)
Uttar Pradesh	Etah	8			113 (105-122)
Uttar Pradesh	Etawah	11			72 (67-77)
Uttar Pradesh	Faizabad	5			29 (26-32)
Uttar Pradesh	Farrukhabad	3			46 (43-49)
Uttar Pradesh	Fatehpur	2	1		44 (37-50)
Uttar Pradesh	Firozabad	10			36 (31-42)
Uttar Pradesh	Gautam Buddha Nagar	20			250 (220-280)
Uttar Pradesh	Ghaziabad	49	12		727 (614-841)
Uttar Pradesh	Ghazipur	6	1		25 (20-30)
Uttar Pradesh	Gonda	3	1		27 (26-28)
Uttar Pradesh	Gorakhpur	19			207 (194-220)
Uttar Pradesh	Hamirpur	8			50 (45-55)
Uttar Pradesh	Hapur	17			160 (145-175)
Uttar Pradesh	Hardoi	19			80 (68-92)

State	Distict	Number of Hot-Spots	Number of Network Operators	Villages	Estimates
Uttar Pradesh	Hathras	3			16 (15-18)
Uttar Pradesh	Jalaun	10			67 (60-75)
Uttar Pradesh	Jaunpur	6	1		51 (42-59)
Uttar Pradesh	Jhansi	13			113 (105-120)
Uttar Pradesh	Kannauj	12			159 (152-166)
Uttar Pradesh	Kanpur Dehat	19			88 (78-98)
Uttar Pradesh	Kanpur Nagar	89			932 (864-1001)
Uttar Pradesh	Kasganj	9	2		109 (102-116)
Uttar Pradesh	Kaushambi	1			5 (5-5)
Uttar Pradesh	Kheri	20			72 (69-74)
Uttar Pradesh	Kushi Nagar	13		2	151 (141-161)
Uttar Pradesh	Lalitpur	6			88 (79-96)
Uttar Pradesh	Lucknow	32	4		379 (317-440)
Uttar Pradesh	Maharajganj	5			25 (21-29)
Uttar Pradesh	Mahoba	2	1		71 (65-77)
Uttar Pradesh	Mainpuri	6			52 (47-57)
Uttar Pradesh	Mathura	5			37 (33-40)
Uttar Pradesh	Mau	17			88 (74-103)
Uttar Pradesh	Meerut	23	1		118 (92-144)
Uttar Pradesh	Mirzapur	1			5 (4-5)
Uttar Pradesh	Moradabad	36	10	9	398 (372-424)
Uttar Pradesh	Muzaffarnagar	46	25		764 (734-795)
Uttar Pradesh	Pilibhit	9			88 (80-95)
Uttar Pradesh	Pratapgarh	1	1		89 (85-93)
Uttar Pradesh	Rae Bareli	8	1		135 (126-145)
Uttar Pradesh	Rampur	23			252 (227-278)
Uttar Pradesh	Saharanpur	9	2		98 (91-105)
Uttar Pradesh	Sambhal	18			86 (78-94)
Uttar Pradesh	Sant Kabeer Nagar	1			17 (14-20)

State	Distict	Number of Hot-Spots	Number of Network Operators	Villages	Estimates
Uttar Pradesh	Shahjahanpur	20			120 (107-133)
Uttar Pradesh	Shamli	13	13		108 (99-117)
Uttar Pradesh	Siddharth Nagar	10	7	4	259 (238-280)
Uttar Pradesh	Sitapur	6	4		90 (78-102)
Uttar Pradesh	Sonbhadra	4	1		21 (17-26)
Uttar Pradesh	Sultanpur	2			12 (10-14)
Uttar Pradesh	Unnao	4			110 (98-123)
Uttar Pradesh	Varanasi	17			491 (430-551)
Uttarakhand	Dehradun	9			88 (79-97)
Uttarakhand	Haridwar	5			111 (107-115)
Uttarakhand	Nainital	7			60 (53-66)
Uttarakhand	Pithoragarh	1	1		8 (6-9)
Uttarakhand	Tehri Garhwal	4			26 (22-30)
Uttarakhand	Udam Singh Nagar		1		2 (2-2)
Uttarakhand	Uttar Kashi	7			27 (24-30)
West Bengal	24 Paraganas North	33	1	4	542 (496-588)
West Bengal	24 Paraganas South	16		23	262 (213-311)
West Bengal	Alipurduar	5		10	137 (133-142)
West Bengal	Basirhat	1		2	8 (8-9)
West Bengal	Birbhum	4	1	2	78 (77-79)
West Bengal	Coochbehar	3	1		117 (111-122)
West Bengal	Darjeeling	3	1	3	85 (83-88)
West Bengal	Diamond Harbour	5		25	52 (48-56)
West Bengal	Dinajpur Dakshin	29			357 (342-371)
West Bengal	Dinajpur Uttar	32	1	4	494 (451-537)
West Bengal	Hooghly	11	2	6	308 (298-318)
West Bengal	Howrah	18			274 (255-294)
West Bengal	Jalpaiguri	8		9	119 (105-132)
West Bengal	Kalimpong		1		9 (9-9)

State	Distict	Number of Hot-Spots	Number of Network Operators	Villages	Estimates
West Bengal	Kolkata	46	13		825 (738-913)
West Bengal	Maldah	4			105 (98-113)
West Bengal	Medinipur East	8			263 (241-284)
West Bengal	Medinipur West	11		2	132 (127-138)
West Bengal	Murshidabad	17		3	215 (180-250)
West Bengal	Nadia	22			379 (356-401)
West Bengal	Paschim Bardhaman	2	6	3	150 (147-154)
West Bengal	Purba Bardhaman	4	6	2	171 (164-177)
West Bengal	Rampurhat	4			53 (52-54)
